# Time to recovery from Obstetric Fistula and its predictors among patients admitted to Hamlin Fistula Center, Addis Ababa, Ethiopia: A survival analysis

**DOI:** 10.1371/journal.pgph.0006848

**Published:** 2026-07-09

**Authors:** Hayat Ibrahim Oumer, Delelegn Yilma Gebremichael, Gizachew Abdissa Bulto

**Affiliations:** 1 Kolfe Keranio Woreda 06 Health Center, Addis Ababa City Health Bureau, Addis Ababa, Ethiopia; 2 Department of Public Health, College of Health Sciences and Referral Hospital, Ambo University, Ambo, Ethiopia; 3 Department of Midwifery, College of Health Sciences and Referral Hospital, Ambo University, Ambo, Ethiopia; PLOS: Public Library of Science, UNITED STATES OF AMERICA

## Abstract

Obstetric Fistula (OBF) remains a devastating maternal condition in Ethiopia, causing significant physical suffering, social isolation and psychological traumas among affected women. Despite its burden, only a limited number of healthcare facilities in Ethiopia provide surgical interventions. Furthermore, there is a scarcity of evidence regarding the time to recovery from OBF and its predictors in treatment centers. Therefore, this study aimed to determine the time to recovery from OBF and its predictors among patients admitted to Hamlin Fistula Hospital Addis Ababa, Ethiopia. A Hospital based retrospective cohort study was conducted among 513 women treated for OBF from January 2017 to June 2023. The Kaplan-Meier method was employed to estimate the distribution of recovery time, and the log-rank test was utilized to assess the experience of recovery time among different levels of categorical variables. Bivariate and multivariable Cox proportional regression analyses were conducted. The overall recovery rate was 74.7%, (95%CI: 71.1, 78.2), with a median recovery-time from OBF was 5.9 weeks (95%CI: 5.1, 6.6). Receiving physiotherapy after surgery (AHR: 1.85, 95%CI: 1.13–3.02) and having a fistula length of 2.2–4 cm (AHR: 5.23, 95%CI: 1.17–12.33) were found to be predictors of faster recovery for OBF. Conversely, women with urethral damage (AHR: 0.43, 95%CI: 0.25–0.76) or severe vaginal scarring (AHR: 0.60, 95%CI: 0.40–0.89) experienced delayed recovery from OBF. Only 7.3% of OBF patients received physiotherapy despite 85% faster recovery. Based on the findings, the median recovery time from OBF was 5.9 weeks. Therefore, scaling-up postoperative physiotherapy for all OBF patients, early identification, and providing counselling and specialized care for women with urethral injury or severe vaginal scarring could improve recovery time from OBF. These findings also highlighted the critical importance of individualized rehabilitation services in fistula treatment centers to optimize patient recovery and overall quality of life.

## Background

Obstetric Fistula (OBF) “is an abnormal opening or a hole between the vagina and urinary bladder or/and rectum or both that is resulted from prolonged and obstructed labor, surgical error, or trauma” [1,2]. Untreated, OBF leads to severe, lifetime morbidities with profound medical, psychological traumas and socio-economic consequences [3]. It is one of the most neglected human rights and public health problems, causing tremendous challenges such as emotional distress, social isolation, family abandonment, and stigma. As reported by Animut et al., women with OBF experience profound social isolation, divorce, health problems, and psychological trauma. One woman expressed, “I am a person who is standing but not alive. […] However, I understand one thing darkness surrounds us despite the sun keeps rising” (crying). The majority (91%) of affected women experience stillbirths, while many also face anxiety, depression, discrimination, and worsening poverty. Common physical complications include nerve damage, foot drop, urinary and fecal incontinence, infertility, renal failure, and other pelvic disorders [4–9].

OBF mainly occurs mainly in sub-Saharan Africa and Asia [10–12]. Without timely access to emergency caesarean section, women with obstructed labor face higher risks of developing OBF [3,13]. Each year, 50,000–100,000 women worldwide develop OBF. In Sub-Saharan Africa and Southeast Asia, up to 3.5 million women may live with untreated OBF, potentially more due to unreported cases [10]. In Ethiopia, the lifetime estimated risk of obstetric fistula in 2016 indicated that 72,533 women of reproductive age had experienced this condition. Of these, 31,961 cases remained untreated, whereas 40,572 have received treatment. Annually, 948 new cases of OBF were reported, constituting approximately 1.6% of total obstructed and prolonged labor cases and 0.03% of all deliveries in 2020. The incidence of untreated vaginal fistula (VF) symptoms was also estimated to be 1.4 cases per 1000 in Ethiopia [14,15]. Although the Ethiopia government’s strategic plan aims to eliminate OBF by 2025, it remained a significant contributor to maternal morbidity. The point prevalence of OBF is 1.4 per 1000 women of childbearing age [14]. The lifetime fistula prevalence was 3.4 cases per 1000 women of reproductive age in Ethiopia [15]. According to these reports, Ethiopia may not only fail to eliminate OBF by 2025 but also might be unable to achieve the Sustainable Development Goals of eradicating this condition by 2030 [16].

Although OBF is treatable through surgery, access remains limited due to a shortage of skilled professionals, essential resources, and financial or awareness barriers that prevent women from receiving care [5,17,18]. The recovery time from OBF in Ethiopia varies across hospitals, ranging from 18.71 days to 5.19 weeks. Factors influencing recovery include socio-demographic conditions, delays in seeking care, cultural and physical barriers, antibiotic use, surgeon experience, and fistula related factors. It is also influenced by hospital settings, treatment approaches, and patient conditions [13,19–24].

Although fistula centers have been established in Ethiopia and the government has implemented strategic initiatives targeting risk reduction, identification and treatment, reintegration, and income generation for survivors, with the goal of eradicating OBF by 2025, the condition remains prevalent [25]. While surgical repair can address fistulas in many women, complete recovery should involve more than just restoring continence [26]. A delay in the time to recovery from the OBF will affect the health care seeking behavior of women and the number of treated cases. Although a few studies on the survival time of OBF have been conducted in the Northern and Eastern Ethiopia, the recovery rate and median recovery time have not been well studied, and there is specifically no evidence from the country’s first and largest fistula center. Therefore, this study will assess the recovery rate and median recovery time and identify factors that predict the recovery time from OBF at Hamlin Fistula Center in Addis Ababa. This helps to understand factors contributing to the recovery time of OBF and to reduce morbidity and mortality due to its complications by improving the care provided in the country’s largest fistula hospital. This study also supports the national plan to eliminate OBF in Ethiopia. Therefore, this study assessed the time to recovery from OBF and its predictors at Addis Ababa Hamlin Fistula Hospital, Ethiopia.

## Methods

### Ethics statement

Ethical approval for this study was obtained from the Ethical Review Board of Ambo University, College of Health Sciences and Referral Hospital (reference number AU/CMHS-RCS/05/2023). A formal official letter of support was also sent to the Hamlin Fistula center. All study methods were performed in accordance with the relevant guidelines and regulations, and the study was performed in accordance with the Declaration of Helsinki. As the current study relied on data collected retrospectively from patient records, the requirement for the informed consent was waived by the Ethical Review Board of College of Health Sciences and Referral Hospital, Ambo University. After submitting the proposal and explaining the study’s purpose, benefits, and risks, permission and signed approval was obtained from the Hamlin Fistula Hospital’s administration. The study used existing patients’ records or charts. Recognizing the sensitivity of the information, the trained data collectors took the patients’ files to a separate room, where access was restricted to only the data collectors and the investigators. Once the data were collected, the records were placed back in their original place. We ensured patient confidentiality by removing personal identifiers, such as names, addresses, phone numbers, and names of healthcare providers. Additionally, the confidentiality of the data was ensured by using anonymous questionnaires and storing the collected information in a password-protected folder to which only researchers had access.

### Study design, area and period

A hospital based retrospective cohort study was conducted among patients treated for OBF at Hamlin Fistula Center, Ethiopia. The Hamlin Fistula Hospital Addis Ababa is the world’s first fistula hospital, founded by Dr. Catherine and Reg Hamlin in 1974. It is a charity organization established for the treatment and prevention of childbirth-related injuries in Ethiopia. The center treats around 200 patients each year using a surgical technique, and more than 50,000 patients were treated. Hamlin Fistula Ethiopia is a globally renowned center of excellence for treating OBF and training Midwives and specialties. The Hamlin Fistula Hospital Addis Ababa serves as Ethiopia’s national referral center, receiving complex cases including failed repairs, extensive tissue damage, and prolonged fistula duration from all regions. The facility offers comprehensive rehabilitation services, including physiotherapy, counselling, and skills training [27]. The study was conducted at the Hamlin fistula center from 27^th^ July to 22^nd^ August 2023.

### Population

Random samples of patients with OBF who were treated at the Hamlin fistula center, between 1^st^ January 2017 and 30^th^ June 2023 were included in the study population. The study included patients with OBF who received treatment at the Hamlin fistula center in Addis Ababa, provided their medical records were complete and contained both admission and discharge dates.

### Sample size determination and sampling technique

The sample size was determined using Open Epi considering the different assumptions: for a time to event comparison with a balanced ratio of unexposed to exposed (1:1), 95% confidence interval, 80% power and the anticipated proportion of the hazard of OBF recovery time among women. Damaged urethra (exposed) =20.7% and those with non-damaged urethral status (unexposed) =79.3%, and AHR = 1.59. The sample size was determined to be 417 for both the exposed and unexposed groups, resulting in a total of 834 participants. After accounting for a 10% non-response rate, the final sample size was found 918 [28].

Given that the available study population was limited to 945 individuals, which is below 10,000, we applied the finite population correction formula to adjust the sample size. By employing the standard correction formula: N_f_ = $\frac{n}{\text{1}+\left[\frac{n}{N}\right]}$, =$\frac{\text{9}\text{1}\text{8}}{\text{1}+\left[\frac{\text{9}\text{1}\text{8}}{\text{9}\text{4}\text{5}}\right]}$. We then calculated the final sample size as 466. After accounting for a 10% non-response rate due to missing values and incomplete patient records (as the data were collected from records), the final sample size was determined to be 466 × 1.10, which is approximately 513.

A simple random sampling technique was employed to select the study participants using the registration numbers of patients registered at the Hamlin fistula center. From a total of 945 patients with fistula enrolled between January 2017 and June 2023, 513 samples were chosen based on their medical record numbers in the database. The relevant information was then extracted from these selected records (S1 Fig).

### Study variables

Time to recovery from OBF in weeks is an outcome variable.

#### Predictor variables.

Socio-demographic factors included, women’s age, residence, level of education, marital status, body mass index, and age at marriage. Obstetric related factors which included, parity, place of delivery, delivery mode, and labor duration.

Fistula-related characteristics included the duration between the onset of the fistula and the time of surgical treatment, width and length of the fistula hole, pre- and postoperative care, repairing approach, type of fistula, duration of bladder catheterization, degree of vaginal scarring, presence of psychotherapy before and after surgery, and rehabilitation after surgery.

### Operational definitions

**Event:** recovering from obstetric fistula

**Recovered:** were those patients who became cured from obstetric fistula.

**Survival time:** The time period in weeks from the initiation of treatment for obstetric fistula to documented clinical recovery. Women were followed up from the start of treatment until recovery [29].

**Censoring:** a woman who does not experience the event(recovery from obstetric fistula) before the study ends, a person who died after being admitted for obstetric fistula treatment and patients whose recovery status was unknown at discharge [30].

**Iatrogenic fistula:** A fistula that is caused mostly by cesarean section and hysterectomy due to the complication of obstructed labor [31].

### Data collection tool and technique

Data for the current study were collected by using an English version of the checklist developed after reviewing the results of different articles [20,22,32]. The checklist was prepared in English, and data were collected electronically using the Kobo Toolbox. Secondary data from hospital patient files were used to collect data on OBF patients from the day of initial treatment for OBF to recovery at Hamlin fistula center. Two trained data collectors were used to collect data for the study.

### Data quality control and management

To enhance the quality and understandability of the data, training was provided for two midwife data collectors on how to use and collect data from the patient files. A pretest was performed on the records of 26 patients at Hamlin fistula center, Addis Ababa, among patients who were not considered in the final collected data. Accordingly, some modifications to the data extraction tool arrangements and the removal and addition of some questions were made based on the feedback from the pre-testing. The data collection process was stringently supervised by the investigators. The consistency and accuracy of the collected data were assessed daily.

### Data processing and analysis

Kobo Toolbox software was used for data collection, after which the data were exported to SPSS version 25 for statistical analyses. Data cleaning was performed to check for missing values and outliers. Women with incomplete records and data with significant missing values were excluded from the study, and we analyzed a complete case analysis. For categorical data, descriptive statistics included frequencies and percentages, whereas for continuous data, the mean, standard deviation, median, and interquartile range were calculated. Survival analysis was employed to determine the recovery time and predictors affecting the time to recover from OBF (S1 Data).

The distribution of recovery time was assessed using the Kaplan-Meier estimator, and the log-rank test was applied to assess the recovery time experience across different categories of categorical variables [33]. The model was tested using the log minus log. Then, Cox regression was done to identify the predictors of time to recovery for those variables that passed the model fitness. Cox-Snell residuals were used to assess the overall goodness of fit of the model. The proportional hazard assumptions were checked using a log min log plot and by checking for multicollinearity using VIF (1.323). Variables which were found to have a statistically significant association with a p-value < 0.25 in the bivariate Cox proportional hazard model analysis were then analyzed using a multivariable Cox proportional hazard model. The 95% confidence interval of the hazard ratio was calculated, and a variable with p-value less than or equal to 0.05 in the multivariable Cox proportional hazards model were considered to be significant predictors of the time to recovery from OBF.

## Results

### Socio-demographic characteristics

In this study, 513 patients’ cards were reviewed, and 495 patients were included, resulting in a response rate of 96.5%. About three-fifth 287 (58.0%) of the women were in the 19–30 years age group, with a mean age of 30.2 years with a standard deviation of 8.9 years. Additionally, 377 (76.2%) of the women were married, and nearly half 228 (46.1%) had given birth at or before the age of 18 years. In terms of education, 310 (62.6%) had no formal education. Half of them, 249 (50.3%), were engaged in farming, and many of them, 218 (44%), were from Oromia region (Table 1).

**Table 1:** Socio-demographic characteristics of obstetric fistula patients admitted at Hamlin fistula center Addis Ababa Ethiopia 2023 (N = 495).

Variables	Category	Frequency	Percent
Age of women	≤18 years	28	5.7
	19-30 years	287	58.0
	≥31 years	180	36.4
Age at first birth	≤18 age in years	228	46.1
	19-30 age in years	262	52.9
	≥31 age in years	5	1.0
Marital status	Married	377	76.2
	Separated	62	12.5
	Widowed and other	56	11.3
Educational status women	No formal Education	310	62.6
	Primary education (1–8)	127	25.7
	Secondary education (9–12)	33	6.7
	college and above	25	5.1
Region	Oromia	218	44.0
	Amhara	101	20.4
	SNNPR	94	19.0
	Addis Ababa and others	82	16.6
Occupation of the women	Farmer	249	50.3
	Housewife	173	34.9
	Governmental employees and others	73	14.7
BMI	Underweight	104	21.0
	Normal weight	323	65.3
	Overweight	68	13.7

### Obstetric related factors

Among the 495 women studied, 118(23.8%) of them were grand multiparas. The majority, 411(83%) of women delivered in a health facility, with 275(55.6%) of them gave birth through cesarean section. Regarding labor outcomes and duration, 342 (69.1%) women experienced stillbirths, and 261 (52.7%) experienced prolonged labor (Table 2).

**Table 2:** Obstetric characteristics of obstetric fistula patients admitted at Hamlin fistula center Addis Ababa Ethiopia 2023 (N = 495).

Variables	Category	Frequency	Percent
Parity	Primiparous (1)	149	30.1
	Multipara (2–4)	228	46.1
	Grand multipara (≥5)	118	23.8
Place of delivery	Health facility	411	83.0
	Home delivery	84	17.0
Duration of labor for the index child in days	Not prolonged (≤12hr)	234	47.3
	Prolonged (>12hr)	261	52.7
Mode of delivery for the index child	Assisted vaginal delivery	37	7.5
	Spontaneous vaginal delivery	183	37.0
	cesarean section	275	55.6
Outcome of delivery	Alive	153	30.9
	Stillbirths	342	69.1

### Fistula related characteristics

Most of the fistula cases 303(61.2%) were developed in the age range of 19–30 years and 461(93.1%) of them received prophylactic antibiotics. All OBF patients had at least one form of incontinence, of which 440(88.9%) of them had urinary incontinence, while only one patient underwent catheterization before surgery.

Regarding surgical history, data from 85 patients (17.2%) indicated they had undergone previous surgery due to a fistula, with only 6 (1.2%) having undergone three surgical procedures. Concerning type of fistula, most of them 431(87.1%) were Vesico-Vaginal Fistula (VVF), followed by 127(25.7%) mixed cases, and 52(10.5%) of them were from iatrogenic. Furthermore, 50(10.1%) of patients had complete urethral distraction, while 87(17.6%) and 16(3.2%) of them had severe vaginal scarring and severe fibrosis respectively. A vaginal surgical approach was performed in 441(89.1%) patients. The results also showed that 370(74.7%) of the OBF patients recovered, while the remaining 125(25.3%) were censored (Table 3).

**Table 3:** Fistula related characteristics patients admitted at Hamlin fistula center Addis Ababa Ethiopia 2023 (N = 495).

Variables	Category	Frequency	Percent
Age at development of fistula in years	≤18 age	57	11.5
	19-30 age	303	61.2
	≥31 age	135	27.3
Took prophylaxis’s antibiotic	Yes	461	93.1
	No	34	6.9
Type of incontinency	Fecal	26	5.3
	Urinary	440	88.9
	Urinary Fecal	29	5.8
Duration of urinary incontinence in days	Short duration (<30 days)	44	8.9
	Medium duration (30–365 days)	370	74.7
	Long duration (>365 days)	81	16.4
Got physiotherapy before surgery	Yes	12	2.4
	No	483	97.6
Got physiotherapy after surgery	Yes	36	7.3
	No	459	92.7
Got psychotherapy before surgery	Yes	200	40.4
	No	295	59.6
Got psychotherapy after surgery	Yes	93	18.8
	No	402	81.2
Got any surgery for the fistula previously at any fistula center	Yes	85	17.2
	No	410	82.8
Frequency of previous surgery (n = 85)	once	62	72.9
	Twice	17	20.0
	Three times	6	7.1
**Type and characteristics of fistula**			
Vesico-vaginal fistula (VVF) (n = 495)	No	64	12.9
	Yes	431	87.1
Length of vesico-vaginal fistula (n = 431)	2.2-4 cm	355	82.4
	≥5 cm	76	17.6
VVF size based on distance from bladder (n = 431)	<5 cm	21	4.9
	>5 cm	410	95.1
Width of the VVF hole (n = 431)	0-2 cm	272	63.1
	2.2-4 cm	103	23.9
	≥5 cm	56	13.0
Rectovaginal fistula (RVF) (n = 495)	No	443	89.5
	Yes	52	10.5
Distance from hymen for RVF (n = 495)	No RVF types	443	89.5
	0-2 cm	32	6.5
	3-5 cm	16	3.2
	>5 cm	4	0.8
Iatrogenic fistula (n = 495)	No	443	89.5
	Yes	52	10.5
Ureteric fistula (n = 495)	No	457	92.3
	Yes	38	7.7
Mixed fistula (n = 495)	No	368	74.3
	Yes	127	25.7
Status of urethra (n = 495)	Partially damaged	166	33.5
	Not damaged	279	56.4
	Complete urethral distraction	50	10.1
Stage of vaginal scaring (n = 495)	No vaginal scaring	98	19.8
	Mild	142	28.7
	Moderate	168	33.9
	Severe	87	17.6
Fistula fibrosis(recto) (n = 495)	No	443	89.5
	Mild	18	3.6
	Moderate	18	3.6
	Severe	16	3.2
Surgery Approach (n = 495)	Vaginal	441	89.1
	Both	17	3.4
	Abdominal	37	7.5
Duration of urethral catheterization after procedure in days (n = 495)	No	22	4.4
	1-5	333	67.3
	6-10	137	27.7
	>11	3	.6
Duration of day before treatment (n = 495)	≤1 year	348	70.3
	1-5 years	76	15.4
	>5 years	71	14.3
Outcomes (n = 495)	Censored	125	25.3
	Recovered	370	74.7

#### Time to recovery from obstetric fistula.

The patients with OBF were followed up for a total of 5932 person-weeks at risk and under observation. The overall person time recovery rate from OBF was 6.34 per 100 person-weeks (95% CI: 5.7, 7.0).

**Fig 1:**
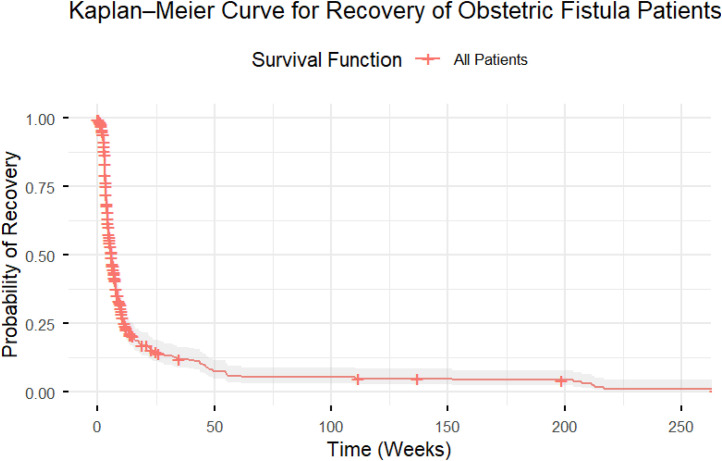
Kaplan-Meier survival curve for estimating the recovery time of obstetric fistula patients at Hamlin fistula center Addis Ababa Ethiopia from January 2017 to June 2023 (n = 495).

Kaplan-Meir curve showed that recovery time estimates revealed that among the included patients, 74.7%, (95%CI: 71.1, 78.2) were recovered from OBF. Among the 125 censored patients, 108 (86.4%) had incomplete recovery, 15 (12.0%) were lost to follow‑up, and 2 (1.6%) were transferred to other facilities, with no recorded deaths. The median recovery time was 5.9 weeks, with an interquartile range (IQR) of 3.57-11.29 weeks (Fig 1).

Significant differences in median recovery time were observed among those with different occupational statuses. The median recovery time among those who were engaged in farming activity was 6.9 weeks, whereas those engaged in as government employee had 5 weeks median recovery time (p-value = 0.45). Furthermore, the log-rank test revealed significant differences in recovery times based on the type of incontinence. The median recovery time was longer (7.4 weeks) if the women had mixed incontinence, while it was relatively shorter for those with fecal incontinence (3.3 weeks) (p value = 0.002).

**Fig 2:**
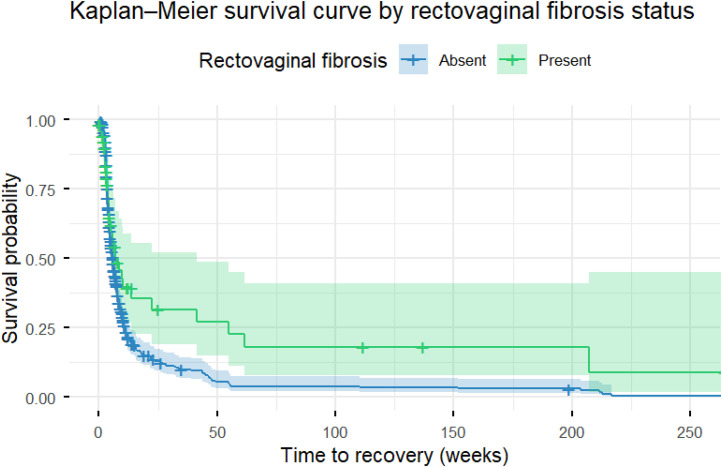
Kaplan-Meier survival curve for recto vaginal fibrosis on the recovery time among obstetric fistula patients at Hamlin fistula center Addis Ababa Ethiopia from January 2017 to June 2023 (n = 495).

According to the log-rank test, a significant difference was observed in the recovery time between patients with and without rectovaginal fistula. Patients with rectovaginal fistula needed more time (7.1 weeks) to recover than those women don’t have rectovaginal fistula (5.7 weeks) (p-value of 0.014). Similarly, the median recovery time varied significantly according to the severity of rectovaginal fibrosis. Patients with severe rectovaginal fibrosis required much longer (22.4 weeks) to recover than those with mild fibrosis (4 weeks) (p-value = 0.011) (Fig 2).

The median recovery time varied significantly depending on the surgical approach. Patients who underwent both vaginal and abdominal surgery required more time to recover (10 weeks) than those who underwent only vaginal (5.9 weeks) or only abdominal (5.4 weeks) surgery (p-value = 0.038). The recovery time also differed significantly depending on the urethral status. Patients with complete urethral damage required 8 weeks to recover (p value = 0.05).

**Fig 3:**
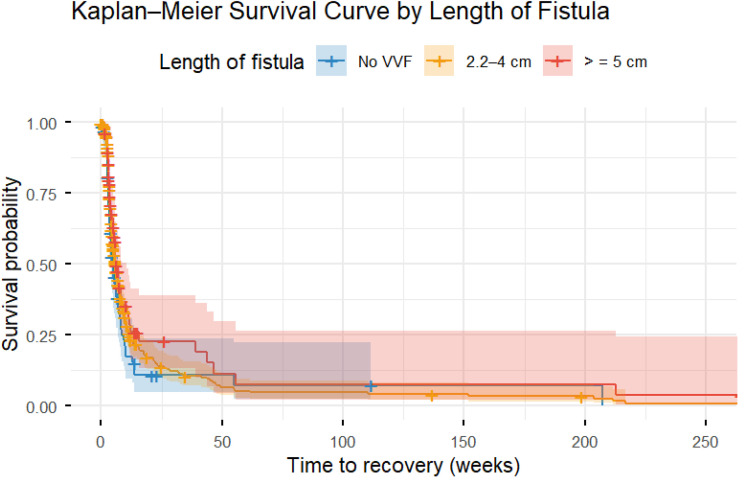
Kaplan-Meier survival curve for the length of fistula on the recovery time among obstetric fistula patients at Hamlin fistula center Addis Ababa Ethiopia from January 2017 to June 2023 (n = 495).

Patients with severe vaginal scarring had a median recovery time of 9.7 weeks, while those without scarring recovered in 5.3 weeks (p-value = 0.000). The location of the fistula also affected the recovery time. Patients with Recto-Vaginal Fistula (RVF) whose fistula was close to the hymen required 8.7 weeks to recover, while those whose fistula, was far from the hymen recovered in 3.1 weeks (p-value = 0.010). Moreover, the distance of the fistula from bladder influenced the recovery time. Patients with a fistula of less than 5 cm from bladder took 8.7 weeks to recover, while those with a larger distance from bladder recovered faster (p-value = 0.042) (Table 4) (Fig 3).

**Table 4:** Comparisons of time to recovery among different levels of predictor variables using log-rank test from obstetric fistula patients admitted at Hamlin fistula center Addis Ababa Ethiopia 2023 (N = 495).

Variables	Category	Frequency (%)	Median recovery time (weeks)	Log rank test	
				X2	P Value
Occupation	Farmer	249(50.3)	6.9	6.2	.045
	Housewife	173(34.9)	5.6		
	governmental employee and others	73(14.7)	5.0		
Type of incontinence	Urinary	440(88.9)	6.0	12.0	.002
	Fecal	26(5.3)	3.3		
	Both	29(5.8)	7.4		
Fecal incontinence	No	441(89.1)	5.7	7.0	.008
	Yes	54(10.9)	8.7		
Recto vaginal fistula	No	443(89.5)	5.7	6.0	.014
	Yes	52(10.5)	7.1		
Recto vaginal fibrosis (n= 52)	Mild	18(34.6)	4.0	11.1	.011
	Moderate	18(34.6)	10.1		
	Severe	16(30.8)	22.4		
Surgery approach	Vaginal	441(89.1)	5.9	6.5	.038
	Abdominal	37(7.5)	5.4		
	Both	17(3.4)	10.0		
Status of urethra	Not damaged	279(56.4)	5.4	6.0	.050
	Partially damaged	166(33.5)	6.0		
	Completely damaged	50(10.1)	8.0		
Stage of vaginal scarring	No vaginal scaring	98(19.8)	5.3	21.9	.000
	Mild	142(28.7)	5.6		
	Moderate	168(33.9)	5.1		
	Severe	87(17.6)	9.7		
Distance from hymen RFV (n = 52)	0-2 cm	32(61.5)	8.7	11.3	.010
	3-5 cm	16(30.8)	5.9		
	>5 cm	4(7.7)	3.1		

### Cox Proportional Hazards (PH) Model adequacy assessment

The adequacy of the Cox proportional hazard model was evaluated using a log-minus-log plot for each variable prior to entering into the final model. For each covariate incorporated into the model, a plot was used to estimate the recovery period for patients with OBF. The log minus log plot assumption was satisfied for that covariate if the plot demonstrated random parallel or not cross each other’s. The proportional hazards assumption was verified using log-minus-log plots and Schoenfeld residuals.

On plot checking: Body Mass Index (BMI), place of delivery, outcome of delivery, age at development of fistula in years, got physiotherapy after surgery, got psychotherapy before surgery, got psychotherapy after surgery, length and width of fistula, rectovaginal fistula and status of urethra were predictors that satisfied the PH assumption. Moreover, marital status, occupational status, educational status, distance from the hymen, stage of vaginal scarring, fistula fibrosis and surgical approach were satisfied after adjustments were made. However, getting ureteral catheterization before surgery, vesico-vaginal fistula and bladder size were removed due to violation of the assumption even after adjustments were made.

#### Checking model fitness by using Cox-Snell residual plots.

**Fig 4:**
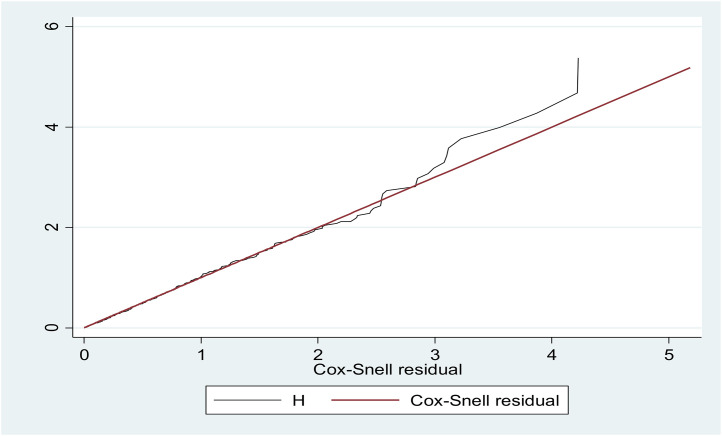
The Cox-Snell residual plots for log-logistic, distributions of time to recovery from obstetric fistula patients at Hamlin fistula center Addis Ababa Ethiopia from January 2017 to June 2023.

Cox-Snell residuals were used to assess the goodness of fit of the model. The log logistic Cox-Snell residual plot appeared to align closely with a 45-degree line passing through the reference line. This shows that the observed line closely aligns with the reference line across the majority of the distribution, with only minor deviations at higher residual values. Therefore, the log-logistic model adequately fitted the data, indicating that the model assumptions were reasonably met (Fig 4).

The final Cox model showed a better fit than the null model (Omnibus: χ² = 55.12, df = 25, p < 0.001). Cox-Snell residuals confirmed an adequate fit (Fig 4), with observed values following the 45° reference line. Minor deviations at residuals >4 reflect fewer long-term observations but do not invalidate the model.

#### Predictors of recovery time from obstetric fistula.

Cox regression analysis was employed in a survival analysis to determine the predictors of OBF recovery time. Twenty-two independent variables were examined using the Cox proportional hazards model with recovery time as the dependent variable.

In the bivariate Cox proportional hazard analysis, variables with a p-value of 0.25 or less were included in the multivariate Cox proportional hazard regression analysis. Among these, only five variables showed statistical significance concerning recovery time, with a p-value of 0.05 or lower, along with their respective Adjusted Hazard Ratios (AHR) and 95% confidence intervals (CI).

The results showed that patients with complete urethral distraction were 57% less likely to recover faster than those with no urethral distraction (AHR: 0.43, 95% CI: 0.25, 0.76). Patients who received physiotherapy after surgery were 1.85 times more likely to recover faster than those who did not receive physiotherapy (AHR: 1.85, 95% CI: 1.13, 3.02). Patients with a small fistula length (VVF) (2.2 cm to 4 cm) were 5.23 times more likely to recover faster than those with a large fistula length (more than or equal to 5 cm) (AHR: 5.23, 95% CI: 1.17, 12.33).

VVF patients with severe vaginal scarring were 40% less likely to recover faster than those with mild or no vaginal scarring (AHR: 0.60, 95% CI: 0.40, 0.89). RVF patients with mild fistula fibrosis were 39% less likely to recover faster than those with no fistula fibrosis (AHR: 0.61, 95% CI: 0.40, 0.84) (Table 5).

**Table 5:** Bivariate and multivariable cox proportional hazard ratio indicating predictors of recovery time from obstetric fistula patients admitted at Hamlin fistula center Addis Ababa Ethiopia 2023 (N = 495).

Variable	Category	Survival status		CHR 95% CI	P value	AHR 95% CI	P value
		Recovered	Censored				
Marital status	Married	292	85	**1**		**1**	
	Others**	78	40	0.81(0.63,1.04)	0.096	.98(0.74,1.29)	0.866
Educational status	No formal education	226	84	**1**		**1**	
	Had formal education	144	41	1.06(0.86,1.31)	0.574	0.92(0.72,1.17)	0.490
Occupation of the women	Farmers	176	73	**1**		**1**	
	Others***	194	52	1.15(0.94,1.41)	0.185	1.20(0.96,1.50)	0.103
BMI	Normal weight	233	90	**1**		**1**	
	Abnormal weight	137	35	1.01(0.89,1.24)	0.997	0.98(0.78,1.22)	0.835
Place of Delivery	Health facility	316	95	1		1	
	Home delivery	54	30	0.77(0.57,1.03)	0.074	.90(0.65,1.24)	0.513
Outcome of delivery	Alive	133	20	1		1	
	Stillbirths	237	105	0.87(0.70,1.08)	0.198	1.09(0.89,1.39)	0.514
Age at development of fistula in years	<=18 age	35	22	1		1	
	19-30 age	222	81	1.11(0.78,1.59)	0.569	.90(0.62,1.32)	0.605
	>=31 age	113	22	1.41(0.96,2.06)	0.077	1.09(0.72,1.65)	0.680
Type of incontinency	Urinary	333	107	1		1	
	Both urinary and fecal	37	18	1.28(0.91,1.80)	0.156	1.23(0.86,1.75)	0.260
Got physiotherapy after surgery	Yes	21	15	1.78(1.14,2.79)	0.011	1.85(1.13,3.02)*	**0.015**
	No	349	110	1		1	
Got psychotherapy before surgery	Yes	142	58	1		1	
	No	228	67	1.17(0.95,1.45)	0.144	0.99(0.78,1.24)	0.901
Got psychotherapy after surgery	No	62	31	1		1	
	Yes	308	94	1.32(1.00,1.75)	0.05	1.15(0.86,1.55)	0.344
Length of fistula of VVF	No VVF	53	11	1.44(0.98, 2.12)	0.06	1.18(0.67, 2.06)	0.09
	2.2-4 cm	266	89	1.17(0.87, 1.58)	0.29	5.23(1.17,12.33)*	**0.03**
	>=5 cm	51	25	1		1	
Width of fistula hole	No VVF	51	11	1.40(0.93, 2.14)	0.12	0.99(0.53, 1.86)	0.994
	0-2 cm	211	63	1.22(0.86, 1.72)	0.24	0.512(0.12,2.29)	0.384
	2.2-4 cm	69	34	1.09(0.74, 1.62)	0.65	1.01(0.53,1.92)	0.908
	>=5 cm	39	17	1		1	
Rectovaginal fistula	No	339	104	1		1	
	Yes	31	21	0.63(0.44,0.92	0.016	0.66(0.17,2.63)	0.560
Distance from hymen	No RVF	335	104	1.43(0.85, 2.39)	0.181	1.15(0.31, 4.23)	0.83
	0-2 cm	20	12	0.76(0.38, 1.51)	0.76	0.56(0.27, 1.16)	0.12
	≥3 cm	15	9	1		1	
Status of urethra	Not damaged	226	53	1		1	
	Partially damaged	117	49	0.93(0.74,1.16)	0.527	0.99(0.78,1.28)	0.994
	Complete urethral distraction	27	23	0.61(0.41,0.91)	0.016	0.43(0.25,0.76)*	**0.004**
Stage of vaginal scarring	Mild(no) vaginal scaring	210	30	1		1	
	Moderate vaginal scarring	122	46	0.91(0.72,1.13)	0.390	0.98(0.76,1.27)	0.882
	Severe vaginal scarring	38	49	0.45(0.32,0.64)	0.000	0.60(0.40,0.89)*	**0.012**
Fistula fibrosis(recto)	RVF not present	339	104	1		1	
	RFV present	31	21	0.63(0.44,0.92)	0.016	0.61(0.40,0.83)*	**0.02**
Surgery Approach	Vaginal	331	110	1		1	
	Abdominal or both	39	15	1.03(0.74,1.44)	0.854	0.97(0.52,1.80)	0.920

Keys: *P-value ≤ 0.05, **separated, divorced, widowed and single, ***Housewife, Self-employed, government-employee, and student.

## Discussions

The study found that the median time of recovery for fistula patients was 5.9 weeks (95%CI = 5.1,6.6) among OBF patients in Hamlin fistula center Addis Ababa. The time to recovery from OBF was significantly affected by predictors such as physiotherapy after surgery, VVF length, urethral status, vaginal scarring stage, and rectal fistula fibrosis.

The current findings indicated that OBF was healed in 74.7% (95%CI: 71.1, 78.2) of the patients, with a median duration of 5.9 weeks. The recovery rate was consistent with the study conducted at Gondar teaching hospital fistula center (73.7%) [20]. However, our finding 74.7% is lower than studies conducted in Gondar (88.07%), Jimma (81.4%), and Mekelle (89.33%) [21,22,28] (S1 Table). This reflects Hamlin Fistula Hospital’s role as a referral center. Our cohort included: 17.2% with prior failed surgery, 10.1% complete urethral destruction, 16.4% with fistula greater than 1 year. The median recovery time (5.9 weeks) matches that of other centers (5.14 to 5.19 weeks), suggesting comparable surgical quality. A lower overall rate reflects case-mix, not inferior care. Overall, although Hamlin Fistula Hospital in Addis Ababa, which is situated in the capital city, receives complex and chronic cases referred from across the country, often involving extensive tissue damage or coexisting complications. These factors may contribute to the relatively lower recovery rate observed compared to other centers that serve less complicated cases.

The median recovery time for patients with fistula in this study was 5.9 weeks, which was in line with the studies conducted at Gondar University Teaching and Referral hospital, which reported an average (median) time of recovery of 5.14 weeks [22], and Gondar teaching hospital fistula center, which reported a median recovery time of 5.19 weeks [20]. However, this study had a longer recovery time than the study conducted at the Harar Hamlin Fistula Center, which reported 18.71 days (2.67 weeks) [23] (S1 Table). The variation in recovery time and the use of mean and median recovery times may be attributed to differences in the settings, particularly as the current facility serves as a referral center where patients present with diverse complications. Additionally, Hamlin Fistula Hospital Addis Ababa serves as a referral center for most patients with failed previous repair and the health-seeking behavior of caregivers or parents may influence the recovery duration.

This study found that complete urethral damage, patients with VVF and severe vaginal scarring affected the recovery time from OBF. Patients with complete urethral damage had a 57% lower chance of recovering faster than those without urethral damage. This result is in line with a study conducted at Harar Hamlin Fistula Center [23,28]. This might be because repairing urethral fistula is a complicated process that requires restoring the surviving tissues as a flexible and functional organ and controlling the urine passage at proper times. A nonfunctional and scarred urethra results in incontinence and longer recovery time. Furthermore, patients with VVF and severe vaginal scarring had a 40% lower likelihood of faster recovery from OBF than those without vaginal scarring. This result was similar to that of a study conducted at Harar Hamlin Fistula Center [23]. This might be because scarred tissue has a weak blood supply and heals slowly. Scarred fistulas are also difficult to separate from the adjacent structures, such as the vagina and pubis, making repair without tension difficult. Moreover, scarred tissue can block access to the fistula site and allow the use of unhealthy tissue to seal the hole. Scarring of the vagina could also keep the urethra open, impairing its healing process and normal function [34,35].

Fistula fibrosis affects the time to recovery from OBF. RVF patients with mild fistula fibrosis had a 39% lower chance of recovering faster than those without fistula fibrosis. This might be because fibrosis is a tissue scarring that occurs as a pathological deviation from the normal wound healing response. Scarring results from increased and prolonged inflammation, leading to slow wound healing. Overall, these findings underscore that patients with complete urethral damage, severe vaginal scarring and fistula fibrosis may require specialized and individualized care.

The length of the VVF affected the recovery time of patients with VVF. Patients with fistula length 2.2-4 cm had a 5.23 times higher chance of recovering faster than those with fistula length greater than or equal to 5 cm. This suggests that the recovery time increased as the fistula length increased. This result is consistent with a study conducted in Gondar, Northwest Ethiopia [22]. The wide confidence interval for length of VVF (AHR: 5.23, 95% CI: 1.17–12.33) may have limited the precision of the estimated effect, and the result should be interpreted with caution.

Furthermore, physiotherapy after surgery increased the recovery rate of patients with OBF by 1.85 times compared to those who did not receive physiotherapy. This is consistent with a study conducted at Mekele Hamlin Fistula Center, Ethiopia [21]. Physiotherapy involves various activities such as physical exercise and electronic stimulation. This enhances blood flow and nourishes damaged tissue, making the healing process faster than usual. In this study, only 7.3% of OBF patients received physiotherapy despite 85% faster recovery. This suggests that physiotherapy is an important clinical intervention that could substantially improve recovery outcomes if integrated into routine postoperative OBF patient care.

The findings of the current study underscore the significant clinical implications for enhancing individualized rehabilitation services for patients with OBF, aiming to improve their recovery time and quality of life. Incorporating physiotherapy into the postoperative care of patients with OBF could be a feasible strategy to improve patient recovery. Moreover, offering a specialized care for women with urethral injuries or severe vaginal scarring can shorten the recovery time. Hospital administrators, health facilities, and healthcare providers must work to address the barriers to receiving quality postoperative cares, such as physiotherapy services, strengthen rehabilitation programs, and adopt individualized women centered care for patients with OBF.

The strengths of the current study include the inclusion of data from randomly selected patients treated over seven years at a specialized fistula center and the use of survival analysis for time to recovery and its predictors, strengthening the reliability of findings. However, this study has several limitations. The study was based on a retrospective design using secondary data, which limits the accuracy and completeness of the information from the records. There is a potential bias from incomplete documentation, data quality or inconsistent clinical recording practices over time. Furthermore, potential predictors for OBF such as household wealth, sociocultural factors, nutritional status, distance from health facilities, antenatal care follow-up, psychosocial supports and providers skill and other related factors were not collected in this study. The absence of these factors on the patients’ charts may have introduced unmeasured confounding, limiting the ability to fully adjust for potential determinants influencing the time to recovery time from OBF. Additionally, since the study included only patients who received surgical treatment and had complete follow-up data, selection bias is possible, as women without care or with incomplete records were excluded. As the current study was conducted in one tertiary center, the findings may not reflect the situation of primary-level repairs. The current study used physical repair and continence only to consider patients with OBF as recovered and did not consider the World Health Organization’s multidimensional outcomes of health such as psychological wellbeing, social reintegration and functional or economic restorations [36]. These may affect the generalizability of findings to all patients with OBF in Ethiopia.

## Conclusions

Based on the findings of this study, recovery rate of the entire cohort and median time of recovery were within the sphere of standard (74.7% recovery rate with median recovery time of 5.9 weeks). Physiotherapy after surgery, VVF length, vaginal scarring, urethral status and RVF fibrosis were predictors of OBF recovery time. Therefore, the Fistula Center and health care providers must scale up physiotherapy services for all OBF patients, as only 7.3% received physiotherapy despite 85% faster recovery (AHR = 1.85) among those who received it. To improve the recovery rate and time from OBF for all patients from the current level (74.7%). Counselling and organizing support for complex cases such as women with urethral destruction who have slower recovery (57%) compared to others. Identification of patients with severe vaginal scarring (17.6%) at admission for prioritized physiotherapy, nutritional support to improve extended hospitalization and to facilitate faster recovery. Future research should prioritize the utilization of prospectively collected data and a wider range of health facilities treating OBF to obtain more comprehensive information, including individual, health service and psycho-social and other related factors not addressed in this study.

## Supporting information

S1 Fig Flow Chart for participant selection. (DOCX)

S1 Data SPSS data set. (SAV)

S1 Table Comparison of findings with other Ethiopian studies. (DOCX)

## Abbreviations:

AHR: Adjusted Hazard Ratios
BMI: Body Mass Index
OBF: Obstetric Fistula
RVF: Recto-Vaginal Fistula
VVF: Vesico-Vaginal Fistula.
