# Supplementary material for: Time to recovery from Obstetric Fistula and its predictors among patients admitted to Hamlin Fistula Center, Addis Ababa, Ethiopia: A survival analysis
Source: PLOS Glob Public Health. 2026 Jul 9;6(7):e0006848. doi: 10.1371/journal.pgph.0006848 (PMC13349304; doi:10.1371/journal.pgph.0006848)
Supplement: S1 Fig — (DOCX) [file pgph.0006848.s001.docx]

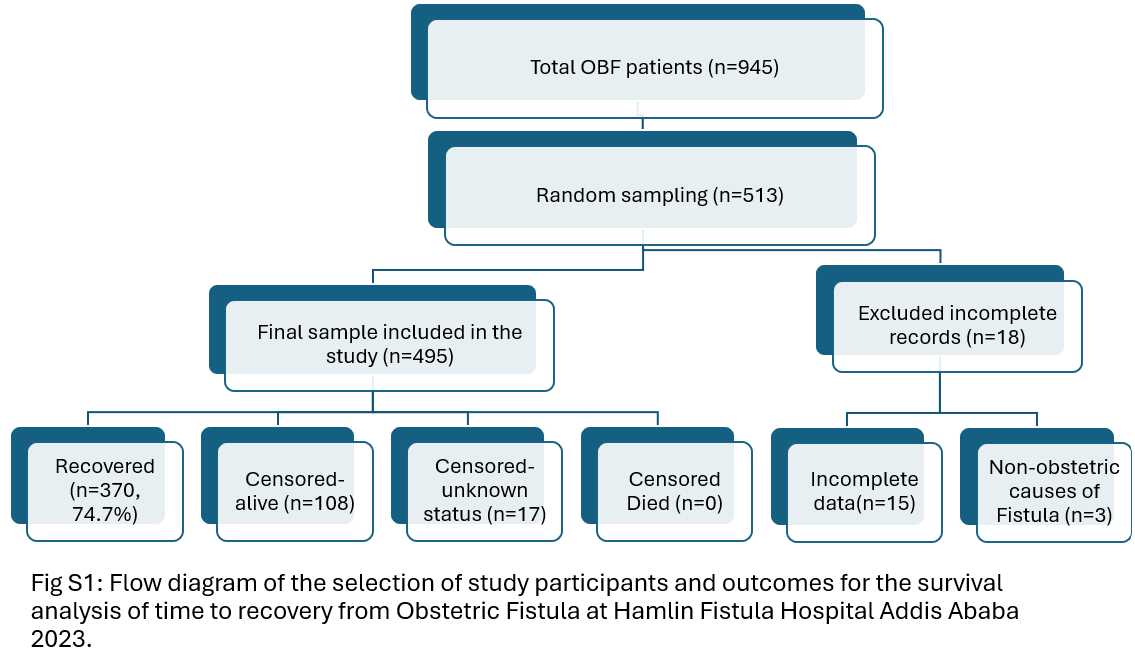


S1 Fig. Flow diagram of the selection of study participants and outcomes for the survival analysis of time recovery from Obstetric Fistula at Hamlin Fistula Hospital Addis Ababa 2023.
