# Supplementary material for: Time to recovery from Obstetric Fistula and its predictors among patients admitted to Hamlin Fistula Center, Addis Ababa, Ethiopia: A survival analysis
Source: PLOS Glob Public Health. 2026 Jul 9;6(7):e0006848. doi: 10.1371/journal.pgph.0006848 (PMC13349304; doi:10.1371/journal.pgph.0006848)
Supplement: S1 Table — (DOCX) [file pgph.0006848.s003.docx]

S1 Table. Table comparing findings with other Ethiopian studies

| **Study** | **Location** | **Sample** | **Recovery Rate** | **Median Time** | **Notes** |
| --- | --- | --- | --- | --- | --- |
| Current study findings | Hamlin | 495 | 74.7% | 5.9 weeks | Tertiary referral |
| Derso EA et al., 2020 | Gondar | 289 | 73.7% | 5.19 weeks |  |
| Yismaw L et al., | Gondar University | 612 | 88.07% | 5.14 weeks |  |
| Hussen S et al., 2017 | Harar | 433 | 67.21% | 2.67 weeks | Mean, not median |
| Bihon Am et al., 2022 | Mekelle | 228 | 89.33% | 4.7 weeks | Mean 6 weeks |
| Areba et al., 2022 | Jimma University | 270 | 81.4% | --- | Not reported |
