# Supplementary material for: Time to recovery from Obstetric Fistula and its predictors among patients admitted to Hamlin Fistula Center, Addis Ababa, Ethiopia: A survival analysis
Source: PLOS Glob Public Health. 2026 Jul 9;6(7):e0006848. doi: 10.1371/journal.pgph.0006848 (PMC13349304; doi:10.1371/journal.pgph.0006848)
Supplement: Attachment — Submitted filename: PGPH-D-25-02015_R1 (1).pdf [file pgph.0006848.s007.pdf]

PLOS Global Public Health

Time to recovery from Obstetric Fistula and its Predictors among Obstetric Fistula patients admitted to Hamlin Fistula Center, Addis Ababa, Ethiopia: A Survival Analysis.

--Manuscript Draft--

**Manuscript Number:**	PGPH-D-25-02015R1
**Article Type:**	Research Article
**Full Title:**	Time to recovery from Obstetric Fistula and its Predictors among Obstetric Fistula patients admitted to Hamlin Fistula Center, Addis Ababa, Ethiopia: A Survival Analysis.
**Short Title:**	Time to recovery from Obstetric Fistula
**Corresponding Author:**	Gizachew Abdissa Bulto<br>Ambo University<br>Ambo, Oromia ETHIOPIA
**Order of Authors:**	Hayat Ibrahim Oumer
	Delelegn Yilma Gebremichael
	Gizachew Abdissa Bulto
**Keywords:**	Time to recovery, Obstetric Fistula, Predictors, Ethiopia
**Abstract:**	The incidence of obstetric fistula (OBF) remains high, posing significant psychological, physical, and health challenges, yet only a limited number of health facilities in Ethiopia offer treatment services. Moreover, there is also a paucity of evidences on the time to recovery from OBF and its predictors at treatment centers. Therefore, it was aimed to identify the time to recovery from obstetric fistula and predictors among obstetric fistula patients admitted at Hamlin fistula hospital Addis Ababa, Ethiopia.<br>A Hospital based retrospective cohort study was employed among 513 obstetric fistula cases admitted at Hamlin fistula hospital, 2023. A simple random sampling technique was employed to recruit 513 patient records. Data were extracted from the patients' medical charts and collected by using kobo tool box software. The Kaplan-Meier method was employed to estimate the distribution of recovery time, and the log-rank test was utilized to assess the experience of recovery time among different levels of categorical variables. Bivariate and multivariable Cox proportional regression analyses were conducted, with statistical significance determined at a p-value < 0.05 and adjusted hazard ratio (AHR).<br>The overall recovery rate of the entire cohort was 74.7%, (95%CI: 71.1, 78.2) and median time to recovery from obstetric fistula was 5.9 weeks (95%CI=5.1, 6.6). Getting physiotherapy after surgery (AHR:1.13,95%CI:1.13, 3.02), urethral status (damaged) (AHR: 0.43,95CI: 0.25,0.76), length of fistula (2.2.-4cm) (AHR:5.23, 95%CI: 1.17, 12.33), and having severe vaginal scarring (AHR: 0.60, 95%CI: 0.40, 0.89) were predictors of time to recovery from OBF.<br>Based on the findings, median recovery time from OBF was 5.9 weeks. Getting physiotherapy after surgery, urethral status, having moderate and severe vaginal scarring were predictors that affect its time of recovery. Stakeholders such as Hamlin fistula centers and health workers should give due attention on the identified predictors for smooth and earlier time of recovery from OBF.
**Additional Information:**	
**Question**	**Response**
**Financial Disclosure**<br><br>Enter a financial disclosure statement that describes the sources of funding for the work included in this submission and the role the funder(s) played. This includes	The authors received no specific funding for this work.

Powered by Editorial Manager® and ProduXion Manager® from Aries Systems Corporation

grants and any commercial funding of the work or authors.<br><br>This statement will be typeset if the manuscript is accepted for publication.<br><br>*Please review the submission guidelines and the instructions link below for detailed requirements and guidance.*	
**Competing Interests**<br><br>On behalf of all authors, disclose any competing interests that could be perceived to bias this work.<br><br>This statement will be typeset if the manuscript is accepted for publication.<br><br>*Please review the instructions link below and PLOS Global Public Health's competing interests policy to determine what information must be disclosed at submission.*	The authors have declared that no competing interests exist.
**Data Availability**<br><br>Before publication, Authors are required to make fully available and without restriction all data underlying their findings. Please see our PLOS Data Policy page for detailed information on this policy.<br><br>A **Data Availability Statement**, detailing where the data can be accessed, is required at first submission. Insert your Data Availability Statement in the box below.<br><br>Please see the data reporting section of our submission guidelines for instructions on what you need to include in your Data Availability Statement.	The datasets used and/or analyzed during the current study are available as supporting information with this paper.

This statement will be typeset if the manuscript is accepted for publication. *PLOS allows rare exemptions to address legal and ethical concerns. If you have legal or ethical restrictions, please use the box below to detail these in full sentences for the Journal team to consider.*	

Manuscript Click here to access/download;Manuscript;V1 Manuscript OBF 14, 2025.docx

Time to recovery from Obstetric Fistula and its Predictors among Obstetric Fistula patients admitted to Hamlin Fistula Center, Addis Ababa, Ethiopia: A Survival Analysis.

Hayat Ibrahim Oumer[1], Delelegn Yilma Gebremichael[2], Gizachew Abdissa Bulto[3]*

[1]Addis Ababa City Health Bureau, Addis Ababa, Ethiopia.
[2]Ambo University, College of Health Sciences and Referral Hospital, Department of Public Health, Ambo, Ethiopia.
[3]Ambo University, College of Health Sciences and Referral Hospital, Department of Midwifery, Ambo, Ethiopia.

*Corresponding author

Address:
HIO: ihayat806@gmail.com
DYG: delelegnyilma@gmail.com
GAB: gizachab@yahoo.com

Abstract

Obstetric Fistula (OBF) remains a devastating maternal condition in Ethiopia, causing significant physical suffering, social isolation and psychological traumas among affected women. Despite its burden, only a limited number of healthcare facilities in Ethiopia provide surgical interventions. Furthermore, there is a scarcity of evidence regarding the time to recovery from OBF and its predictors at treatment centers. Therefore, this study aimed to determine the time to recovery from OBF and its predictors among patients admitted to Hamlin Fistula Hospital Addis Ababa, Ethiopia. A Hospital based retrospective cohort study was employed among 513 women treated for OBF at Hamlin fistula hospital from January 1st, 2017, to June 30th, 2023. A simple random sampling technique was employed to recruit 513 patient records and data were collected by using kobo toolbox software. The Kaplan-Meier method was employed to estimate the distribution of recovery time, and the log-rank test was utilized to assess the experience of recovery time among different levels of categorical variables. Bivariate and multivariable Cox proportional regression analyses were conducted, with statistical significance determined at a p-value < 0.05 and adjusted hazard ratio (AHR).

The overall recovery rate was 74.7%, (95%CI: 71.1, 78.2), with a median recovery time from OBF was 5.9 weeks (95%CI: 5.1, 6.6). Receiving physiotherapy after surgery (AHR: 1.13, 95% CI: 1.13–3.02) and having a fistula length of 2.2–4 cm (AHR: 5.23, 95% CI: 1.17–12.33) were found to be predictors of faster recovery for OBF. Conversely, women with urethral damage (AHR: 0.43, 95% CI: 0.25–0.76) or severe vaginal scarring (AHR: 0.60, 95% CI: 0.40–0.89) experienced delayed recovery from OBF.

Based on the findings, median recovery time from OBF was 5.9 weeks. Therefore, strengthening postoperative physiotherapy and providing specialized care for women with urethral injury or severe vaginal scarring could improve recovery time from OBF. These findings also highlighted

the critical importance of individualized rehabilitation services in fistula treatment centers to optimize patient recovery and overall quality of life.

Background

Obstetric Fistula (OBF) "is an abnormal opening or a hole between the vagina and urinary bladder or/and rectum or both that is resulted from prolonged and obstructed labor, surgical error, or trauma" (1)(2). Untreated, OBF leads to severe, lifetime morbidities with profound medical, psychological traumas and socio-economic consequences (3). It is one of the most neglected human rights and public health problems, causing tremendous challenges such as emotional distress, social isolation, family abandonment, and stigma. As reported by Animut et al., women with OBF experience profound social isolation, divorce, health problems, and psychological trauma. One woman expressed, "I am a person who is standing but not alive. […] However, I understand one thing darkness surrounds us despite the sun keeps rising" (crying). The majority (91%) of affected women experience stillbirths, while many also face anxiety, depression, discrimination, and worsening poverty. Common physical complications include nerve damage, foot drop, urinary and fecal incontinence, infertility, renal failure, and other pelvic disorders (4–8)(9).

OBF occurs mainly in sub-Saharan Africa and Asia (10–12). Without timely access to emergency caesarean section, women with obstructed labor face higher risks of developing OBF (3,13). Each year, 50,000-100,000 women worldwide develop OBF. In Sub-Saharan Africa and Southeast Asia, up to 3.5 million women may live with untreated OBF, potentially more due to unreported cases (10). In Ethiopia, an estimated 72,533 women of reproductive age have experienced fistula. Of these, 31,961 cases remain untreated, whilst 40,572 have received treatment. Annually, 948 new cases of OBF are reported, constituting approximately 1.6% of total obstructed and prolonged

labor cases and 0.03% of total deliveries in 2020. The incidence of untreated vaginal fistula (VF) symptoms was also estimated to be 1.4 cases per 1000 in Ethiopia (14)(15). Although the government of Ethiopia in its strategic plan aimed to eliminate OBF by 2025, it remained a significant contributor to maternal morbidities. The point prevalence of OBF is 1.4 per 1000 women of childbearing age(14). The lifetime fistula prevalence was 3.4 cases per 1000 reproductive age women of Ethiopia(15). According to those reports, Ethiopia may not only fail to eliminate OBF by 2025 but also might be unable to achieve the Sustainable Development Goals of eradicating this condition by 2030 (16).

Although OBF is treatable through surgery, access remains limited due to a shortage of skilled professionals, essential resources, and financial or awareness barriers preventing women from receiving care (5,18). And the recovery time from OBF in Ethiopia varies across hospitals ranging from 18.71 days to 5.19 weeks. Factors influencing recovery include socio-demographic conditions, delays in seeking care, cultural and physical barriers, antibiotic use, surgeon experience, and fistula related factors. It is also influenced by hospital settings, treatment approaches, and patient conditions (13)(19–23)(24).

Although fistula centers have been established in Ethiopia and the government has implemented strategic initiatives targeting risk reduction, identification and treatment, reintegration, and income generation for survivors, with the goal of eradicating OBF by 2025, the condition remains prevalent (25). Whilst surgical repair can address fistula in many women, complete recovery should involve more than just restoring continence (26). Delay in time to recovery from the OBF will affect the health care seeking behavior of the women and number of treated cases. Even though there are few studies on survival time of OBF conducted in the Northern and eastern Ethiopia, the recovery rate and median recovery time have not been well studied, and there is specifically no

evidence from the country's first and largest Fistula center. Therefore, this study will assess the recovery rate and median recovery time and identify factors that predict the recovery time from OBF at Hamlin Fistula Center in Addis Ababa. This helps to understand factors contributing to the recovery time of OBF and to reduce morbidity and mortality due to its complication by improving the care provided in the country's largest fistula hospital. The study also helps to support in achieving the national plan to eliminate OBF in Ethiopia. Therefore, this study assessed predictors of recovery time from OBF and its predictors at Addis Ababa Hamlin fistula hospital Ethiopia.

Methods

Study design, area and period

A Hospital based retrospective cohort study was employed among patients who were treated for OBF at Hamlin fistula center, Ethiopia. Hamlin Fistula Hospital Addis Ababa is the world's first fistula hospital which was founded by Dr. Catherine and Reg Hamlin in 1974. It is a charity organization established for the treatment and prevention of childbirth-related injuries in Ethiopian. The center treats around 200 patients each year, using a surgical technique and more than 50,000 patients were treated. Hamlin Fistula Ethiopia stands as a globally renowned center of excellence for treating OBF and training Midwives and specialties. The facility offers comprehensive rehabilitation services, including physiotherapy, counselling, and skills training (27). The study was conducted at Hamlin fistula center from July 27 to August 22, 2023.

Population

Random samples of OBF patients who were treated at Hamlin fistula center, from January 1st, 2017, to June 30th, 2023, were the study population. The study included patients with OBF who received treatment at the Hamlin fistula center in Addis Ababa, provided their medical records were complete and contained both admission and discharge dates.

Sample Size Determination and Sampling Technique

The sample size was determined using Open Epi considering the different assumptions: for a time to event comparison with a balanced ratio of unexposed to exposed (1:1), 95% confidence interval, 80% power and the anticipated proportion of the hazard of OBF recovery time among women. Damaged urethra (exposed) =20.7%, and those with non-damaged urethral status (unexposed) =79.3 % and AHR =1.59. The sample size was determined to be 417 for both the exposed and unexposed groups, resulting in a total of 834 participants. After accounting for a 10% non-response rate, the final sample size was found 918 (28).

Given that the study population available was limited to 945 individuals, which is below 10,000, we applied the finite population correction formula to adjust the sample size. By employing the standard correction formula, we calculated the sample size to be 466. Subsequently, after accounting for a 10% non-response rate, the final sample size was determined to be 513.

A simple random sampling technique was employed to select the study subjects using the registration numbers of patients registered at the Hamlin fistula center. From a total of 945 fistula patient records enrolled between January 2017 and June 2023, 513 samples were chosen based on their medical record numbers in the database. The relevant information's were then extracted from these selected records.

Study variables

Time to recovery from OBF in weeks is an outcome variable.

Predictor variables

Socio demographic factors include, woman age, residence, level of education, marital status body mass index, and age at marriage. Obstetric related factors which included, parity, place of delivery, delivery mode, labor duration.

Fistula-related characteristics were duration between the onset of the fistula and time of surgical treatment, width and length of fistula hole, pre- and post-operative care, repairing approach, type of fistula, duration of bladder catheterization, degree of vaginal scarring, presence of psychotherapy before and after surgery, and rehabilitation after surgery.

Operational definitions

Event: recovering from obstetric fistula

Recovered: were those patients who became cured from obstetric fistula.

Survival time: the time period in weeks from the initiation of treatment for obstetric fistula to documented clinical recovery. Women were followed from the start of treatment until recovery (29).

Censoring: a woman who does not experience the event(recovery from obstetric fistula) before the study ends, a person who died after being admitted for obstetric fistula treatment and patients whose recovery status was unknown at discharge (30).

Iatrogenic fistula: A fistula that is caused mostly by cesarean section and hysterectomy do to the complication of obstructed labor (31).

Data collection tool and technique

Data for the current study were collected by using English version checklist developed after reviewing results of different articles(5,12,36). The checklist was prepared in English then data were collected electronically using Kobo toolbox. A secondary data from hospital's patient file were used to collect data on OBF patients from the day of initial treatment for OBF to recovery at Hamlin fistula center. Two trained data collectors collected data for the study.

Data Quality Control and Management

In order to enhance the quality and understandability of data, training was provided for two midwife data collectors on how to use and collect data from the patient file. Pretest was done on records of 26 patients at Hamlin fistula center Addis Ababa among patients who were not considered in the final collected data. Accordingly, some modifications on the data extraction tool arrangements, removal and addition of some questions were made based on the feedback from the pre-testing. The data collection process was stringently supervised by the investigators. Consistency and accuracy of the collected data were conducted on a daily basis.

Data processing and analysis

Kobo Toolbox software was used for data collection, after which the data were exported to SPSS version 25 for statistical analysis. The data cleaning was done to check for missing values and outliers. Those women with incomplete record and data with significant missing values were excluded from the study and we analyzed a complete case analysis. For categorical data, descriptive statistics included frequencies and percentages, whilst for continuous data, the mean, standard deviation, median, and interquartile range were calculated. Survival analysis was employed to determine the recovery time and predictors affecting the time to recover from OBF.

The distribution of recovery time was assessed using the Kaplan-Meier estimator, while the log-rank test was applied to assess the recovery time experience across different categories of categorical variables (32). The model was tested by log minus log. Then, Cox regression was done to identify the predictors of time to recovery on those variables that pass the model fitness. Cox-Snell residuals are utilized to assess the overall goodness of fit of the model. The proportional hazard assumptions were checked using a log min log plot and through checking for multicollinearity using VIF (1.323). Variables which were found to have statistically significant

association with a p-value < 0.25 in the bivariate Cox proportional hazard model analysis were then analyzed using a multivariable Cox proportional hazard model. The 95% confidence interval of the hazard ratio was calculated, and a variable with p-value less than or equal to 0.05 in the multivariable Cox proportional hazards model were considered to be significant predictors of the time to recovery from OBF.

Ethical approval

The ethical approval of this study was secured from the Ethical Review Board of Ambo University, College of Health Sciences and Referral Hospital (reference number AU/CMHS-RCS/05/2023). A formal official letter of support was also written to Hamlin Fistula center. All study methods were performed in accordance with the relevant guidelines and regulations; the study was performed in accordance with the Declaration of Helsinki. As the current study relied on data collected from patient's record retrospectively, the requirement for the informed consent was waived by Ethical review board of College of Health Sciences and Referral Hospital, Ambo University. After submitting the proposal and explaining the study's purpose, benefits, and risks, permission and signed approval was also obtained from the Hamlin Fistula Hospital's administration. The study used existing patients' records or charts. Recognizing the sensitivity of the information, the trained data collectors took the patients' files to a separate room, where access was restricted to only the data collectors and the investigator. Once the data was collected, the records were placed back in their original place. We ensured patient confidentiality strictly through removing personal identifiers such as their names, addresses, phone numbers, or the names of healthcare providers. Additionally, the confidentiality of the data was ensured by using anonymous questionnaires and storing the collected information in a password-protected folder where only researchers can get access to it.

Results

Socio-demographic characteristics

In this study, 513 patients' cards were reviewed, and 495 patients were included, resulting in a response rate of 96.5%**.** About three-fifth 287 (58.0%) of the women were in the 19-30 years age group, with the mean age of 30.2 years with a standard deviation of 8.9 years. Additionally**,** 377 (76.2%) of the women were married, and nearly half 228 (46.1%) had given birth at or before the age of 18 years. In terms of education, 310 (62.6%) had no formal education. Half of them, 249 (50.3%)**,** were engaged in farming, and many of them 218 (44%)**,** were from Oromia region (**Table 1**).

Obstetric Related Factors

Among the 495 women studied, 118(23.8%) of them were grand multipara. The majority, 411(83%) of women delivered in a health facility, with 275(55.6%) of them gave birth through cesarean section. Regarding labor outcomes and duration, 342 (69.1%) women experienced stillbirths, and 261 (52.7%) encountered prolonged labor (**Table 2**).

Fistula Related Characteristics

Most of the fistula cases 303(61.2%) were developed at the age range between 19-30 years and 461(93.1%) of them took prophylactic antibiotics. All OBF patients had at least one form of inconsistence, of which 440(88.9%) of them had urinary inconsistence while only one patient got catheterization before surgery.

Regarding surgical history, data from 85 patients (17.2%) indicated they had undergone previous surgery due to a fistula, with only 6 (1.2%) having had three surgical procedures. Concerning type of fistula, most of them 431(87.1%) were Vesico-Vaginal Fistula (VVF), followed by 127(25.7%) mixed cases, and 52(10.5%) of them were from iatrogenic. Furthermore, 50(10.1%) of patients

had complete urethral distraction, while 87(17.6%) and 16(3.2%) of them had severe vaginal scarring and severe fibrosis respectively. A vaginal surgical approach was performed for 441(89.1%) patients. The results also showed that 370(74.7%) of the OBF patients recovered, while the remaining 125(25.3%) were censored (**Table 3**).

Time to recovery from obstetric fistula

The OBF patients were followed for a total of 5932 person-weeks at risk and under observation. The overall person time recovery rate from OBF was 6.34 per 100 person-weeks (95% CI: 5.7, 7.0).

Kaplan-Meir curve shows that recovery time estimates revealed that among the included patients 74.7%, 95%CI: (71.1, 78.2) were recovered from OBF. The median recovery time was 5.86 weeks, with an interquartile range (IQR) of 3.57 to 11.29 weeks (**Figure 1**). **Figure 1: Kaplan-Meier estimator curve for the recovery time of obstetric fistula patients at Hamlin fistula center Addis Ababa Ethiopia from January 2017 to June 2023.** Significant differences in Median recovery time were observed among those having different occupational status. The median recovery time among those who were engaged in farming activity was 6.9 weeks whereas those engaged in as government employee had 5 weeks median recovery time (p-value = 045). Furthermore, the long rank test revealed significant differences in recovery time based on the type of incontinence. The median recovery time was longer (7.4 weeks) if the women had mixed inconsistence while relatively shorter for those with fecal inconsistence (3.3 weeks) with p value 0.002.

According to the long rank test, a significant difference was observed in the recovery time between patients with and without rectovaginal fistula. Patients with rectovaginal fistula needed more time (7.1 weeks) to recover than those women don’t have rectovaginal fistula (5.7 weeks) (p-value of

0.014). Similarly, the median recovery time varied significantly depending on the severity of rectovaginal fibrosis. Patients with severe rectovaginal fibrosis required much longer (22.4 weeks) to recover than those with mild fibrosis (4 weeks), with a p value of 0.011 (**Figure 2**). **Figure 2: Survival graph for recto vaginal fibrosis on the recovery time among obstetric fistula patients at Hamlin fistula center Addis Ababa Ethiopia from January 2017 to June 2023.**

The median recovery time varied significantly depending on the surgery approach. Patients who underwent both vaginal and abdominal surgery needed more time to recover (10 weeks) than those who had only vaginal (5.9 weeks) or only abdominal (5.4 weeks) surgery, with a p value of 0.038. The recovery time also differed significantly depending on the urethral status. Patients with complete urethral damage required 8 weeks to recover, with a p value of 0.05.

Patients with severe vaginal scarring had a median recovery time of 9.7 weeks, while those without scarring recovered in 5.3 weeks (p value 0.000). The location of the fistula also affected the recovery time. Recto-Vaginal Fistula (RVF) patients, whose fistula was close to the hymen, needed 8.7 weeks to recover, while those whose fistula, was far from the hymen recovered in 3.1 weeks (p value 0.010). Moreover, the fistula distance from bladder influenced the recovery time. Patients with a fistula of less than 5 cm from bladder took 8.7 weeks to recover, while those with a larger distance from bladder recovered faster (p-value = 0.042) (**Table 4**) (**Figure 3**). **Figure 3: Survival graph for length of fistula on the recovery time among obstetric fistula patients at Hamlin fistula center Addis Ababa Ethiopia from January 2017 to June 2023.**

Cox Proportional Hazards (PH) Model adequacy assessment

The adequacy of the Cox proportional hazard model was evaluated using a log-minus-log plot for each variable prior to entering into the final model. For each covariate incorporated into the model, a plot was used to estimate the recovery period for patients suffering from OBF. The log minus log

plot assumption was satisfied for that covariate if the plot demonstrates random parallel or not cross each other's.

On plot checking: Body Mass Index (BMI), place of delivery, outcome of delivery, age at development of fistula in years, got physiotherapy after surgery, got psychotherapy before surgery, got psychotherapy after surgery, length and width of fistula, rectovaginal fistula and Status of urethra were predictors that satisfied the PH assumption while marital status, occupational status, educational status, distance from hymen, stage of vaginal scarring, fistula fibrosis and surgical approach were satisfied after adjustments made however getting ureteral catheterization before surgery, vesico-vaginal fistula and bladder size was removed due to violation of the assumption even after adjustments made.

Checking model fitness by using Cox-Snell residual plots

Cox-Snell residuals were used to assess the goodness of fit of the model. The log logistic Cox-Snell residual plot appears to align closely with a 45-degree line passing through the reference line. This shows the observed line closely aligns with the reference line across the majority of the distribution, with only minor deviations at higher residual values. Therefore, the log-logistic model adequately fits the data, indicating that the model assumptions are reasonably met (**Figure 4**).

Figure 4: The Cox-Snell residual plots for log-logistic, distributions of time to recovery from obstetric fistula patients at Hamlin fistula center Addis Ababa Ethiopia from January 2017 to June 2023. The Omnibus Test of Model Coefficients also revealed that the final Cox proportional hazards model provided a significantly better fit than the null model ($\chi^2 = 55.12$, df = 25, $p < 0.001$). This finding indicates that the predictors included in the model collectively explain a significant portion of the variation in recovery time among patients with obstetric fistula.

Predictors of recovery time from obstetric fistula

Cox regression analysis was employed in a survival analysis to determine the predictors of the recovery time of OBF. Twenty-two independent variables were examined using the Cox proportional hazards model, with recovery time as the dependent variable.

In the bivariate Cox proportional hazard analysis, variables with a p-value of 0.25 or less were included in a multivariate Cox proportional hazard regression analysis. Among these, only five variables showed statistical significance concerning recovery time, with a p-value of 0.05 or lower, along with their respective Adjusted Hazard Ratios (AHR) and 95% confidence intervals (CI).

The results showed that, patients with complete urethral distraction were 57% less likely to recover faster than those with no urethral distraction (AHR: 0.43, 95% CI: 0.25, 0.76). Patients who received physiotherapy after surgery were 1.85 times more likely to recover faster than those who did not receive physiotherapy (AHR: 1.85, 95% CI: 1.13, 3.02). Patients with a small fistula length (VVF) (2.2 cm to 4 cm) were 5.23 times more likely to recover faster than those with a large fistula length (more than or equal to 5 cm) (AHR: 5.23, 95% CI: 1.17, 12.33).

VVF patients with severe vaginal scarring were 40% less likely to recover faster than those with mild or no vaginal scarring (AHR: 0.60, 95% CI: 0.40, 0.89). RVF patients with mild fistula fibrosis were 39% less likely to recover faster than those with no fistula fibrosis (AHR: 0.61, 95% CI: 0.40, 0.84) (**Table 5**).

Discussions

The study aimed to determine the median recovery time and its influencing factors among patients with OBF. The findings indicated that OBF was healed in 74.7% (95%CI: 71.1, 78.2) of the patients, with the median duration of 5.86 weeks. The recovery rate is consistent with the study done in Gondar teaching hospital fistula center (73.7%) (20). However, this result was lower than studies conducted in other parts of Ethiopia, including another study at Gondar University

Teaching and referral hospital (88.07%) (22), Jimma University Medical Center (81.4%) (28) and Mekele Hamlin Fistula Center (89.33%) (21). This variation may be attributed to the differences in patient's demographic and socio-economic characteristics, care seeking behavior and access to timely care across facilities. Although the Hamlin Fistula Center in Addis Ababa is situated in the capital, it receives complex and chronic cases referred from across the country, often involving extensive tissue damage or coexisting complications. These factors may contribute to the relatively lower recovery rate observed compared to other centers that serve less complicated cases.

The study revealed that the median time of recovery for fistula patients was 5.9 weeks (95%CI=5.1,6.6), which was in line with the studies done in Gondar university teaching and referral hospital, which reported an average (median) time of recovery of 5.14 weeks (22), and Gondar teaching hospital fistula center, which reported a median time of recovery of 5.19 weeks(20). However, this study had a longer recovery time compared to the study done in Harar Hamlin Fistula Center, which reported 18.71 days (2.67 weeks)(23). The variation in recovery time and the use of mean and median time of recovery may be attributed to differences in the settings, particularly as the current facility serves as a referral center where patients present with diverse complications. Additionally, the health-seeking behavior of caregivers or parents may influence the recovery duration.

This study identified predictors that influenced the recovery time for OBF patients in Hamlin fistula center Addis Ababa. The time to recovery from OBF was significantly affected by five factors: physiotherapy after surgery, VVF length, urethral status, vaginal scaring stage, and rectal fistula fibrosis. Patients with complete urethral damage had a 57% lower chance of recovering faster than those without urethral damage. This result is in line with the study done in Harar Hamlin Fistula Center (23,28). This might be because repairing urethral fistula is a complicated process, which

requires restoring the surviving tissues as a flexible and functional organ, and controlling the urine passage at proper times. A nonfunctional and scarred urethra results in incontinence and longer recovery time.

The length of VVF affected the recovery time for VVF patients. Patients with fistula length 2.2-4cm had a 5.23 times higher chance of recovering faster than those with fistula length greater than or equal to 5cm. This suggests that the recovery time increased as the fistula length increased. This result was consistent with the study done in Gondar, northwest Ethiopia (22).

Additionally, patients with VVF and severe vaginal scarring had a 40% lower likelihood of faster recovery from OBF than those without vaginal scarring. This result was similar with the study done in Harar Hamlin Fistula Center (23). This might be because scarred tissue had a weak blood supply and healed slowly. Scared fistula was also difficult to separate from the adjacent structures like vagina and pubis, making it difficult to repair without tension. Moreover, scarred tissue could block access to the fistula site and allow for the use of unhealthy tissue to seal the hole. Scarring of the vagina could also keep the urethra open, impairing its healing process and normal function (33,34).

Furthermore, physiotherapy after surgery increased the recovery rate for OBF patients by 1.85 times compared to those who did not receive physiotherapy. This is consistent with study conducted in Mekele Hamlin Fistula Center, Ethiopia (21). Physiotherapy involves various activities such as physical exercise and electronic stimulation. This enhanced the blood flow and nourished the damaged tissue, making the healing process faster than usual. Additionally, fistula fibrosis affected the time to recover from fistula. RVF patients with mild fistula fibrosis had a 39% lower chance of recovering faster than those without fistula fibrosis. This might be because fibrosis was a tissue scarring that occurred as a pathological deviation from the normal wound healing

response. The scar resulted from increased and prolonged inflammation, leading to slow wound healing.

The findings of the current study underscore significant clinical implications for enhancing individualized rehabilitation services for patients with OBF, aiming to improve their recovery time and quality of life. Incorporating physiotherapy into the postoperative care of OBF patients and offering specialized care for women with urethral injuries or severe vaginal scarring can shorten the recovery time. Hospital administrators, health facilities, and healthcare providers must strengthen rehabilitation programs and adopt individualized women centered care for OBF patients.

As strengths the current study included data from randomly selected patients treated over seven years at a specialized fistula center and used survival analysis for time to recovery and its predictors, strengthening the reliability of findings. However, this study has several limitations. The study was based on retrospective design using secondary data, which limits accuracy and completeness of information from records. There is potential bias from incomplete documentation, data quality or inconsistent clinical recording practices across time. Furthermore, potential predictors for OBF such as household wealth, sociocultural factors, nutritional status, distance from health facilities, antenatal care follow-up and psychosocial supports were not collected in this study. The absence of these factors on patients' chart may have introduced unmeasured confounding, limiting ability to fully adjust for potential determinants influencing the time to recovery time from OBF. Additionally, since the study included only patients who received surgical treatment and had complete follow-up data, selection bias is possible, as women without care or incomplete records were excluded. This may affect generalizability of findings to all OBF patients in Ethiopia.

Conclusions

Based on the findings of this study, recovery rate of the entire cohort and median time of recovery was within the sphere of standard (74.7% recovery rate with median recovery time of 5.9 weeks).

Physiotherapy after surgery, VVF length vaginal scarring, status of urethra and RVF fibrosis were predictors of time of recovery from fistula. Therefore, the Hamlin Fistula centers administration and health professionals should facilitate access to physiotherapy after surgery for all OBF patients. Health professionals also must give due attention to those patients having large size fistula, damaged urethral status, severe vaginal scaring and severe fibrosis to facilitate faster recovery. Future research should prioritize the utilization of prospectively collected data to obtain more comprehensive information, including factors not addressed in this study.

List of abbreviations: AHR: Adjusted Hazard Ratios, BMI: Body Mass Index, OBF: Obstetric Fistula, RVF: Recto-Vaginal Fistula, VVF: Vesico-Vaginal Fistula.

Declarations

Availability of data and materials

The datasets used and/or analyzed during the current study are available as supporting information with this manuscript.

Competing interests

The authors declare that they have no competing interests.

Funding: Not applicable

Authors' contributions

HIO, DYG and GAB participated in conceptualization, design of methods, investigation, data analysis, supervision, visualization, writing original draft and subsequent drafts. All authors read and approved of the final manuscript.

Acknowledgments

We wish to express our heartfelt thanks to Ambo University for the support and opportunity to conduct this study. We would also like to thank Addis Ababa Hamlin fistula center for their

invaluable sharing of information and support they provided us during proposal development and data collection. Furthermore, we wish to express our gratitude to the data collectors for their commitment throughout the data collection process.

Reference

1. Ethiopia I and central stastical A (CSA). Central Statistical Agency (CSA) [Ethiopia] and ICF. Ethiopia Demographic and Health Survey . Addis Ababa,Ethiopia,and Rockvile,Maryland,USA. 2016. 134 p.
2. Heller A, Allman J, Stone G, Heller AW. Washington University Open Scholarship Interrogating the Superlative Sufferer : Experiencing Obstetric Fistula and Treatment Seeking in Niger. 2015;
3. United Nations. Intensifying efforts to end obstetric fistula. United Nations Gen Assem. 2016;(August):1–19.
4. Wall LL. Obstetric fistula is a “neglected tropical disease”. Vol. 6, PLoS neglected tropical diseases. United States; 2012. p. e1769.
5. Arrowsmith S, Hamlin EC, Wall LL. Obstructed labor injury complex: obstetric fistula formation and the multifaceted morbidity of maternal birth trauma in the developing world. Obstet Gynecol Surv. 1996 Sep;51(9):568–74.
6. Wall LL. Obstetric vesicovaginal fistula as an international public-health problem. Lancet [Internet]. 2006;368(9542):1201–9. Available from: https://www.sciencedirect.com/science/article/pii/S0140673606694762
7. UNFPA. Good practice on ending the health and human right tragedy of obstetric fistula. New York. 2013;(15).
8. FMOH. OBSTETRIC FISTULA SURVEILLANCE AND RESPONSE. 2022;
9. Animut M, Mamo A, Abebe L, Berhe MA, Asfaw S, Birhanu Z. “The sun keeps rising but darkness surrounds us”: a qualitative exploration of the lived experiences of women with obstetric fistula in Ethiopia. BMC Womens Health. 2019;19(1):37.
10. Ahmed S, Anastasi E, Laski L. Double burden of tragedy: Stillbirth and obstetric fistula. Lancet Glob Heal. 2016;4(2):e80–2.
11. Wall LL. Obstetric Fistula Is a “Neglected Tropical Disease.” PLoS Negl Trop Dis. 2012;6(8):8–10.
12. Ahmed S, Tunçalp Ö. Burden of obstetric fistula: From measurement to action. Lancet Glob Heal. 2015;3(5):e243–4.
13. Ali AH, Al-azzi RMS, Al-busaili T, Al-sorori TA. Predisposing Factors of Obstetric Fistula & its Surgical Outcomes, among Al-Sabeen maternal Hospital attendances, Sana ` a – Yemen. Hadhramout J Med Sci. 2015;4(1):350–6.
14. National Strategic Plan for. 2021;2025(December).
15. Deribe K, Fronterre C, Dejene T, Biadgilign S, Deribew A, Abdullah M, et al. Measuring the spatial heterogeneity on the reduction of vaginal fistula burden in Ethiopia between 2005 and 2016. Sci Rep. 2020;10(1):3–5.
16. United Nations. Intensifying efforts to end obstetric fistula within a decade: report of the Secretary-General [Internet]. < bound method Organization. get_name_with_acronym of< Organization: The …; 2024. Available from:

https://documents.un.org/doc/undoc/gen/n24/192/39/pdf/n2419239.pdf
17. Slinger G, Trautvetter L. Addressing the fistula treatment gap and rising to the 2030 challenge. Int J Gynecol Obstet. 2020;148(S1):9–15.
18. Bello OO, Morhason-Bello IO, Ojengbede OA. Nigeria, a high burden state of obstetric fistula: A contextual analysis of key drivers. Pan Afr Med J. 2020;36:1–12.
19. Getachew T. Survival Analysis of Time to Recovery from Obstetric Fistula: A Case Study at Yirgalem Hamlin Fistula Hospital, Ethiopia. J Biom Biostat. 2015;06(03).
20. Derso EA, Ayalew S, Eshete A, Wale M. Determinants of time to recovery from obstetric fistula by using the data of university of Gondar teaching hospital fistula center, Gondar – Ethiopia: A parametric survival regression analysis. Cogent Med. 2020;7(1).
21. Bihon AM, Meikena HK, Serka S. Survival Modeling on the Determinants of Time to Recovery from Obstetric Fistula : The Case of Mekelle Hamlin Fistula. 2022;2022.
22. Yismaw L, Alemu K, Addis A, Alene M. Time to recovery from obstetric fistula and determinants in Gondar university teaching and referral hospital, northwest Ethiopia.
23. Hussen S, Melese E. Time-to-recovery from obstetric fistula and associated factors: The case of Harar Hamlin Fistula Center. Ethiop J Heal Dev. 2017;31(2):85–95.
24. Hamlin Y, Hospital F, Getachew T, Taye A, Jabessa S. Survival Analysis of Time to Recovery from Obstetric Fistula : A Case. J Biometrics Biostattistics. 2015;6(3).
25. Federal Ministry of Health Ethiopia. National Strategic Plan for Elimination of obstetric Fistula [Internet]. Vol. 2025, Moh. 2021. Available from: https://www.moh.gov.et/sites/default/files/2024-07/National Strategic Plan for Elimination of Obstetric Fistula_2021-2025.pdf
26. Kopp DM, Wilkinson J, Bengtson A, Chipungu E, Pope RJ, Moyo M, et al. Fertility outcomes following obstetric fistula repair: A prospective cohort study. Reprod Health. 2017;14(1):1–8.
27. http://hamlin.org.au/.
28. A.S. A, G.M. A, M. T, A. H, B.G. A, A.C. E, et al. Recovery Time and Its Predictors among Women Admitted with Obstetric Fistula in Jimma University Medical Center Southwest, Ethiopia: A Retrospective Cohort Study. medRxiv [Internet]. 2022; Available from: https://www.medrxiv.org/%0Ahttp://ovidsp.ovid.com/ovidweb.cgi?T=JS&PAGE=reference&D=empp&NEWS=N&AN=2017166185
29. Brandon George, MSa, Samantha Seals, PhDb, and Inmaculada Aban P. NIH Public Access. PMC. 2014;21(4):686–94.
30. Singh R, Mukhopadhyay K. Survival analysis in clinical trials: Basics and must know areas. Perspect Clin Res. 2011;2(4):145.
31. Tebeu PM, Kongnyuy E, Rochat CH. Quality care for rectovaginal Obstetric Fistula . The Northern Cameroon experience : case series and a review of the literature. Nternational J Obstet Trauma Trauma. 2011;1(1).
32. Bradburn MJ, Clark TG, Love SB, Altman DG. utorial Paper Survival Analysis Part II : Multivariate data analysis – an introduction to concepts and methods. 2003;431–6.
33. Ambese TY, Gebre H, Berhe A, Fisseha G, Gufue ZH, Hailu NA, et al. Effect of vaginal scarring on the recovery of surgical repair of obstetric fistula in Northern Ethiopia. Int J Gynaecol Obstet Off organ Int Fed Gynaecol Obstet. 2023 Mar;160(3):915–25.
34. Kayondo M, Wasswa S, Kabakyenga J, Mukiibi N, Senkungu J, Stenson A, et al. Predictors and outcome of surgical repair of obstetric fistula at a regional referral hospital,

Mbarara, western Uganda. BMC Urol [Internet]. 2011;11(1):23. Available from: https://doi.org/10.1186/1471-2490-11-23

Table 1: socio-demographic characteristics of obstetric fistula patients admitted at Hamlin fistula center Addis Ababa Ethiopia 2023 (N=495).

Variables	Category	Frequency	Percent
Age of women	≤18 years	28	5.7
	19-30 years	287	58.0
	≥31 years	180	36.4
Age at first birth	≤18 age in years	228	46.1
	19-30 age in years	262	52.9
	≥31 age in years	5	1.0
Marital status	Married	377	76.2
	Separated	62	12.5
	Widowed and other	56	11.3
Educational status women	No formal Education	310	62.6
	Primary education (1-8)	127	25.7
	Secondary education (9-12)	33	6.7
	college and above	25	5.1
Region	Oromia	218	44.0
	Amhara	101	20.4
	SNNPR	94	19.0
	Addis Ababa and others	82	16.6
Occupation of the women	Farmer	249	50.3
	Housewife	173	34.9
	Governmental employees and others	73	14.7
BMI	Underweight	104	21.0
	Normal weight	323	65.3
	Overweight	68	13.7

Table 2: Obstetric characteristics of obstetric fistula patients admitted at Hamlin fistula center Addis Ababa Ethiopia 2023 (N=495).

Variables	Category	Frequency	Percent
Parity	Primiparous (1)	149	30.1
	Multipara (2-4)	228	46.1
	Grand multipara (≥5)	118	23.8
Place of delivery	Health facility	411	83.0

	Home delivery	84	17.0
Duration of labor for the index child in days	Not prolonged (≤12hr)	234	47.3
	Prolonged (>12hr)	261	52.7
Mode of delivery for the index child	Assisted vaginal delivery	37	7.5
	Spontaneous vaginal delivery	183	37.0
	cesarean section	275	55.6
Outcome of delivery	Alive	153	30.9
	Stillbirths	342	69.1

Table 3: Fistula related characteristics patients admitted at Hamlin fistula center Addis Ababa Ethiopia 2023 (N=495).

**Variables**	**Category**	**Frequency**	**Percent**
Age at development of fistula in years	≤18 age	57	11.5
	19-30 age	303	61.2
	≥31 age	135	27.3
Took prophylaxis's antibiotic	Yes	461	93.1
	No	34	6.9
Type of incontinency	Fecal	26	5.3
	Urinary	440	88.9
	Urinary Fecal	29	5.9
Duration of urinary incontinence in days	Short duration (<30 days)	44	8.9
	Medium duration (30-365 days)	370	74.7
	Long duration (>365 days)	81	16.4
Got physiotherapy before surgery	Yes	12	2.4
	No	483	97.6
Got physiotherapy after surgery	Yes	36	7.3
	No	459	92.7
Got psychotherapy before surgery	Yes	200	40.4
	No	295	59.6
Got psychotherapy after surgery	Yes	93	18.8
	No	402	81.2
Got any surgery for the fistula previously at any fistula center	Yes	85	17.2
	No	410	82.8
Frequency of previous surgery (n=85)	once	62	12.5
	Twice	17	3.4.0
	Three times	6	1.2
**Type and characteristics of fistula**			
Vesico-vaginal fistula (VVF) (n=495)	No	64	12.9
	Yes	431	87.1
Length of vesico-vaginal fistula (n=431)	2.2-4cm	355	82.4
	≥5cm	76	17.6
VVF size based on distance from bladder (n= 431)	<5cm	21	4.9
	>5cm	410	95.1
Width of the VVF hole (n= 431)	0-2cm	272	54.9

	2.2-4cm	103	23.8
	≥5cm	56	12.9
Rectovaginal fistula (RVF) (n=495)	No	443	89.5
	Yes	52	10.5
Distance from hymen for RVF (n= 495)	No RVF types	443	89.5
	0-2cm	32	6.5
	3-5cm	16	3.2
	>5cm	4	0.8
Iatrogenic fistula (n=495)	No	443	89.5
	Yes	52	10.5
Ureteric fistula (n= 495)	No	457	92.3
	Yes	38	7.7
Mixed fistula (n= 495)	No	368	74.3
	Yes	127	25.7
Status of urethra (n= 495)	Partially damaged	166	33.5
	Not damaged	279	56.4
	Complete urethral distraction	50	10.1
Stage of vaginal scaring (n= 495)	No vaginal scaring	98	19.8
	Mild	142	28.7
	Moderate	168	33.9
	Severe	87	17.6
Fistula fibrosis(recto) (n= 495)	No	443	89.5
	Mild	18	3.6
	Moderate	18	3.6
	Severe	16	3.2
Surgery Approach (n= 495)	Vaginal	441	89.1
	Both	17	3.4
	Abdominal	37	7.5
Duration of urethral catheterization after procedure in days (n= 495)	No	22	4.4
	1-5	333	67.3
	6-10	137	27.7
	>11	3	.6
Duration of day before treatment (n= 495)	≤1 year	348	70.3
	1-5 years	76	15.4
	>5 years	71	14.3
Outcomes (n= 495)	Censored	125	25.3
	Recovered	370	74.7

Table 4: Comparisons of time to recovery among different levels of predictor variables using log-rank test from obstetric fistula patients admitted at Hamlin fistula center Addis Ababa Ethiopia 2023 (N= 495).

**Variables**	**Category**	**Frequency (%)**	**Median recovery time (weeks)**	**Log rank test**	
				X2	P Value
Occupation	Farmer	249(50.3)	6.9	6.2	.045
	Housewife	173(34.9)	5.6		
	governmental employee and others	73(14.7)	5.0		
Type of inconsistence	Urinary	440(88.9)	6.0	12.0	.002
	Fecal	26(5.3)	3.3		
	Both	29(5.9)	7.4		
Fecal Inconsistence	No	441(89.1)	5.7	7.0	.008
	Yes	54(10.9)	8.7		
Recto vaginal fistula	No	443(89.5)	5.7	6.0	.014
	Yes	52(10.5)	7.1		
Recto vaginal fibrosis (n= 52)	Mild	18(34.6)	4.0	11.1	.011
	Moderate	18(34.6)	10.1		
	Severe	16(30.8)	22.4		
Surgery approach	Vaginal	441(89.1)	5.9	6.5	.038
	Abdominal	37(7.5)	5.4		
	Both	17(3.4)	10.0		
Status of urethra	Not damaged	279(56.4)	5.4	6.0	.050
	Partially damaged	166(33.5)	6.0		
	Completely damaged	50(10.1)	8.0		
Stage of vaginal scarring	No vaginal scaring	98(19.8)	5.3	21.9	.000
	Mild	142(28.7)	5.6		
	Moderate	168(33.9)	5.1		
	Severe	87(17.6)	9.7		
Distance from hymen RFV (n= 52)	0-2cm	32(61.5)	8.7	11.3	.010
	3-5cm	16(30.8)	5.9		
	>5cm	4(7.9)	3.1		

Table 5: Bivariate and multivariable cox proportional hazard ratio indicating predictors of recovery time from obstetric fistula patients admitted at Hamlin fistula center Addis Ababa Ethiopia 2023 (N= 495).

Variable	Category	Survival status Recovered	Censored	CHR95%CI	P value	AHR95%CI	P value
Marital status	Married	292	85	**1**		**1**	
	Others**	78	40	.81(0.63,1.04)	0.096	.98(0.74,1.29)	.866
Educational status	No formal education	226	84	**1**		**1**	
	Had formal education	144	41	1.06(0.86,1.31)	0.574	0.92(0.72,1.17)	.490
Occupation of the women	Farmers	176	73	**1**		**1**	
	Others***	194	52	1.15(0.94,1.41)	0.185	1.20(0.96,1.50)	.103
BMI	Normal weight	233	90	**1**		**1**	
	Abnormal weight	137	35	1.01(0.89,1.24)	.997	0.98(0.78,1.22)	.835
Place of Delivery	Health facility	316	95	1		1	
	Home delivery	54	30	.77(0.57,1.03)	.074	.90(0.65,1.24)	.513
Outcome of delivery	Alive	133	20	1		1	
	Stillbirths	237	105	.87(0.70,1.08)	.198	1.09(0.89,1.39)	.514
Age at development of fistula in years	<=18 age	35	22	1		1	
	19-30 age	222	81	1.11(0.78,1.59)	.569	.90(0.62,1.32)	.605
	>=31 age	113	22	1.41(0.96,2.06)	.077	1.09(0.72,1.65)	.680
Type of incontinency	Urinary	333	107	1			
	Both urinary and fecal	37	18	1.28(0.91,1.80)	0.156	1.23(0.86,1.75)	2.60
Got physiotherapy after surgery	Yes	21	15	1.78(1.14,2.79)	.011	1.85(1.13,3.02)*	**.015**
	No	349	110	1		1	
Got psychotherapy before surgery	Yes	142	58	1		1	
	No	228	67	1.17(0.95,1.45)	.144	0.99(0.78,1.24)	.901
Got psychotherapy after surgery	No	62	31	1		1	
	Yes	308	94	1.32(1.00,1.75)	0.05	1.15(0.86,1.55)	.344
Length of fistula of VVF	No VVF	11	53	1.44(0.98, 2.12)	0.06	1.18(0.67, 2.06)	
	2..2-4cm	266	89	1.17(0.87, 1.58)	0.29	5.23(1.17,12.33)*	**0.03**
	>=5cm	51	25	1		1	
Width of fistula hole	No VVF	51	11	1.40(0.93, 2.14)	0.12	0.99(0.53, 1.86)	0.994
	0-2cm	211	63	1.22(0.86, 1.72)	0.24	.512(0.12,2.29)	0.384
	2.2-4cm	69	34	1.09(0.74, 1.62)	0.65	1.01(0.53,1.92)	0.908
	>=5cm	39	17	1		1	
Rectovaginal fistula	No	339	104	1		1	
	Yes	31	21	.63(0.44,0.92	.016	.66(0.17,2.63)	.560
Distance from hymen	No RVF	335	104	1.43(0.85, 2.39)	0.181	1.15(0.31, 4.23)	0.83
	0-2cm	20	12	0.76(0.38, 1.51)	0.76	0.56(0.27, 1.16)	0.12
	≥3cm	15	9	1		1	
Status of urethra	Not damaged	226	53	1		1	
	Partially damaged	117	49	.93(0.74,1.16)	.527	0.99(0.78,1.28)	.994

	Complete urethral distraction	27	23	.61(0.41,0.91)	.016	.43(0.25,0.76)*	**.004**
Stage of vaginal scarring	Mild(no) vaginal scaring	210	30	1		1	
	Moderate vaginal scarring	122	46	.91(0.72,1.13)	.390	.98(0.76,1.27)	.882
	Severe vaginal scarring	38	49	.45(0.32,0.64)	.000	.60(0.40,0.89)*	**.012**
Fistula fibrosis(recto)	RVF not present	339	104	1		1	
	RFV present	31	21	.63(0.44,0.92)	.016	0.61(0.40,0.83)*	**0.02**
Surgery Approach	Vaginal	331	110	1		1	
	Abdominal or both	39	15	1.03(0.74,1.44)	.854	.97(0.52,1.80)	.920

Keys: *P-value ≤ 0.05, **separated, divorced, widowed and single, ***Housewife, Self-employed, government-employee, and student

List of supporting Information

SI 1: SPSS data set

 Click here to access/download;Figure;Fig 1.tif

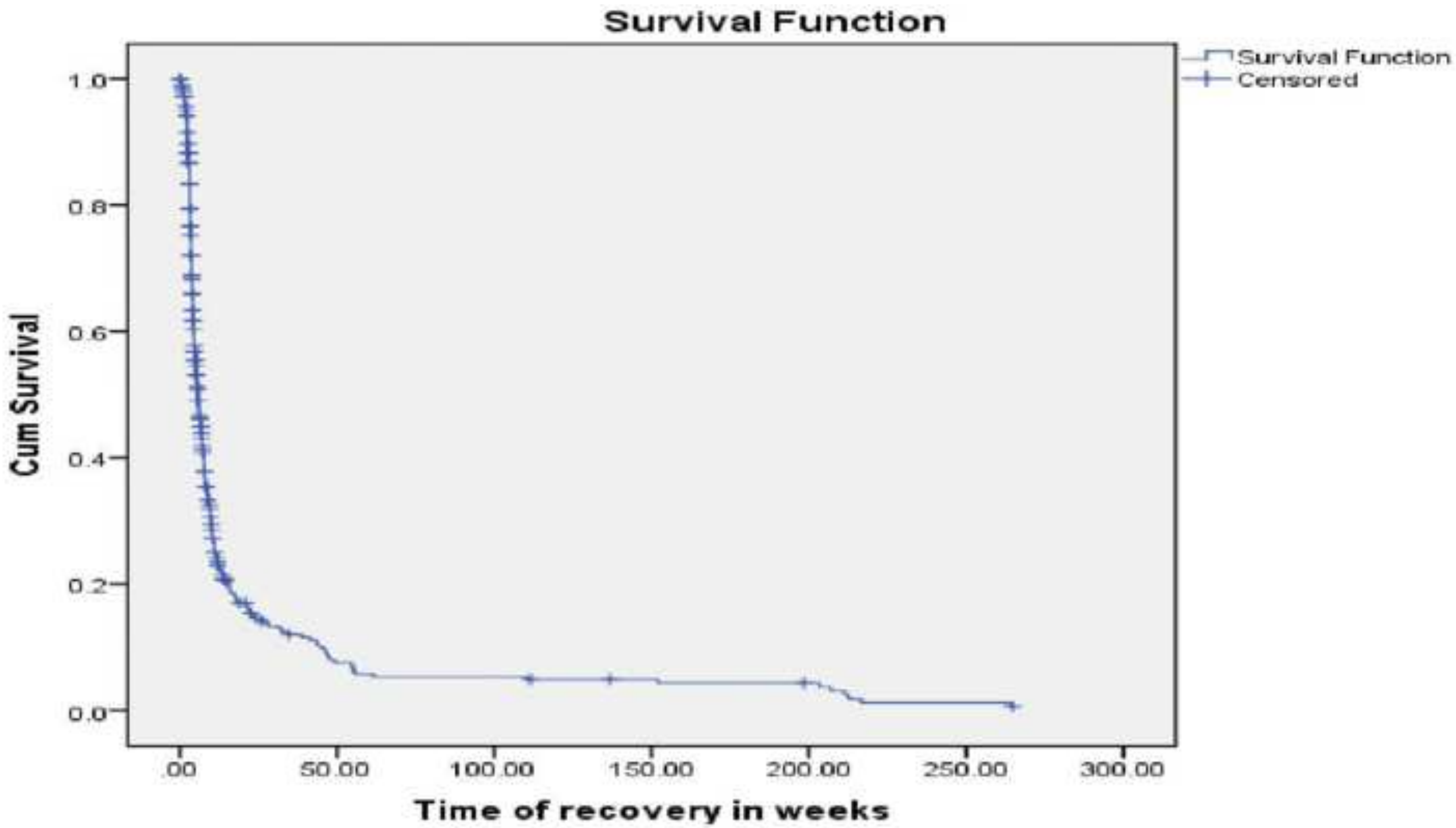

Figure 1: Kaplan-Meier estimator curve for the recovery time of obstetric fistula patients at Hamlin fistula center Addis Ababa Ethiopia from January 2017 to June 2023

Figure

Click here to access/download;Figure;Fig 2.tif

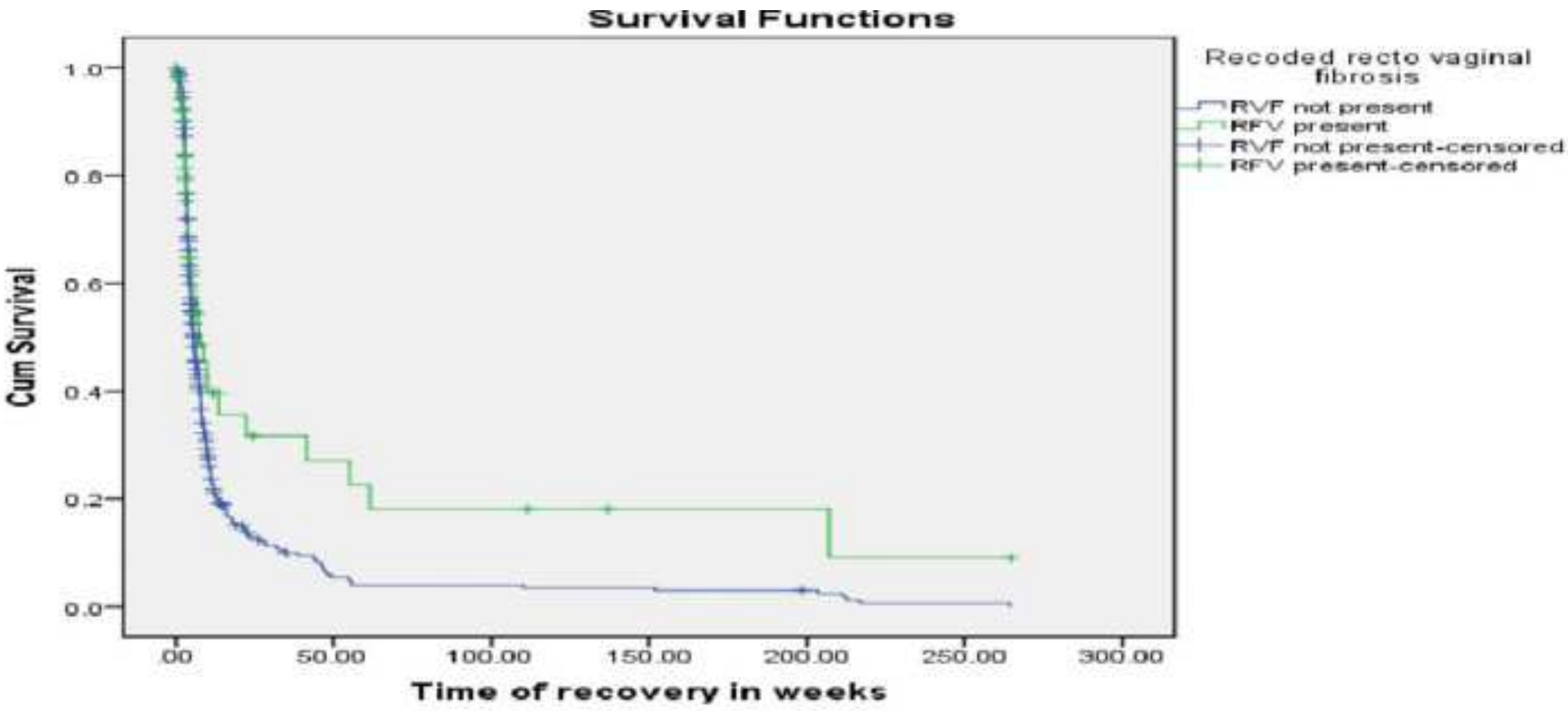

Figure 2: Survival graph for recto vaginal fibrosis on the recovery time among obstetric fistula patients at Hamlin fistula center Addis Ababa Ethiopia from January 2017 to June 2023

Figure

Click here to access/download;Figure;Fig 3.tif

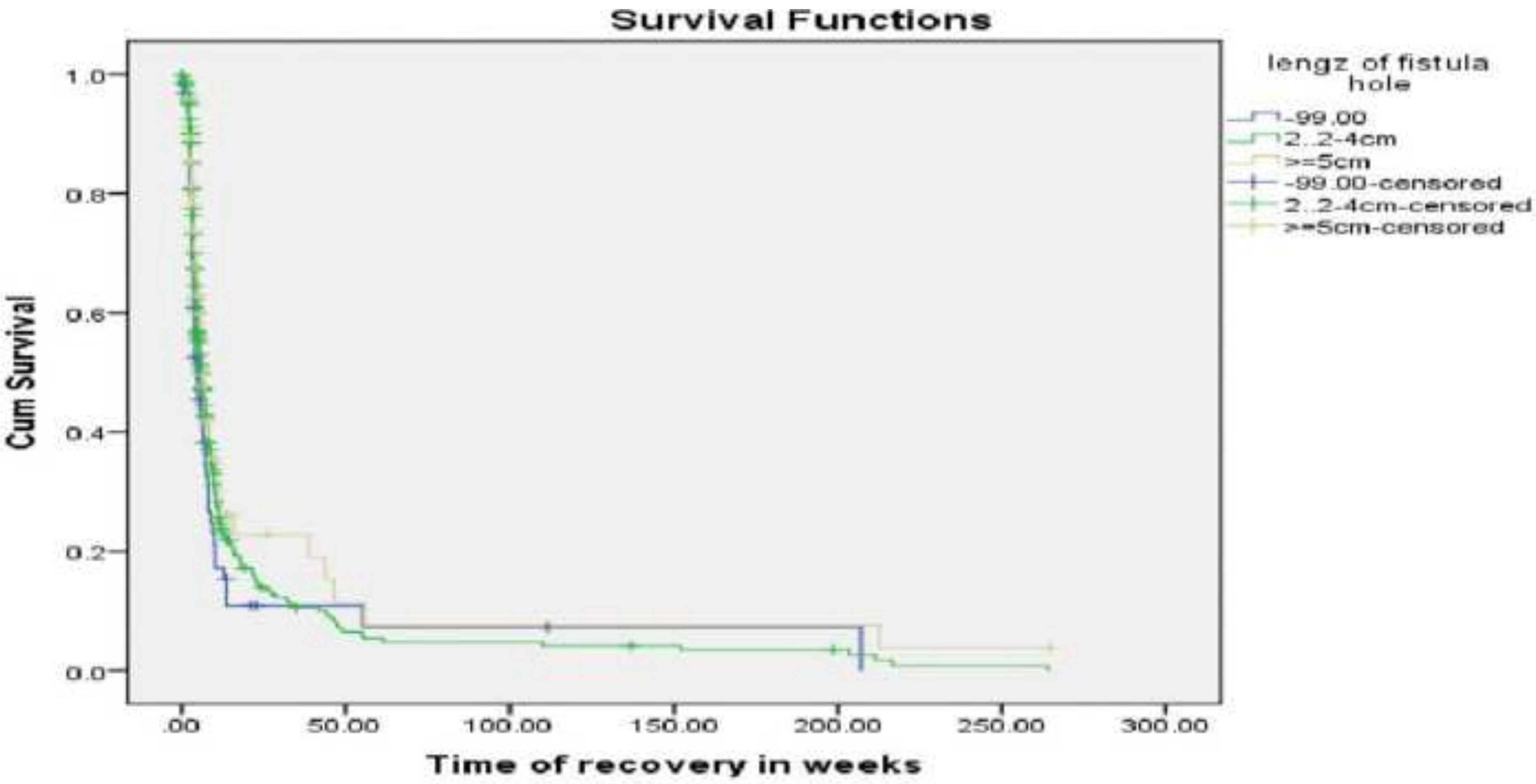

Figure 3: Survival graph for length of fistula on the recovery time among obstetric fistula patients at Hamlin fistula center Addis Ababa Ethiopia from January 2017 to June 2023

 Click here to access/download;Figure;Fig 4.tif

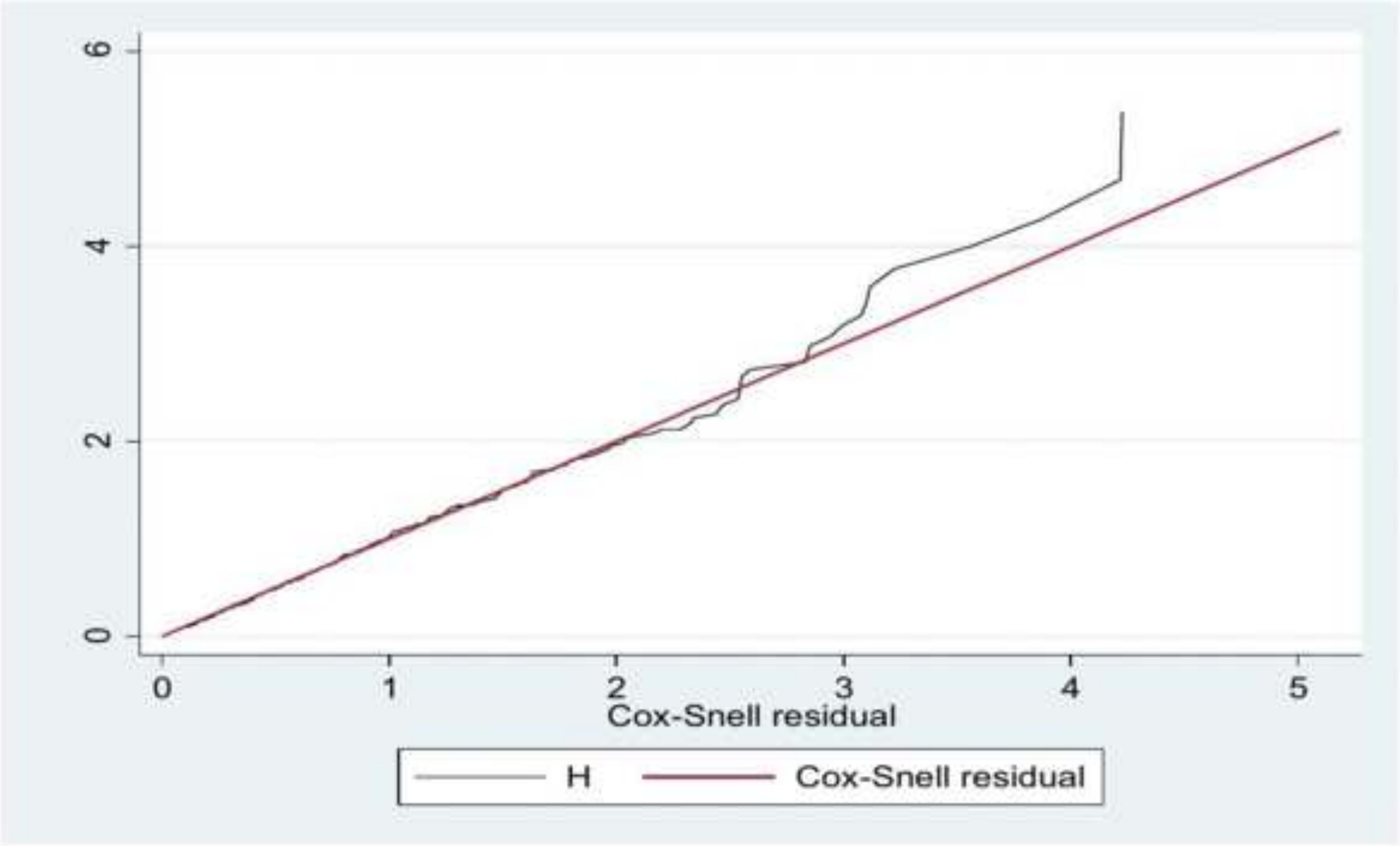

Figure 4: The Cox-Snell residual plots for log-logistic, distributions of time to recovery from obstetric fistula patients at Hamlin fistula center Addis Ababa Ethiopia from January 2017 to June 2023.

Click here to access/download
Supporting Information
Surv data OBF final.sav

Time to recovery from Obstetric Fistula and its Predictors among Obstetric Fistula patients admitted to Hamlin Fistula Center, Addis Ababa, Ethiopia: A Survival Analysis.

Formatted: Space After: 0 pt

Hayat Ibrahim Oumer[1], Delelegn Yilma Gebremichael[2], Gizachew Abdissa Bulto[3]*

[1]Addis Ababa City Health Bureau, Addis Ababa, Ethiopia.
[2]Ambo University, College of Health Sciences and Referral Hospital, Department of Public Health, Ambo, Ethiopia.
[3]Ambo University, College of Health Sciences and Referral Hospital, Department of Midwifery, Ambo, Ethiopia.

*Corresponding author

Address:
HIO: ihayat806@gmail.com
DYG: delelegnyilma@gmail.com
GAB: gizachab@yahoo.com

Formatted: Font: (Default) Times New Roman
Formatted: Font: (Default) Times New Roman
Formatted: Font: (Default) Times New Roman

Abstract

Obstetric Fistula (OBF) ~~The incidence of obstetric fistula (OBF)~~ remains a devastating maternal condition in Ethiopia, ~~high, posing~~causing significant ~~psychological,~~ physical suffering, social isolation ~~and health~~ and psychological traumas among affected women.~~challenges,~~ Despite its burden, only a limited number of healthcare facilities in Ethiopia provide surgical interventions. Furthermore, there is a scarcity of evidence regarding the time to recovery from OBF and its predictors at treatment centers. ~~yet only a limited number of health facilities in Ethiopia offer treatment services. Moreover, there is also a paucity of evidences on the time to recovery from OBF and its predictors at treatment centers. Therefore, it was aimed to identify the time to recovery from obstetric fistula and predictors among obstetric fistula patients admitted at Hamlin fistula hospital Addis Ababa, Ethiopia.~~ Therefore, this study aimed to determine the time to recovery from OBF and its predictors among patients admitted to Hamlin Fistula Hospital Addis Ababa, Ethiopia.

A Hospital based retrospective cohort study was employed among 513 women treated for OBF ~~obstetric fistula cases admitted~~ at Hamlin fistula hospital~~,~~ from January 1st, 2017, to June 30th 2023~~2023~~. A simple random sampling technique was employed to recruit 513 patient records and ~~. D~~data were ~~extracted from the patients' medical charts and~~ collected by using kobo toolbox software. The Kaplan-Meier method was employed to estimate the distribution of recovery time, and the log-rank test was utilized to assess the experience of recovery time among different levels of categorical variables. Bivariate and multivariable Cox proportional regression analyses were conducted, with statistical significance determined at a p-value < 0.05 and adjusted hazard ratio (AHR).

The overall recovery rate ~~of the entire cohort~~ was 74.7%, (95%CI: 71.1, 78.2), ~~and~~ with a median ~~time to~~ recovery time from ~~obstetric fistula~~OBF was 5.9 weeks (95%CI: ~~=~~5.1, 6.6). ~~Getting~~

Formatted: Font: 12 pt

Formatted: Font: 12 pt

Formatted: Font: 12 pt

Formatted: Font: 12 pt

Formatted: Font: 12 pt

Formatted: Font: 12 pt

Formatted: Font: 12 pt

Formatted: Superscript

~~physiotherapy after surgery (AHR:1.13,95%CI:1.13, 3.02), urethral status (damaged) (AHR: 0.43,95CI: 0.25,0.76), length of fistula (2.2. 4cm) (AHR:5.23, 95%CI: 1.17, 12.33), and having severe vaginal scarring (AHR: 0.60, 95%CI: 0.40, 0.89) were predictors of time to recovery from OBF.~~Receiving physiotherapy after surgery (AHR: 1.13, 95% CI: 1.13–3.02) and having a fistula length of 2.2–4 cm (AHR: 5.23, 95% CI: 1.17–12.33) were found to be predictors of faster recovery for OBF. Conversely, women with urethral damage (AHR: 0.43, 95% CI: 0.25–0.76) or severe vaginal scarring (AHR: 0.60, 95% CI: 0.40–0.89) experienced delayed recovery from OBF.

Based on the findings, median recovery time from OBF was 5.9 weeks. Therefore, strengthening postoperative physiotherapy and providing specialized care for women with urethral injury or severe vaginal scarring could improve recovery time from OBF. These findings also highlighted the critical importance of individualized rehabilitation services in fistula treatment centers to optimize patient recovery and overall quality of life.

~~**Getting physiotherapy after surgery, urethral status, having moderate and severe vaginal scarring were predictors that affect its time of recovery. Stakeholders such as Hamlin fistula centers and health workers should give due attention to the identified predictors for smooth and earlier time of recovery from OBF.**~~

Background

Obstetric Fistula (OBF) "is an abnormal opening or a hole between the vagina and urinary bladder or/and rectum or both that is resulted from prolonged and obstructed labor, surgical error, or trauma" (1)(2). Untreated, ~~obstetric fistula~~OBF leads to severe, lifetime morbidities with profound medical, psychological traumas and socio-economic consequences (3). –It is one of the most neglected human rights and public health problems, causing tremendous challenges such as

Formatted: Font: (Default) Times New Roman, 12 pt

Formatted: Font: 12 pt, Font color: Auto

Formatted: Font: (Default) Times New Roman, 12 pt

Formatted: Normal

Formatted: Font: (Default) Times New Roman

Formatted: Font: Bold

Formatted: Body Text

emotional distress, social isolation, family abandonment, and stigma. As reported by Animut et al., women with OBF experience profound social isolation, divorce, health problems, and psychological trauma. One woman expressed, “I am a person who is standing but not alive. […] However, I understand one thing darkness surrounds us despite the sun keeps rising” (crying). The majority (91%) of affected women experience stillbirths, while many also face anxiety, depression, discrimination, and worsening poverty. Common physical complications include nerve damage, foot drop, urinary and fecal incontinence, infertility, renal failure, and other pelvic disorders It is one of the neglected human rights and public health problems causing tremendous challenges including; emotional and social isolation, abandoned by their families, and stigmatized. The majority (91%) suffer stillbirths, while many face anxiety, depression, discrimination, and worsening poverty. Common physical complications include nerve damage, foot drop, incontinence, infertility, renal failure, and pelvic disorders (4–8)(9).

OBF occurs mainly in sub-Saharan Africa and Asia OBF is almost solely occurring in sub-Saharan Africa and Asia (10–12). Without timely access to emergency caesarean section, women with obstructed labor face higher risks of developing OBF Without timely access to an emergency caesarean section, women who developed obstructed labor is at higher risks of developing an OBF (3,13). Each year, 50,000-100,000 women worldwide develop OBF. In Sub-Saharan Africa and Southeast Asia, up to 3.5 million women may live with untreated OBF, potentially more due to unreported cases Each year, between 50,000 and 100,000 women worldwide are estimated to develop obstetric fistula. In Sub-Saharan Africa and Southeast Asia, it is estimated that up to 3.5 million women may be living with untreated OBF, with the actual figure potentially higher due to unreported cases (10). In Ethiopia, an estimated 72,533 women of reproductive age have

experienced fistula. Of these, 31,961 cases remain untreated, whilst 40,572 have received treatment. Annually, 948 new cases of OBF~~obstetric fistula~~ are reported, constituting approximately 1.6% of total obstructed and prolonged labor cases and 0.03% of total deliveries in 2020. The incidence of untreated vaginal fistula (VF) symptoms was also estimated to be 1.4 cases per 1000 in Ethiopia (14)(15). Although the government of Ethiopia in its strategic plan aimed to eliminate OBF~~obstetric fistula~~ by 2025, it remained a significant contributor to maternal morbidities. The point prevalence of OBF is 1.4 per 1000 women of childbearing age(14). The lifetime fistula prevalence was 3.4 cases per 1000 reproductive age women of Ethiopia(15). According to those reports, Ethiopia may not only fail to eliminate OBF by 2025 but also might be unable to achieve the Sustainable Development Goals of eradicating this condition by 2030 (16).

Although OBF ~~obstetric fistula~~ is treatable through surgery, access remains limited due to a shortage of skilled professionals, essential resources, and financial or awareness barriers preventing women from receiving care (5,18). And the recovery time from OBF ~~obstetric fistula~~ in Ethiopia varies across hospitals ranging from 18.71 days to 5.19 weeks. Factors influencing recovery include socio-demographic conditions, delays in seeking care, cultural and physical barriers, antibiotic use, surgeon experience, and fistula related factors. It is also influenced by hospital settings, treatment approaches, and patient conditions (13)(19–23)(24). Although fistula centers have been established in Ethiopia and the government has implemented strategic initiatives targeting risk reduction, identification and treatment, reintegration, and income generation for survivors, with the goal of eradicating OBF by 2025, the condition remains prevalent (25).

Formatted: Space Before: 0 pt, After: 0 pt

Formatted: Font: (Default) Times New Roman

~~Obstetric fistula is still prevalent in Ethiopia, yet its recovery rate and median time of recovery is not well studied.~~ Whilst surgical repair can address fistula in many women, complete recovery should involve more than just restoring continence (26). Delay in time to recovery from the OBF will affect the health care seeking behavior of the women and number of treated cases. ~~Moreover, t~~Even though there are ~~also limited~~few studies on survival time of OBF ~~obstetric fistula~~conducted in the Northern and eastern Ethiopia, the recovery rate and median recovery time have not been well studied, and there is specifically no evidence from the country's first and largest Fistula center. Therefore, ~~so~~ this study will assess the recovery rate and median recovery time and identify factors that predict the recovery time from OBF at Hamlin Fistula Center in Addis Ababa. This ~~help~~helps to understand factors contributing to the recovery time of OBF ~~obstetric fistula~~ and to reduce ~~the morbidity~~morbidity and mortality due to its complication by improving the care provided in the country's largest fistula hospital. The study also helps to support in achieving the national plan to eliminate OBF ~~obstetric fistula~~ in Ethiopia. Therefore, this study assessed predictors of recovery time from OBF and its predictors at Addis Ababa Hamlin fistula hospital Ethiopia.

Methods

Formatted: Space Before: 0 pt

Study design, area and period

A Hospital based retrospective cohort study was employed among patients who were treated for OBF ~~obstetric fistula~~ at Hamlin fistula center, Ethiopia. Hamlin Fistula Hospital Addis Ababa is the world's first fistula hospital which was founded by Dr. Catherine and Reg Hamlin in 1974. It is a charity organization established for the treatment and prevention of childbirth-related injuries in Ethiopian. The center treats around 200 patients each year, using a surgical technique and more than 50,000 patients ~~had been~~were treated. Hamlin Fistula Ethiopia stands as a globally renowned center of excellence for treating OBF and training Midwives and specialties. The facility offers

comprehensive rehabilitation services, including physiotherapy, counselling, and skills training (27). The study was conducted at Hamlin fistula center from July 27 to August 22, 2023.

Population

Formatted: Space Before: 0 pt

Random ~~sample~~samples of OBF ~~obstetric fistula~~ patients who were treated at Hamlin fistula center, from January 1st ~~2017~~, 2017, to June 30th ~~2023~~, 2023, were the study population. The study included patients with OBF who received treatment at the Hamlin fistula center in Addis Ababa, provided their medical records were complete and contained both admission and discharge dates.

Formatted: Space After: 0 pt

Sample Size Determination and Sampling Technique

Formatted: Space Before: 0 pt

The sample size was determined using Open Epi considering the different assumptions: for a time to event comparison with a balanced ratio of unexposed to exposed (1:1), ~~a~~ 95% confidence interval, 80% power~~, a balanced ratio of unexposed to exposed (1:1),~~ and the anticipated proportion of the hazard of OBF recovery time among women. Damaged urethra (exposed) =20.7%, and those with non-damaged urethral status (unexposed) =79.3 % and AHR =1.59. The sample size was determined to be 417 for both the exposed and unexposed groups, resulting in a total of 834 participants. ~~This sample size was found to be greater than the sample size calculated for other variables.~~ After accounting for a 10% non-response rate, the final sample size was found 918 (28). Given that the study population available was limited to 945 individuals, which is below 10,000, we applied the finite population correction formula to adjust the sample size. By employing the standard correction formula, we calculated the sample size to be 466. Subsequently, after accounting for a 10% non-response rate, the final sample size was determined to be 513.

~~Given that the study population was 945, which falls below 10,000, a correction formula was employed to adjust the sample size. Then the calculated sample was found to be 466. Subsequently, after considering 10% non-response rate, the final sample size was found to be 513.~~

Formatted: Space After: 0 pt, Don't adjust space between Latin and Asian text, Don't adjust space between Asian text and numbers

A simple random sampling technique was employed to select the study subjects using the registration numbers of patients registered at the Hamlin fistula center. From a total of 945 fistula patient records enrolled between January 2017 and June 2023, ~~495~~ 513 samples were chosen based on their medical record numbers in the database. The relevant information's were then extracted from these selected records.

Study variables

Time to recovery from OBF in weeks is an outcome variable

Predictor variables

Socio demographic factors ~~includes~~include, woman age, residence, level of education, marital status body mass index, and age at marriage. Obstetric related factors which included, parity, place of delivery, delivery mode, labor duration.

Fistula-related characteristics ~~were:~~were duration between the onset of the fistula and time of surgical treatment, width and length of fistula hole, pre- and post-operative care, repairing approach, type of fistula, duration of bladder catheterization, degree of vaginal scarring, presence of psychotherapy before and after surgery, and rehabilitation after surgery.

Operational definitions

Event: recovering from obstetric fistula

Recovered: were those patients who became cured from obstetric fistula.

Survival time: the time period in weeks from ~~a~~ the ~~starting treatment for obstetric fistula to recovery in weeks~~initiation of treatment for obstetric fistula to documented clinical recovery. Women were followed from the start of treatment until recovery (29).

Formatted: Space Before: 0 pt

Formatted: Space Before: 0 pt

Formatted: Space After: 0 pt

Censoring: a woman who does not experience the event(recovery from obstetric fistula) before the study ends, a person who died after being admitted for obstetric fistula treatment and patients whose recovery status was unknown at discharge (30).

Formatted: Space After: 0 pt

Iatrogenic fistula: A fistula that is caused mostly by cesarean section and hysterectomy do to the complication of obstructed labor (31).

Data collection tool and technique

Formatted: Space Before: 0 pt

Data for the current study were collected by using English version checklist developed after reviewing results of different articles(5,12,36). The checklist was prepared in English then data were collected electronically using Kobo ~~tool box~~toolbox. A secondary data from hospital's patient file were used to collect data on OBF patients from the day of initial treatment for OBF ~~obstetric fistula~~ to recovery at Hamlin fistula center. Two trained data collectors collected data for the study.

Data Quality Control and Management

Formatted: Space Before: 0 pt

In order to enhance the quality and understandability of data, training was provided for two midwife data collectors on how to use and collect data from the patient file. Pretest was done on records of 26 patients at Hamlin fistula center Addis Ababa among patients who were not considered in the final collected data. Accordingly, some ~~modification~~modifications on the data extraction tool arrangements, removal and addition of some questions were ~~done~~made based on the ~~feedbacks~~feedback from the pre-testing. The data collection process was stringently supervised by the investigators. Consistency and accuracy of the collected data were conducted on a daily basis.

Data processing and analysis

Formatted: Space Before: 0 pt

Kobo Toolbox software was used for data collection, after which the data were exported to SPSS version 25 for statistical analysis. The data cleaning was done to check for missing values and

outliers. Those women with incomplete record and data with significant missing values were excluded from the study and we analyzed a complete case analysis. For categorical data, descriptive statistics included frequencies and percentages, whilst for continuous data, the mean, standard deviation, median, and interquartile range were calculated. Survival analysis was employed to determine the recovery time and predictors affecting the time to recover from OBF. The distribution of recovery time was assessed using the Kaplan-Meier estimator, while the log-rank test was applied to assess the recovery time experience across different categories of categorical variables (32). The model was tested by log minus log. Then, Cox regression was done to identify the predictors of time to recovery on those variables that pass the model fitness. Cox-Snell residuals are utilized to assess the overall goodness of fit of the model. The proportional hazard assumptions were checked using a log min log plot and through checking for multicollinearity using VIF (1.323). Variables which were found to have statistically significant association with a p-value < 0.25 in the bivariate Cox proportional hazard model analysis were then analyzed using a multivariable Cox proportional hazard model. The 95% confidence interval of the hazard ratio was calculated, and a variable with p-value less than or equal to 0.05 in the multivariable Cox proportional hazards model were considered to be significant predictors of the time to recovery from OBF.

Ethical approval

The ethical approval of this study was secured from the Ethical Review Board of Ambo University, College of Health Sciences and Referral Hospital (reference number AU/CMHS-RCS/05/2023). A formal official letter of support was also written to Hamlin Fistula center. All study's method

~~was~~methods were performed in accordance with the relevant guidelines and regulations; the study was performed in accordance with the Declaration of Helsinki. As the current study relied on data collected from patient's record retrospectively, the requirement for the informed consent was waived by Ethical review board of College of Health Sciences and Referral Hospital, Ambo University. After submitting the proposal and explaining the study's purpose, benefits, and risks, permission and signed approval was also obtained from the Hamlin Fistula Hospital's administration. The study used existing patients' ~~record~~records or charts. Recognizing the sensitivity of the information, ~~T~~the trained data collectors took the patients' files to a separate room, where access was restricted to only the data collectors and the investigator. Once the data was collected, the records were placed back in their original place. We ensured ~~To maintain~~ patient confidentiality strictly through removing personal identifiers such as ~~, no information such as~~ their names, addresses, phone numbers, or the names of healthcare providers ~~was gathered~~. Additionally, the confidentiality of the data was ensured by using anonymous questionnaires and storing the collected information in a password-protected folder where only researchers can get access to it.

Results

Formatted: Space Before: 0 pt

Socio-demographic characteristics

In this study, 513 patients' ~~card~~cards were reviewed, and 495 patients were included, resulting in a response rate of 96.5%**.** About three-fifth 287 (58.0%) of the women were in the 19-30 years age group, with the mean age of 30.2 years with a standard deviation of 8.9 years. Additionally**,** 377 (76.2%) of the women were married, and nearly half ~~over half,~~ 228 (46.1%) had given birth at or before the age of 18 years. In terms of education, 310 (62.6%) had no formal education. Half of

Formatted: Space After: 0 pt

them, 249 (50.3%), were engaged in farming, and ~~the majority of~~many of them 218 (44%), were from Oromia region (**Table 1**).

Obstetric Related Factors

~~Out~~Among the ~~of~~ 495 women studied, ~~almost one third~~ 118(23.8%) of them were grand multipara. ~~While~~ The majority, 411(83%) of women delivered ~~at~~ in a health facility, with ~~and~~ 275(55.6%) of them gave birth through cesarean section. Regarding labor outcomes and duration, 342 (69.1%) women experienced stillbirths, and 261 (52.7%) encountered prolonged labor ~~Concerning the outcome and duration of labor 342(69.1%) had still birth while 261(52.7%) experienced prolonged labor~~ (**Table 2**).

Fistula Related Characteristics

Most of the fistula cases 303(61.2%) were developed at the age range between 19-30 years and 461(93.1%) of them took prophylactic antibiotics. All OBF patients had at least one form of inconsistence, of which 440(88.9%) of them had urinary inconsistence while only one patient got catheterization before surgery.

Regarding surgical history ~~of surgery~~, data from 85 patients (17.2%) indicated they had undergone previous surgery due to a fistula, with only 6 (1.2%) having had three surgical procedures. ~~85(17.2%) of patients' data reveled as they had previous surgery due to fistula case and only 6(7.1%) of them had three times surgical procedure.~~ Concerning type of fistula ~~case~~, most of them 431(87.1%) were Vesico-Vaginal Fistula (VVF), followed by 127(25.7%) mixed cases, and 52(10.5%) of them were from iatrogenic. Furthermore, 50(10.1%) of patients had complete urethral distraction, while 87(17.6%) and 16(3.2%) of them had severe vaginal scarring and severe

Formatted: Space Before: 0 pt

Formatted: Space After: 0 pt

Formatted: Space Before: 0 pt

Formatted: Space After: 0 pt

fibrosis respectively. A Vvaginal surgical approach was performed for 441(89.1%) patients. The results also showed that 370(74.7%) of the OBF patients were recoveredrecovered, while the remaining 125(25.3%) were censored (**Table 3**).

Formatted: Font:

Time to recovery from obstetric fistula

The OBF obstetric fistula patients were followed for a total of 5932 person-weeks at risk and under observation. The overall person time oftime recovery rate from OBF obstetric fistula was 6.34 per 100 person-weeks (95% CI: 5.7, 7.0).

Kaplan-Meir curve shows that recovery time estimates revealed that among the included patients 74.7%, 95%CI: (71.1, 78.2) were recovered from OBF. The median recovery time was 5.86 weeks, with an interquartile range (IQR) of 3.57 to 11.29 weeks (**Figure 1**). Significant difference onin Median recovery time waswere observed among those having different occupational status. The median recovery time among those who were engaged in farming activity was 6.9 weeks where aswhereas those engageengaged in as government employee had 5 weeks median recovery time (p-value = 045). Furthermore, the long rank test revealed significant differences in recovery time based on the type of incontinence. The median recovery time was longer (7.4 weeks) if the women had mixed inconsistence while relatively shorter for those with fecal inconsistence (3.3 weeks) with p value 0.002.

Formatted: Space Before: 0 pt

According to the long rank test, a significant difference was observed in the recovery time between patients with and without rectovaginal fistula. Patients with rectovaginal fistula needed more time (7.1 weeks) to recover than those women don’t have rectovaginal fistula (5.7 weeks) (p-value of 0.014). Similarly, the median recovery time varied significantly depending on the severity of rectovaginal fibrosis. Patients with severe rectovaginal fibrosis required much longer (22.4 weeks) to recover than those with mild fibrosis (4 weeks), with a p value of 0.011 (**Figure 2**).

The median recovery time varied significantly depending on the surgery approach. Patients who underwent both vaginal and abdominal surgery needed more time to recover (10 weeks) than those who had only vaginal (5.9 weeks) or only abdominal (5.4 weeks) surgery, with a p value of 0.038. The recovery time also differed significantly depending on the urethral status. Patients with complete urethral damage required 8 weeks to recover, with a p value of 0.05.

Patients with severe vaginal scarring had a median recovery time of 9.7 weeks, while those without scarring recovered in 5.3 weeks (p value 0.000). The location of the fistula also affected the recovery time. Recto-Vaginal Fistula (RVF) patients, whose fistula was close to the hymen needed 8.7 weeks to recover, while those whose fistula, was far from the hymen recovered in 3.1 weeks (p value 0.010). Moreover, the fistula distance from bladder influenced the recovery time. Patients with a fistula of less than 5 cm from bladder took 8.7 weeks to recover, while those with a larger distance from bladder recovered faster (p-value = 0.042) (**Figure 3**) (**Table 4**).

Cox Proportional Hazards (PH) Model adequacy assessment

The adequacy of the Cox proportional hazard model was evaluated using a log-minus-log plot for each variable prior to entering ~~in to~~into the final model. For each covariate incorporated into the model, a plot was used to estimate the recovery period for patients suffering from OBF~~obstetric fistula~~. The log minus log plot assumption was satisfied for that covariate if the plot demonstrates random parallel or not cross each other's.

Formatted: Space After: 0 pt

On plot checking: Body Mass Index (BMI), place of delivery, outcome of delivery, age at development of fistula in years, got physiotherapy after surgery, got psychotherapy before surgery, got psychotherapy after surgery, length and width of fistula, rectovaginal fistula and Status of urethra were predictors that satisfied the PH assumption while marital status, occupational status, educational status, distance from hymen, stage of vaginal scarring, fistula

fibrosis and surgical approach were satisfied after adjustments made however getting ureteral catheterization before surgery, vesico-vaginal fistula and bladder size was removed due to violation of the assumption even after adjustments made.

Checking model fitness by using Cox-Snell residual plots

Cox-Snell residuals were used are employed to assess the goodness of fit of the model. The log logistic Cox-Snell residual plot appears to align closely with a 45-degree line passing through the originreference line. This shows the observed line closely aligns with the reference line across the majority of the distribution, with only minor deviations at higher residual values. Therefore, the log-logistic model adequately fits the data, indicating that the model assumptions are reasonably met, indicating that the model is well-suited to the data (**Figure 4**). The Omnibus Test of Model Coefficients also revealed that the final Cox proportional hazards model provided a significantly better fit than the null model ($\chi^2 = 55.12$, df = 25, $p < 0.001$). This finding indicates that the predictors included in the model collectively explain a significant portion of the variation in recovery time among patients with obstetric fistula.

Predictors of recovery time from obstetric fistula

Cox regression analysis was employed in a survival analysis to determine the predictors of the recovery time of OBFobstetric fistula. Twenty-two independent variables were examined using the Cox proportional hazards model, with recovery time as the dependent variable.

Formatted: Space Before: 0 pt

In the bivariate Cox proportional hazard analysis, variables with a p-value of 0.25 or less were included in a multivariate Cox proportional hazard regression analysis. Among these, only five variables showed statistical significance concerning recovery time, with a p-value of 0.05 or lower, along with their respective Adjusted Hazard Ratios (AHR) and 95% confidence intervals (CI).

The results showed that, patients with complete urethral distraction were 57% less likely to recover faster than those with no urethral distraction (AHR: 0.43, 95% CI: 0.25, 0.76). Patients who received physiotherapy after surgery were 1.85 times more likely to recover faster than those who did not receive physiotherapy (AHR: 1.85, 95% CI: 1.13, 3.02). Patients with a small fistula length (VVF) (2.2 cm to 4 cm) were 5.23 times more likely to recover faster than those with a large fistula length (more than or equal to 5 cm) (AHR: 5.23, 95% CI: 1.17, 12.33).

VVF patients with severe vaginal scarring were 40% less likely to recover faster than those with mild or no vaginal scarring (AHR: 0.60, 95% CI: 0.40, 0.89). RVF patients with mild fistula fibrosis were 39% less likely to recover faster than those with no fistula fibrosis (AHR: 0.61, 95% CI: 0.40, 0.84) (**Table 5**).

Formatted: Space Before: 0 pt, After: 0 pt

Discussions

Formatted: Space Before: 0 pt

The study aimed to determine the median recovery time and its influencing factors among patients with OBF. The findings indicated that OBF was healed in 74.7% (95%CI: 71.1, 78.2) of the patients, with the median duration of 5.86 weeks. ~~The study aimed to measure the median time of recovery and its influencing factors among patients with obstetric fistula. The result showed that obstetric fistula was healed in 74.7%, 95%CI :(71.1, 78.2) of the patients within a median of 5.86 weeks.~~ Th e~~is~~ recovery rate is consistent with ~~was almost the same as~~ the study~~one reported by study~~ done in Gondar teaching hospital fistula center (~~, which was~~ 73.7%) (20).

~~Nevertheless~~However, this result was lower than studies conducted in other parts of Ethiopia, ~~such as:~~including another ~~-~~study at Gondar University Teaching and referral hospital (88.07%) (22), Jimma University Medical Center (81.4%) (28) and Mekele Hamlin Fistula Center (89.33%) (21). This variation may be attributed to the differences in patient’s demographic and socio-economic characteristics, care seeking behavior and access to timely care across facilities. Although the

Hamlin Fistula Center in Addis Ababa is situated in the capital, it receives complex and chronic cases referred from across the country, often involving extensive tissue damage or coexisting complications. These factors may contribute to the relatively lower recovery rate observed compared to other centers that serve less complicated cases.

The variations in the recovery rates among the studies might be due to the differences in demographic, socio-economic, environmental and health related factors, the quality of care provided in each hospital, the health seeking behavior and the availability of different medications and therapies for fistula treatment. Furthermore, Hamlin fistula center receive patients from different geographical location which having different socio-demographic characteristics that could affect recovery rate.

Formatted: Space After: 0 pt

The study revealed that the median time of recovery for fistula patients was 5.9 weeks (95%CI=5.1,6.6), which was in line with the studies done in Gondar university teaching and referral hospital, which reported an average (median) time of recovery of 5.14 weeks (22), , and Gondar teaching hospital fistula center, which reported a median time of recovery of 5.19 weeks(20). However, this study had a longer recovery time compared to than the study done in Harar Hamlin Fistula Center, which reported 18.71 days (2.67 weeks)(23). The variation in recovery time and the use of mean and median time of recovery may be attributed to differences in the settings, particularly as the current facility serves as a referral center where patients present with diverse complications. Additionally, the health-seeking behavior of caregivers or parents may influence the recovery duration.

This study identified predictors that influenced the recovery time for OBF obstetric fistula patients in Hamlin fistula center Addis Ababa. The time to recovery from OBF obstetric fistula was significantly affected by five factors: physiotherapy after surgery, VVF length, urethral status,

vaginal scaring stage, and rectal fistula fibrosis. Patients with complete urethral damage had a 57% lower chance of recovering faster than those without urethral damage. This result ~~was~~ is in line with the study done in Harar Hamlin Fistula Center (23,28). This might be because repairing urethral fistula is a complicated process, which requires restoring the surviving tissues as a flexible and functional organ, and controlling the urine passage at proper times. A nonfunctional and scarred urethra results in incontinence and longer recovery time.

The length of VVF affected the recovery time for VVF patients. Patients with fistula length 2.2-4cm had a 5.23 times higher chance of recovering faster than those with fistula length greater than or equal to ~~≥~~5cm. This suggest~~ed~~s that the recovery time increased as the fistula length increased. This result was consistent with the study done in Gondar, northwest Ethiopia (22).

Additionally, patients with VVF and severe vaginal scarring had a 40% lower likelihood of faster recovery from OBF ~~obstetric fistula~~ than those without vaginal scarring. This result was similar with the study done in Harar Hamlin Fistula Center (23). This might be because scarred tissue had a weak blood supply and heal~~ed~~s slowly. Scarred fistula was also difficult to separate from the adjacent structures like vagina and pubis, making it difficult to repair without tension. Moreover, scarred tissue could block ~~the access~~access to the fistula site and also ~~let~~allow for the use of unhealthy tissue to seal the hole. Scarring of the vagina could also keep the urethra open, impairing its healing process and normal function (33,34).

Furthermore, physiotherapy after surgery increased the recovery rate for OBF ~~obstetric fistula~~ patients by 1.85 times compared to those who did not receive physiotherapy. This ~~was~~ is consistent with study conducted in Mekele Hamlin Fistula Center, Ethiopia (21)~~.~~. Physiotherapy ~~involved~~involves various activities such as physical exercise and electronic stimulation. This enhanced the blood flow and nourished the damaged tissue, making the healing process faster than

usual. Additionally, fistula fibrosis affected the time to recover from fistula. RVF patients with mild fistula fibrosis had a 39% lower chance of recovering faster than those without fistula fibrosis. This might be because fibrosis was a tissue scarring that occurred as a pathological deviation from the normal wound healing response. The scar resulted from increased and prolonged inflammation, leading to slow wound healing.

The findings of the current study underscore significant clinical implications for enhancing individualized rehabilitation services for patients with OBF, aiming to improve their recovery time and quality of life. Incorporating physiotherapy into the postoperative care of OBF patients and offering specialized care for women with urethral injuries or severe vaginal scarring can shorten the recovery time. Hospital administrators, health facilities, and healthcare providers must strengthen rehabilitation programs and adopt individualized women centered care for OBF patients.

As strengths the current study included data from randomly selected patients treated over seven years at a specialized fistula center and used survival analysis for time to recovery and its predictors, strengthening the reliability of findings. However, this study has several limitations. The study was based on retrospective design using secondary data, which limits accuracy and completeness of information from records. There is potential bias from incomplete documentation, data quality or inconsistent clinical recording practices across time. Furthermore, potential predictors for OBF such as household wealth, sociocultural factors, nutritional status, distance from health facilities, antenatal care follow-up and psychosocial supports were not collected in this study. The absence of these factors on patients' chart may have introduced unmeasured confounding, limiting ability to fully adjust for potential determinants influencing the time to recovery time from OBF. Additionally, since the study included only patients who received surgical treatment and had complete follow-up data, selection bias is possible, as women without care or incomplete records were excluded. This may affect generalizability of findings to all OBF patients in Ethiopia.As a strength the current study included randomly selected patients who get treatment in seven years period. But the data were merely based on secondary data, so analysis of

Formatted: Normal, Line spacing: 1.5 lines, Tab stops: Not at 6.5"

Formatted: Font: (Default) Times New Roman, 12 pt

predictors for obstetric fistula recovery time was limited by the information that could be obtained from the patients' charts. Potential bias due to failing to address variables like,household wealth index, socioeconomic status that might have an effect on recovery, were some of the limitations of the current study.

Conclusions

Based on the findings of this study, recovery rate of the entire cohort and median time of recovery was within the sphere of standard (74.7% recovery rate with median recovery time of 5.9 weeks). Physiotherapy after surgery, VVF length vaginal scarring, status of urethra and RVF fibrosis were predictors of time of recovery from fistula. Therefore, the Hamlin Fistula centers administration and health professionals should facilitate access to physiotherapy after surgery for all OBFobstetric fistula patients. Health professionals also have tomust give due attention for those patients having large size fistula, damaged urethral status, having severe vaginal scaring and severe fibrosis to facilitate faster recovery. Future research should prioritize the utilization of prospectively collected data to obtain more comprehensive information, including factors not addressed in this study.

Formatted: Space After: 0 pt

List of abbreviations: AHR: Adjusted Hazard Ratios, BMI: Body Mass Index, OBF: Obstetric Fistula, RVF: Recto-Vaginal Fistula, VVF: Vesico-Vaginal Fistula.

Formatted: Space Before: 0 pt

Declarations

Availability of data and materials

The datasets used and/or analyzed during the current study are available from the corresponding author on reasonable requestas supporting information with this manuscript.

Competing interests

The authors declare that they have no competing interests.

Funding: Not applicable

Authors' contributions

HIO, DYG and GAB participated in conceptualization, design of methods, investigation, data analysis, supervision, visualization, writing original draft and subsequent drafts. All authors read and approved the final manuscript.

Acknowledgments

We wish to express our heartfelt thanks to Ambo University for the support and opportunity to conduct this study. We would also like to thank Addis Ababa Hamlin fistula center for their invaluable sharing of information and support they provided us during proposal development and data collection. Furthermore, we wish to express our gratitude to the data collectors for their commitment throughout the data collection process.

Reference

Formatted: Space Before: 0 pt

1. Ethiopia I and central stastical A (CSA). Central Statistical Agency (CSA) [Ethiopia] and ICF. Ethiopia Demographic and Health Survey . Addis Ababa,Ethiopia,and Rockvile,Maryland,USA. 2016. 134 p.
2. Heller A, Allman J, Stone G, Heller AW. Washington University Open Scholarship Interrogating the Superlative Sufferer : Experiencing Obstetric Fistula and Treatment Seeking in Niger. 2015;
3. United Nations. Intensifying efforts to end obstetric fistula. United Nations Gen Assem. 2016;(August):1–19.
4. Wall LL. Obstetric fistula is a “neglected tropical disease”. Vol. 6, PLoS neglected tropical diseases. United States; 2012. p. e1769.
5. Arrowsmith S, Hamlin EC, Wall LL. Obstructed labor injury complex: obstetric fistula formation and the multifaceted morbidity of maternal birth trauma in the developing world. Obstet Gynecol Surv. 1996 Sep;51(9):568–74.
6. Wall LL. Obstetric vesicovaginal fistula as an international public-health problem. Lancet [Internet]. 2006;368(9542):1201–9. Available from: https://www.sciencedirect.com/science/article/pii/S0140673606694762
7. UNFPA. Good practice on ending the health and human right tragedy of obstetric fistula. New York. 2013;(15).
8. FMOH. OBSTETRIC FISTULA SURVEILLANCE AND RESPONSE. 2022;
9. Animut M, Mamo A, Abebe L, Berhe MA, Asfaw S, Birhanu Z. “The sun keeps rising but darkness surrounds us”: a qualitative exploration of the lived experiences of women with obstetric fistula in Ethiopia. BMC Womens Health. 2019;19(1):37.
10. Ahmed S, Anastasi E, Laski L. Double burden of tragedy: Stillbirth and obstetric fistula. Lancet Glob Heal. 2016;4(2):e80–2.

11. Wall LL. Obstetric Fistula Is a “Neglected Tropical Disease.” PLoS Negl Trop Dis. 2012;6(8):8–10.
12. Ahmed S, Tunçalp Ö. Burden of obstetric fistula: From measurement to action. Lancet Glob Heal. 2015;3(5):e243–4.
13. Ali AH, Al-azzi RMS, Al-busaili T, Al-sorori TA. Predisposing Factors of Obstetric Fistula & its Surgical Outcomes, among Al-Sabeen maternal Hospital attendances, Sana ` a – Yemen. Hadhramout J Med Sci. 2015;4(1):350–6.
14. National Strategic Plan for. 2021;2025(December).
15. Deribe K, Fronterre C, Dejene T, Biadgilign S, Deribew A, Abdullah M, et al. Measuring the spatial heterogeneity on the reduction of vaginal fistula burden in Ethiopia between 2005 and 2016. Sci Rep. 2020;10(1):3–5.
16. United Nations. Intensifying efforts to end obstetric fistula within a decade: report of the Secretary-General [Internet]. < bound method Organization. get_name_with_acronym of< Organization: The …; 2024. Available from: https://documents.un.org/doc/undoc/gen/n24/192/39/pdf/n2419239.pdf
17. Slinger G, Trautvetter L. Addressing the fistula treatment gap and rising to the 2030 challenge. Int J Gynecol Obstet. 2020;148(S1):9–15.
18. Bello OO, Morhason-Bello IO, Ojengbede OA. Nigeria, a high burden state of obstetric fistula: A contextual analysis of key drivers. Pan Afr Med J. 2020;36:1–12.
19. Getachew T. Survival Analysis of Time to Recovery from Obstetric Fistula: A Case Study at Yirgalem Hamlin Fistula Hospital, Ethiopia. J Biom Biostat. 2015;06(03).
20. Derso EA, Ayalew S, Eshete A, Wale M. Determinants of time to recovery from obstetric fistula by using the data of university of Gondar teaching hospital fistula center, Gondar – Ethiopia: A parametric survival regression analysis. Cogent Med. 2020;7(1).
21. Bihon AM, Meikena HK, Serka S. Survival Modeling on the Determinants of Time to Recovery from Obstetric Fistula : The Case of Mekelle Hamlin Fistula. 2022;2022.
22. Yismaw L, Alemu K, Addis A, Alene M. Time to recovery from obstetric fistula and determinants in Gondar university teaching and referral hospital, northwest Ethiopia.
23. Hussen S, Melese E. Time-to-recovery from obstetric fistula and associated factors: The case of Harar Hamlin Fistula Center. Ethiop J Heal Dev. 2017;31(2):85–95.
24. Hamlin Y, Hospital F, Getachew T, Taye A, Jabessa S. Survival Analysis of Time to Recovery from Obstetric Fistula : A Case. J Biometrics Biostattistics. 2015;6(3).
25. Federal Ministry of Health Ethiopia. National Strategic Plan for Elimination of obstetric Fistula [Internet]. Vol. 2025, Moh. 2021. Available from: https://www.moh.gov.et/sites/default/files/2024-07/National Strategic Plan for Elimination of Obstetric Fistula_2021-2025.pdf
26. Kopp DM, Wilkinson J, Bengtson A, Chipungu E, Pope RJ, Moyo M, et al. Fertility outcomes following obstetric fistula repair: A prospective cohort study. Reprod Health. 2017;14(1):1–8.
27. http://hamlin.org.au/.
28. A.S. A, G.M. A, M. T, A. H, B.G. A, A.C. E, et al. Recovery Time and Its Predictors among Women Admitted with Obstetric Fistula in Jimma University Medical Center Southwest, Ethiopia: A Retrospective Cohort Study. medRxiv [Internet]. 2022; Available from: https://www.medrxiv.org/%0Ahttp://ovidsp.ovid.com/ovidweb.cgi?T=JS&PAGE=reference&D=empp&NEWS=N&AN=2017166185

29. Brandon George, MSa, Samantha Seals, PhDb, and Inmaculada Aban P. NIH Public Access. PMC. 2014;21(4):686–94.
30. Singh R, Mukhopadhyay K. Survival analysis in clinical trials: Basics and must know areas. Perspect Clin Res. 2011;2(4):145.
31. Tebeu PM, Kongnyuy E, Rochat CH. Quality care for rectovaginal Obstetric Fistula . The Northern Cameroon experience : case series and a review of the literature. Nternational J Obstet Trauma Trauma. 2011;1(1).
32. Bradburn MJ, Clark TG, Love SB, Altman DG. utorial Paper Survival Analysis Part II : Multivariate data analysis – an introduction to concepts and methods. 2003;431–6.
33. Ambese TY, Gebre H, Berhe A, Fisseha G, Gufue ZH, Hailu NA, et al. Effect of vaginal scarring on the recovery of surgical repair of obstetric fistula in Northern Ethiopia. Int J Gynaecol Obstet Off organ Int Fed Gynaecol Obstet. 2023 Mar;160(3):915–25.
34. Kayondo M, Wasswa S, Kabakyenga J, Mukiibi N, Senkungu J, Stenson A, et al. Predictors and outcome of surgical repair of obstetric fistula at a regional referral hospital, Mbarara, western Uganda. BMC Urol [Internet]. 2011;11(1):23. Available from: https://doi.org/10.1186/1471-2490-11-23

Formatted: Space After: 0 pt

Table 1: socio-demographic characteristics of obstetric fistula patients admitted at Hamlin fistula center Addis Ababa Ethiopia 2023 (N=495).

Variables	Category	Frequency	Percent
Age of women	≤18 years	28	5.7
	19-30 years	287	58.0
	≥31 years	180	36.4
Age at first birth	≤18 age in years	228	46.1
	19-30 age in years	262	52.9
	≥31 age in years	5	1.0
Marital status	Married	377	76.2
	Separated	62	12.5
	Widowed and other	56	11.3
Educational status women	No formal Education	310	62.6
	Primary education (1-8)	127	25.7
	Secondary education (9-12)	33	6.7
	college and above	25	5.1
Region	Oromia	218	44.0
	Amhara	101	20.4
	SNNPR	94	19.0
	Addis Ababa and others	82	16.6

Occupation of the women	Farmer	249	50.3
	~~House wife~~Housewife	173	34.9
	Governmental ~~employee~~employees and others	73	14.7
BMI	Underweight	104	21.0
	Normal weight	323	65.3
	Overweight	68	13.7

Table 2: Obstetric characteristics of obstetric fistula patients admitted at Hamlin fistula center Addis Ababa Ethiopia 2023 (N=495).

Variables	Category	Frequency	Percent
Parity	Primiparous (1)	149	30.1
	Multipara (2-4)	228	46.1
	Grand multipara (≥5)	118	23.8
Place of delivery	Health facility	411	83.0
	Home delivery	84	17.0
Duration of labor for the index child in days	Not prolonged (≤12hr)	234	47.3
	Prolonged (>12hr)	261	52.7
Mode of delivery for the index child	Assisted vaginal delivery	37	7.5
	Spontaneous vaginal delivery	183	37.0
	cesarean section	275	55.6
Outcome of delivery	Alive	153	30.9
	Stillbirths	342	69.1

Formatted: Space After: 0 pt

Table 3: Fistula related characteristics patients admitted at Hamlin fistula center Addis Ababa Ethiopia 2023 (N=495).

Formatted Table

Variables	Category	Frequency	Perce
Age at development of fistula in years	≤18 age	57	11.5
	19-30 age	303	61.2
	≥31 age	135	27.3
Took prophylaxis's antibiotic	Yes	461	93.1
	No	34	6.9
Type of incontinency	Fecal	26	5.3
	Urinary	440	88.9
	Urinary Fecal	29	5.9
Duration of urinary ~~incontinency~~incontinence in days	Short duration (<30 days)	44	8.9
	Medium duration (30-365 days)	370	74.7
	Long duration (>365 days)	81	16.4
Got physiotherapy before surgery	Yes	12	2.4
	No	483	97.6
Got physiotherapy after surgery	Yes	36	7.3
	No	459	92.7
Got psychotherapy before surgery	Yes	200	40.4
	No	295	59.6
Got psychotherapy after surgery	Yes	93	18.8

	No	402	81.2
Got any surgery for the fistula previously at any fistula center	Yes<br>No	85<br>410	17.2<br>82.8
Frequency of previous surgery (n=85)	once<br>Twice<br>Three times	62<br>17<br>6	172.59%<br>203.4.0%<br>1.27.1%
**Type and characteristics of fistula**			
Vesico-vaginal fistula (VVF) (n=495)	No	64	12.9
	Yes	431	87.1
Length of vesico-vaginal fistula (n=431)	2.2-4cm	355	82.4
	≥5cm	76	17.6
VVF size based on distance from bladder (n= 431)	<5cm	21	4.9
	>5cm	410	95.1
Width of the VVF hole (n= 431)	0-2cm	2724	6354.9
	2.2-4cm	103	23.8
	≥5cm	56	12.9
Rectovaginal fistula (RVF) (n=495)	No	443	89.5
	Yes	52	10.5
Distance from hymen for RVF (n= 495)	No RVF types0-2cm	44332	89.557
	0-2cm	32	6.5
	3-5cm	169	33.93.
	>5cm	45	0.88.9
Iatrogenic fistula (n=495)	No	443	89.5
	Yes	52	10.5
Ureteric fistula (n= 495)	No	457	92.3
	Yes	38	7.7
Mixed fistula (n= 495)	No	368	74.3
	Yes	127	25.7
Status of urethra (n= 495)	Partially damaged<br>Not damaged<br>Complete urethral distraction	166<br>279<br>50	33.5<br>56.4<br>10.1
Stage of vaginal scaring (n= 495)	No vaginal scaring<br>Mild<br>Moderate<br>Severe	98<br>142<br>168<br>87	19.8<br>28.7<br>33.9<br>17.6
Fistula fibrosis(recto) (n= 495)	No<br>Mild<br>Moderate<br>Severe	443<br>18<br>18<br>16	89.5<br>3.6<br>3.6<br>3.2
Surgery Approach (n= 495)	Vaginal<br>Both<br>Abdominal	441<br>17<br>37	89.1<br>3.4<br>7.5
Duration of urethral catheterization after procedure in days (n= 495)	No<br>1-5<br>6-10	22<br>333<br>137	4.4<br>67.3<br>27.7

Formatted: Not Highlight

Formatted: Not Highlight

Formatted: Not Highlight

Formatted: Not Highlight

Formatted: Not Highlight

Formatted: Not Highlight

	>11	3	.6
Duration of day before treatment (n= 495)	≤1 year	348	70.3
	1-5 years	76	15.4
	>5 years	71	14.3
~~Out comes~~Outcomes (n= 495)	Censored	125	25.3
	Recovered	370	74.7

Formatted: Space After: 0 pt

Table 4: Comparisons of time to recovery among different levels of predictor variables using log-rank test from obstetric fistula patients admitted at Hamlin fistula center Addis Ababa Ethiopia 2023 (N= 495).

**Variables**	**Category**	**Frequency (%)**	**Median recovery time (weeks)**	**Log rank test**	
				X2	P Value
Occupation	Farmer	249(50.3)	6.9	6.2	.045
	House wifeHousewife	173(34.9)	5.6		
	governmental employee and others	73(14.7)	5.0		
Type of inconsistence	Urinary	440(88.9)	6.0	12.0	.002
	Fecal	26(5.3)	3.3		
	Both	29(5.9)	7.4		
Fecal Inconsistence	No	441(89.1)	5.7	7.0	.008
	Yes	54(10.9)	8.7		
Recto vaginal fistula	No	443(89.5)	5.7	6.0	.014
	Yes	52(10.5)	7.1		
Recto vaginal fibrosis (n= 52)	Mild	18(34.6)	4.0	11.1	.011
	Moderate	18(34.6)	10.1		
	Severe	16(30.8)	22.4		
Surgery approach	Vaginal	441(89.1)	5.9	6.5	.038
	Abdominal	37(7.5)	5.4		
	Both	17(3.4)	10.0		
Status of urethra	Not damaged	279(56.4)	5.4	6.0	.050
	Partially damaged	166(33.5)	6.0		
	Completely damaged	50(10.1)	8.0		
Stage of vaginal scarring	No vaginal scaring	98(19.8)	5.3	21.9	.000
	Mild	142(28.7)	5.6		
	Moderate	168(33.9)	5.1		
	Severe	87(17.6)	9.7		
Distance from hymen RFV (n= 52)	0-2cm	32(61.557.1)	8.7	11.3	.010
	3-5cm	169(30.833.9)	5.9		
	>5cm	45(7.99.0)	3.1		
VVF distance from bladder	<5cm	21(4.2)	8.7	4.1	.042

Formatted: Not Highlight
Formatted: Not Highlight
Formatted: Not Highlight
Formatted: Not Highlight
Formatted: Not Highlight
Commented [A1]: Not clear how this became 21 only.
Formatted: Highlight
Formatted: Font: (Default) Times New Roman
Formatted: Highlight
Formatted: Space After: 0 pt

Table 5: Bivariate and multivariable cox proportional hazard ratio indicating predictors of recovery time from obstetric fistula patients admitted at Hamlin fistula center Addis Ababa Ethiopia 2023 (N= 495).

Variable	Category	Survival status		CHR95%CI	P value	AHR95%CI	P value
		Recovered	Censored				
Marital status	Married	292	85	**1**		**1**	
	separated and oOthers**	78	40	.81(0.63,1.04)	0.096	.98(0.74,1.29)	.866
Educational status	No formal education	226	84	**1**		**1**	
	Had formal education	144	41	1.06(0.86,1.31)	0.574	0.92(0.72,1.17)	.490
Occupation of the women	Farmers	176	73	**1**		**1**	
	Housewife and oOthers***	194	52	1.15(0.94,1.41)	0.185	1.20(0.96,1.50)	.103
BMI	Normal weight	233	90	**1**		**1**	
	Abnormal weight	137	35	1.01(0.89,1.24)	.997	0.98(0.78,1.22)	.835
Place of deliveryDelivery	Health facility	316	95	1		1	
	Home delivery	54	30	.77(0.57,1.03)	.074	.90(0.65,1.24)	.513
Outcome of delivery	Alive	133	20	1		1	
	Stillbirths	237	105	.87(0.70,1.08)	.198	1.09(0.89,1.39)	.514
Age at development of fistula in years	<=18 age	35	22	1		1	
	19-30 age	222	81	1.11(0.78,1.59)	.569	.90(0.62,1.32)	.605
	>=31 age	113	22	1.41(0.96,2.06)	.077	1.09(0.72,1.65)	.680
Type of incontinency	Urinary	333	107	1			
	Both urinary and FfecalFecal and others***	37	18	1.28(0.91,1.80)	0.156	1.23(0.86,1.75)	2.60
Got physiotherapy after surgery	Yes	21	15	1.78(1.14,2.79)	.011	1.85(1.13,3.02)*	[illegible]
	No	349	110	1		1	
Got –psychotherapy before surgery	Yes	142	58	1		1	
	No	228	67	1.17(0.95,1.45)	.144	0.99(0.78,1.24)	.901
	No	62	31	1		1	

Got ~~psychotherapy~~ ~~after~~psychotherapy after surgery	Yes	308	94	1.32(1.00,1.75)	.051	1.15(0.86,1.55)	.3[illegible]
Length of fistula of VVF	No VVF~~2..2-4cm~~	11~~266~~	53~~89~~	1.44(0.98, 2.12)~~1.44(0.98,2.12)~~	0.06~~0~~ ~~.064~~	1.18(0.67, 2.06)~~5.23(1.17,12.33)*~~	~~0~~[illegible]
	2..2-4cm	266	89	1.17(0.87, 1.58)	0.29	5.23(1.17,12.33)*	0[illegible]
	>=5cm	51	25	1		1	
Width of fistula hole	No VVF~~0-2cm~~	51~~20911~~	11~~63~~	1.40(0.93, 2.14)~~1.41(0.93,2.14)~~	0.12~~.109~~	0.99(0.53, 1.86)~~.512(0.12,2.29)~~	0,[illegible] 3[illegible]
	0-2cm	211	63	1.22(0.86, 1.72)	0.24	.512(0.12,2.29)	0[illegible]
	2.2-4cm	69	34	1.09(0.74, 1.62)~~1.22(0.87,1.72)~~	0.65~~2~~ ~~49~~	1.01(0.53,1.92)	0[illegible] ~~0~~[illegible]
	>=5cm	39	17	1		1	
Rectovaginal fistula	No	339	104	1		1	
	Yes	31	21	.63(0.44,0.92	.016	.66(0.17,2.63)	.5[illegible]
Distance from hymen	No RVF ~~0-2cm~~	335~~20~~	104~~1~~ ~~2~~	1.43(0.85, 2.39)~~1.43(0.85,2.40)~~	0.181 ~~0.181~~	1.15(0.31, 4.23)~~.15(0.31,4.23)~~	0[illegible] 3[illegible]
	0-2cm	20	12	0.76(0.38, 1.51)	0.76	0.56(0.27, 1.16)	0[illegible]
	≥3cm	1~~55~~	~~99~~	1		1	
Status of urethra	Not damaged	226	53	1		1	
	Partially damaged	117	49	.93(0.74,1.16)	.527	0.99(0.78,1.28)	.[illegible]
	Complete urethral distraction	27	23	.61(0.41,0.91)	.016	.43(0.25,0.76)*	.0[illegible]
Stage of vaginal scarring	Mild(no) vaginal scaring	210	30	1		1	
	Moderate vaginal scarring	122	46	.91(0.72,1.13)	.390	.98(0.76,1.27)	.8[illegible]
	Severe vaginal scarring	38	49	.45(0.32,0.64)	.000	.60(0.40,0.89)*	.0[illegible]

Formatted: Font: 11 pt
Formatted: Font: 11 pt
Formatted: Font: 11 pt, Not Highlight
Formatted: Font: 11 pt, Not Highlight
Formatted: Font: 11 pt
Formatted: Font: 11 pt, Not Highlight
Formatted: Font: 11 pt
Formatted: Font: 11 pt, Not Highlight
Formatted: Font: 11 pt
Formatted: Font: 11 pt
Formatted: Font: 11 pt
Formatted: Font: 11 pt
Formatted: Font: 11 pt, Not Highlight
Formatted: Font: 11 pt
Formatted: Font: 11 pt, Not Highlight
Formatted: Font: 11 pt
Formatted: Font: 11 pt, Not Highlight
Formatted: Font: 11 pt
Formatted: Font: 11 pt, Not Highlight
Formatted: Font: 11 pt
Formatted: Font: 11 pt, Not Highlight
Formatted: Font: 11 pt
Formatted: Font: 11 pt
Formatted: Font: 11 pt
Formatted: Font: 11 pt
Formatted: Font: 11 pt, Not Highlight
Formatted: Font: 11 pt
Formatted: Font: 11 pt, Not Highlight
Formatted: Font: 11 pt
Formatted: Font: 11 pt, Not Highlight
Formatted: Font: 11 pt
Formatted: Font: 11 pt
Formatted: Font: 11 pt, Not Highlight
Formatted: Font: 11 pt
Formatted: Font: 11 pt, Not Highlight
Formatted: Font: 11 pt
Formatted: Font: 11 pt
Formatted: Font: 11 pt

Fistula	RVF not present	339	104	1		1	
fibrosis(recto)	RFV present	31	21	.63(0.44,0.92)	.016	0.61(0.40,0.83)*	**0.02**
Surgery Approach	Vaginal	331	110	1		1	
	Abdominal ~~and~~ or ~~other~~both****	39	15	1.03(0.74,1.44)	.854	.97(0.52,1.80)	.920

Keys: *P-value, < 0.05, **~~Single~~separated, divorced, widowed and single, ***Housewife, Self-employed, government-employee, and student~~, *** both urinary and fecal, **** both abdominal and vaginal~~

Formatted: Font: 11 pt

Formatted: Font: 11 pt

Formatted: Underline

Formatted: Space After: 0 pt

PGPH-D-25-02015

Time to recovery from Obstetric Fistula and its Predictors among Obstetric Fistula patients admitted to Hamlin Fistula Center, Addis Ababa, Ethiopia: A Survival Analysis.

PLOS Global Public Health.

To: Editor of PLOS Global Public Health

Dear Sir/Madam,

We wish to remind you of our submission of the manuscript entitled "*Time to recovery from Obstetric Fistula and its Predictors among Obstetric Fistula patients admitted to Hamlin Fistula Center, Addis Ababa, Ethiopia: A Survival Analysis.*" for publication consideration in PLOS Global Public Health. We express our gratitude for your efforts in reviewing and considering our article for further revision. In accordance with the feedback provided by the reviewers and editor, we have revised and modified the manuscript, addressing each comment individually as detailed below. The revised version, with tracked changes and clean version are attached for your reference. The responses to the reviewers' and editor's comments are outlined below.

Gizachew Abdissa Bulto

Email: gizachab@yahoo.com

Corresponding Author

Editor's Comment

PGPH-D-25-02015

Time to recovery from Obstetric Fistula and its Predictors among Obstetric Fistula patients admitted to Hamlin Fistula Center, Addis Ababa, Ethiopia: A Survival Analysis.

PLOS Global Public Health

Thank you for submitting your manuscript to PLOS Global Public Health. After careful consideration, we feel that it has merit but does not fully meet PLOS Global Public Health's publication criteria as it currently stands. Therefore, we invite you to submit a revised version of the manuscript that addresses the points raised during the review process.

Please carefully review the reviewers' comments and revise your manuscript accordingly, providing a point-by-point response to the reviewers upon resubmission.

Please include the following items when submitting your revised manuscript:

- A rebuttal letter that responds to each point raised by the editor and reviewer(s). You should upload this letter as a separate file labeled 'Response to Reviewers'.
- A marked-up copy of your manuscript that highlights changes made to the original version. You should upload this as a separate file labeled 'Revised Manuscript with Track Changes'.
- An unmarked version of your revised paper without tracked changes. You should upload this as a separate file labeled 'Manuscript'.

Author's response: *Thank you very for reviewing our paper and giving us your valuable comments. We are grateful for considering our article for publication. We did our revision accordingly and attached a rebuttal letter, Marked paper with tracked and highlighted document and clean version of the Manuscript.*

Journal Requirements:

If the reviewer comments include a recommendation to cite specific previously published works, please review and evaluate these publications to determine whether they are relevant and should be cited. There is no requirement to cite these works unless the editor has indicated otherwise.

Authors response: Thank you we have well noted.

Reviewers' comments:

Reviewer's Responses to Questions

Comments to the Author

1. Does this manuscript meet PLOS Global Public Health's publication criteria? Is the manuscript technically sound, and do the data support the conclusions? The manuscript must describe methodologically and ethically rigorous research with conclusions that are appropriately drawn based on the data presented.

Reviewer #1: Yes

Reviewer #2: Yes

Author's response: Thank you!

2. Has the statistical analysis been performed appropriately and rigorously?

Reviewer #1: Yes

Reviewer #2: No

Author's response: Thank you, we have checked and revised the results specifically for tables and text accordingly.

3. Have the authors made all data underlying the findings in their manuscript fully available (please refer to the Data Availability Statement at the start of the manuscript PDF file)?

The PLOS Data policy requires authors to make all data underlying the findings described in their manuscript fully available without restriction, with rare exception. The data should be provided as part of the manuscript or its supporting information, or deposited to a public repository. For example, in addition to summary statistics, the data points behind means, medians and variance measures should be available. If there are restrictions on publicly sharing data—e.g. participant privacy or use of data from a third party—those must be specified.

Reviewer #1: No

Reviewer #2: Yes

Author's response: We have included the statement about data availability and made all the data underlying the findings available as supporting information.

4. Is the manuscript presented in an intelligible fashion and written in standard English?

PLOS Global Public Health does not copyedit accepted manuscripts, so the language in

submitted articles must be clear, correct, and unambiguous. Any typographical or grammatical errors should be corrected at revision, so please note any specific errors here.

Reviewer #1: Yes

Reviewer #2: Yes

Author's response: Thank you, we are grateful for your time and forwarding us your valuable comments.

5. Review Comments to the Author

Please use the space provided to explain your answers to the questions above. You may also include additional comments for the author, including concerns about dual publication, research ethics, or publication ethics. (Please upload your review as an attachment if it exceeds 20,000 characters)

Reviewer #1:

First of all, I would like to sincerely thank the editor for inviting me to review the important paper titled " Time to recovery from Obstetric Fistula and its Predictors among Obstetric Fistula patients admitted to Hamlin Fistula Center, Addis Ababa, Ethiopia: A Survival Analysis." I also extend my appreciation to the author for providing such interesting and valuable evidence on an issue that is increasingly significant in developing countries like Ethiopia.

Author's response: Dear Reviewer, we thank you for taking your time and giving us your invaluable comments and suggestions.

Here is a suggested revision for your comments section:

General comments:

1. The paper is well written and organized, though some modifications are advised.

Author's response: Thank you!

2. From my search there are a lot of papers that say what is new for this paper in Ethiopia…

Author's response: Thank you! We added statements on the revised version to address this issue. The statement now reads:

> *"… Although fistula centers have been established in Ethiopia and the government has implemented strategic initiatives targeting risk reduction, identification and treatment, reintegration, and income generation for survivors, with the goal of eradicating OBF by 2025, the condition remains prevalent…*

.... Even though there are few studies on survival time of obstetric fistula conducted in the Northern and eastern Ethiopia, the recovery rate and median recovery time have not been well studied, and there is specifically no evidence from the country's first and largest Fistula center. Therefore, this study will assess the recovery rate and median recovery time and identify factors that predict the recovery time from OBF at Hamlin Fistula Center in Addis Ababa.....”

3. Attention is needed to improve sentence synthesis and grammar throughout the manuscript.

Author’s response: Dear reviewer, we have revised the whole section of the article and tried to also revise the sentences and grammatical issues as well. Thank you.

Specific Comments:

Abstract:

- Well written and organized, but please put the subtitles, like "methods," "result," and "conclusion and recommendation."

Author’s response: Thank you, we formatted the abstract according to the journal guideline. In authors guideline section it was stated that: “*While the Abstract is conceptually divided into three sections (Background, Methodology/Principal Findings, and Conclusions/Significance), do not apply these distinct headings to the Abstract within the article file*.”

- Please be specific about your recommendation.

Author’s response: Thank you. We have revised the conclusion and recommendation section. Now it reads:

“*Based on the findings, median recovery time from OBF was 5.9 weeks. Therefore, strengthening postoperative physiotherapy and providing specialized care for women with urethral injury or severe vaginal scarring could improve recovery time from OBF. These findings also highlighted the critical importance of individualized rehabilitation services in fistula treatment centers to optimize patient recovery and overall quality of life.*”

Introduction: Really, this section is also well written and organized. If possible, please include why this problem persists today, what has been done, what strategies are in place, what obstacles remain, and what the future implications for SGD are.

Author's response: Thank you for your suggestions, we have revised the introduction accordingly. We added statements on the 2nd and 4th paragraphs of the introduction.

"*… According to those reports, Ethiopia may not only fail to eliminate obstetric fistula by 2025 but also might be unable to achieve the Sustainable Development Goals of eradicating this condition by 2030…*"

"*… Although fistula centers have been established in Ethiopia and the government has implemented strategic initiatives targeting risk reduction, identification and treatment, reintegration, and income generation for survivors, with the goal of eradicating OBF by 2025, the condition remains prevalent….*

Even though there are few studies on survival time of obstetric fistula conducted in the Northern and eastern Ethiopia, the recovery rate and median recovery time have not been well studied, and there is specifically no evidence from the country's first and largest Fistula center. Therefore, this study will assess the recovery rate and median recovery time and identify factors that predict the recovery time from OBF at Hamlin Fistula Center in Addis Ababa …."

Methods: operational definition: Survival time: the time period from a starting treatment for obstetric fistula to recovery in weeks. Please, could you give the standard or duration follow-up?

Author's Response: Thank you. We have revised the operational definition accordingly. The revised version now read:

"*Survival time: the time period in weeks from the initiation of treatment for obstetric fistula to documented clinical recovery. Women were followed from the start of treatment until recovery.*"

⎫ Include the overall goodness-of-fit value of the model.

Author's response: Thank you very much. We have included the result in text under the result section.

> *"The Omnibus Test of Model Coefficients also revealed that the final Cox proportional hazards model provided a significantly better fit than the null model ($\chi^2 = 55.12$, $df = 25$, $p < 0.001$). This finding indicates that the predictors included in the model collectively explain a significant portion of the variation in recovery time among patients with obstetric fistula."*

⎫ Since the issue is sensitive, you need to provide the IRB number.

Author's response: Dear reviewer, thank you, we included the reference number from IRB under the Ethical approval section page 9.

Result: Well stated.

1. In such a study, it's advised to run the frequency both for the outcome and the censored with the present clearly.

Author's response: Dear reviewer, we thank you. We have included the frequencies for the predictors clearly within table 5 for both the outcome and censored the 3rd and 4th column.

Discussion: from your result "this result was lower than studies conducted in other parts of Ethiopia such as: at 299 Gondar University Teaching (88.07%) (20), Jimma University Medical Center (81.4%) (25) and 300 Mekele Hamlin Fistula Center (89.33%) " AA, the capital city where advanced medical procedures may probably be given… Please revise your justification.

Author's response: Thank you very much, it was a nice observation. We have accepted your comment and revised it accordingly. Now the revision read:

"The study aimed to determine the median recovery time and its influencing factors among patients with obstetric fistula. The findings indicated that obstetric fistula was healed in 74.7% (95%CI: 71.1, 78.2) of the patients, with the median duration of 5.86 weeks. The recovery rate is consistent with the study done in Gondar teaching hospital fistula center (73.7%) (18). However, this result was lower than studies conducted in other parts of Ethiopia, including another study at Gondar University Teaching and referral hospital (88.07%) (20), Jimma University Medical Center (81.4%) (25) and Mekele Hamlin Fistula Center (89.33%) (19). This variation may be attributed to the differences in patient's demographic and socio-economic characteristics, care seeking behavior and access to timely care across facilities. Although the Hamlin Fistula Center in Addis Ababa is situated in the capital, it receives complex and chronic cases referred from across the country, often involving extensive tissue damage or coexisting complications. These factors may contribute to the relatively lower recovery rate observed compared to other centers that serve less complicated cases."

⎫ *Please add implication after justification of all discussion points…*

Author's response: Thank you for your suggestions. We have now added the implication of the study after discussion. Now it read as:

> "*The findings of the current study underscore significant clinical implications for enhancing individualized rehabilitation services for patients with obstetric fistula, aiming to improve their recovery time and quality of life. Incorporating physiotherapy into the postoperative care of OBF patients and offering specialized care for women with urethral injuries or severe vaginal scarring can shorten the recovery time. Hospital administrators, health facilities, and healthcare providers must strengthen rehabilitation programs and adopt individualized women centered care for OBF patients*."

Reviewer #2:

Thank you for the opportunity to review this retrospective cohort study on recovery time and predictors of obstetric fistula at Hamlin Fistula Center. The study tackles a critical maternal health issue with a robust dataset and appropriate survival analysis, but several methodological, stylistic, and presentation aspects require refinement. With clearer methods, stronger narrative coherence, and a more human-centred framing, it has the potential to make a meaningful contribution to maternal health and provide valuable insights for clinicians, policymakers, and women's health advocates.

Author's response: Dear reviewer, we are grateful for your constructive comments and suggestions on our article. We have addressed those concerns in our revision. The responses were included in the sections below one by one. Thank you!

Section-by-Section Review

1. **Abstract**: There are inconsistent confidence interval (CI) formatting (commas vs. colons), fragmented presentation of results, and limited narrative flow. The abstract omits the human dimension and clinical implications of obstetric fistula (OBF).

a. Standardise statistical reporting for CIs.

b. Begin with a compelling sentence highlighting the human cost of OBF.

c. Present results in a coherent narrative rather than disjointed statistics.

d. Conclude with a statement on how findings could inform patient care, rehabilitation, or health policy.

Author's response: Dear reviewer, thank you very much for your constructive comments and suggestions. We have accepted your comment and revised it accordingly. We have revised the

whole abstract; background, methods, results and conclusion and recommendation sections. The whole revision we did could be seen from the documents with tracked changes.

2. **Introduction**: this section is informative but overly dense. Transition from global to local context is abrupt. The human experience of OBF pain, stigma, and isolation has been under emphasised.

a. Condense global statistics; shift focus towards Ethiopia's context.

b. Integrate a paragraph on the psychosocial toll of OBF to humanise the issue.

c. Where ethically permissible, include patient quotes or anecdotal reflections to ground the narrative in lived experience.

Author's response: Thank you very much for your constructive comments. We have revised making it briefer than before. On the first paragraph of our introduction, we have included the devastating effects of obstetric fistula on women which includes medical, psychological traumas, and socio-economic consequences. Also added women's quote from the study by Animut et al., (2019). The paragraph reads as:

"… Untreated, obstetric fistula leads to severe, lifetime morbidities with profound medical, psychological traumas and socio-economic consequences (3). It is one of the most neglected human rights and public health problems, causing tremendous challenges such as emotional distress, social isolation, family abandonment, and stigma. As reported by Animut et al., women with obstetric fistula experience profound social isolation, divorce, health problems, and psychological trauma. One woman expressed, "I am a person who is standing but not alive… however, I understand one thing darkness surrounds us despite the sun keeps rising" (crying). The majority (91%) of affected women experience stillbirths, while many also face anxiety, depression, discrimination, and worsening poverty. Common physical complications include nerve damage, foot drop, urinary and fecal incontinence, infertility, renal failure, and other pelvic disorders (4–8)…"

3. Methods: The Sample size calculation appears mismatched to the study design and there is no explanation of handling missing data or competing risks. Also ethical considerations have been presented procedurally, lacking empathy.

a. Clarify the rationale for sample size determination and any correction formula used.

Author's response: Thank you for the comment. The sample size was determined using OpenEpi, based on the assumptions described in the manuscript. The initial calculated sample size was 918.

However, the total number of women who underwent obstetric fistula surgery between January 1, 2017, and June 30, 2023, was only 945. Therefore, we applied a finite population correction formula to obtain a more precise estimate appropriate for our study population. After applying the correction and adding a 10% non-response, the final sample size was found to be 513.

We have included our revision in the document which now read:

"... Given that the study population available was limited to 945 individuals, which is below 10,000, we applied the finite population correction formula to adjust the sample size. By employing the standard correction formula, we calculated the sample size to be 466. Subsequently, after accounting for a 10% non-response rate, the final sample size was determined to be 513…."

b. Explain how missing data and competing risks were assessed and addressed.

Author's response: Thank you. We have included statements in the revised version. We have excluded those patients records with an incomplete record and data with significant missing values (18). Additionally, those woman who does not experience the event (recovery from obstetric fistula) before the study ends, a person who died after being admitted for obstetric fistula treatment and patients whose recovery status was unknown at discharge was censored in the current study.

We included a statement under the data processing and analysis section: "*The data cleaning was done to check for missing values and outliers. Those women with incomplete record and data with significant missing values were excluded from the study and we analyzed a complete case analysis*."

c. Reframe ethical considerations to emphasise patient dignity, confidentiality, and sensitivity, not just procedural approvals.

Author's response: Thank you for your constructive comment. We have added your comment on the revised version. Now it reads as:

"... As the current study relied on data collected from patient's record retrospectively, the requirement for the informed consent was waived by Ethical review board of College of Health Sciences and Referral Hospital, Ambo University. After submitting the proposal and explaining the study's purpose, benefits, and risks, permission and signed approval was also obtained from the Hamlin Fistula Hospital's administration. The study used existing patients' records or charts. Recognizing the sensitivity of the information, the trained data collectors took the patients' files to a separate room, where access was restricted to only the data collectors and the investigator. Once the data was collected, the records were placed back in their original place. We ensured patient

confidentiality strictly through removing personal identifiers such as their names, addresses, phone numbers, or the names of healthcare providers. Additionally, the confidentiality of the data was ensured by using anonymous questionnaires and storing the collected information in a password-protected folder where only researchers can get access to it."

4. **Results**: Some percentages and raw numbers are inconsistent, Survival curves lack Cis and interpretation of the Cox-Snell residual plots is vague.

a. Audit all numerical data for consistency.

Author's response: Thank you! We have checked all the data, percentages and tables for consistency and revised the numbers, percentages and tables accordingly (We did this for the whole result section).

b. Add CIs to survival curves.

c. Provide clearer interpretation of diagnostic plots and model assumptions.

Author's response: Thank you very much! We have added brief interpretation of the plot.

The revised statement now reads as:

"*Cox-Snell residuals were used to assess the goodness of fit of the model. The log logistic Cox-Snell residual plot appears to align closely with a 45-degree line passing through the reference line. This shows the observed line closely aligns with the reference line across the majority of the distribution, with only minor deviations at higher residual values. Therefore, the log-logistic model adequately fits the data, indicating that the model assumptions are reasonably met*"

5. **Discussion**: there is limited engagement with study limitations, superficial comparisons with other studies, and underdeveloped clinical implications.

a. Expand discussion of limitations, including retrospective bias, unmeasured confounders, and selection effects.

Author's response: Thank you very much. We have expanded the limitation section accordingly.

The revised version now read:

"As strengths the current study included data from randomly selected patients treated over seven years at a specialized fistula center and used survival analysis for time to recovery and its predictors, strengthening the reliability of findings. However, this study has several limitations. The study was based on retrospective design using secondary data, which limits accuracy and completeness of information from records. There is potential bias from incomplete documentation, data quality or inconsistent clinical recording practices across time. Furthermore, potential

predictors for obstetric fistula such as household wealth, sociocultural factors, nutritional status, distance from health facilities, and psychosocial supports were not collected in this study. The absence of these factors on patients' chart may have introduced unmeasured confounding, limiting ability to fully adjust for potential determinants influencing the time to recovery time from OBF. Additionally, since the study included only patients who received surgical treatment and had complete follow-up data, selection bias is possible, as women without care or incomplete records were excluded. This may affect generalizability of findings to all obstetric fistula patients in Ethiopia."

b. When comparing with other studies, critique methodological differences and their influence on findings.

Author's response: Thank you very much we did this throughout the discussion section from page 14 to 17.

c. Strengthen clinical implications: highlight how results may guide post-operative care, physiotherapy, counselling, and community reintegration.

Author's response: We thank you for your suggestions. Depending on the findings of the study, we included implications of the study in the revised version. The statement now reads as follows: "*The findings of the current study underscore significant clinical implications for enhancing individualized rehabilitation services for patients with obstetric fistula, aiming to improve their recovery time and quality of life. Incorporating physiotherapy into the postoperative care of OBF patients and offering specialized care for women with urethral injuries or severe vaginal scarring can shorten the recovery time. Hospital administrators, health facilities, and healthcare providers must strengthen rehabilitation programs and adopt individualized women centered care for OBF patients*."

6. **Tables and Figures**: there is inconsistent formatting of the tables and figures lack clarity and polish. There is also no flow diagram of patient selection.

a. Standardise table formatting, labelling, and style.

Author's response: Thank you very much! We did the formatting, labelling and style for the whole tables in the revised version. We checked and revised for the consistency in the numbers across the document and within tables as well.

b. Improve figure resolution and add clear, descriptive captions.

Author's response: Thank you, we improved the resolution and revised it accordingly.
c. Include a flowchart of patient inclusion/exclusion to enhance transparency.
Author's response: We appreciate your suggestion regarding the use of a flow chart for selecting study subjects. In this study, we chose study subjects through simple random sampling techniques after obtaining their medical registration numbers. Consequently, we decided not to include the flow diagram in the manuscript.

7**. Language and Style**: there are a lot and frequent grammatical errors, awkward phrasing, inconsistent terminology ("OBF" vs. "obstetric fistula") which makes readability very difficult.
a. Undertake thorough proofreading or professional language editing.
b. Maintain consistent terminology throughout.
c. Use active voice and empathetic language, particularly when describing patients' experiences.
Author's response: Thank you very much for your constructive comments and suggestions. We all authors did the proofreading and editing for language and also revised for consistency throughout the paper.

8. Human-Centred Enhancements:
a. Incorporate a brief patient vignette (with ethical clearance) to illustrate the journey from injury to recovery.
b. Discuss stigma, gendered vulnerabilities, and social reintegration, not just clinical outcomes.
c. Frame recovery as multidimensional—physical, psychological, and social.
Author's response: Thank you! This is a good observation. We included this especially in the background section of the paper.

Figure Resubmissions:

While revising your submission, we strongly recommend that you use PLOS's NAAS tool (https://ngplosjournals.pagemajik.ai/artanalysis) to test your figure files. NAAS can convert your figure files to the TIFF file type and meet basic requirements (such as print size, resolution), or provide you with a report on issues that do not meet our requirements and that NAAS cannot fix.

After uploading your figures to PLOS's NAAS tool - https://ngplosjournals.pagemajik.ai/artanalysis, NAAS will process the files provided and display the results in the "Uploaded Files" section of the page as the processing is complete. If the uploaded figures meet our requirements (or NAAS is able to fix the files to meet our requirements), the figure will be marked as "fixed" above. If NAAS is unable to fix the files, a red "failed" label will appear above. When NAAS has confirmed that the figure files meet our requirements, please download the file via the download option, and include these NAAS processed figure files when submitting your revised manuscript.

Thank you very we used this tool to improve our figures resolution and to adjust it to the journal standard.
