# Supplementary material for: Time to recovery from Obstetric Fistula and its predictors among patients admitted to Hamlin Fistula Center, Addis Ababa, Ethiopia: A survival analysis
Source: PLOS Glob Public Health. 2026 Jul 9;6(7):e0006848. doi: 10.1371/journal.pgph.0006848 (PMC13349304; doi:10.1371/journal.pgph.0006848)
Supplement: Attachment — Submitted filename: Time to Recovery from Obstetric Fistula.docx [file pgph.0006848.s004.docx]

# Time to Recovery from Obstetric Fistula

# Thank you for the opportunity to review this retrospective cohort study on recovery time and predictors of obstetric fistula at Hamlin Fistula Center. The study tackles a critical maternal health issue with a robust dataset and appropriate survival analysis, but several methodological, stylistic, and presentation aspects require refinement. With clearer methods, stronger narrative coherence, and a more human-centred framing, it has the potential to make a meaningful contribution to maternal health and provide valuable insights for clinicians, policymakers, and women’s health advocates.

## Section-by-Section Review

## 1. Abstract: **There are i**nconsistent confidence interval (CI) formatting (commas vs. colons), fragmented presentation of results, and limited narrative flow. The abstract omits the human dimension and clinical implications of obstetric fistula (OBF).

a. Standardise statistical reporting for CIs.
b. Begin with a compelling sentence highlighting the human cost of OBF.
c. Present results in a coherent narrative rather than disjointed statistics.
d. Conclude with a statement on how findings could inform patient care, rehabilitation, or health policy.

### 2. Introduction: this section is informative but overly dense. Transition from global to local context is abrupt. The human experience of OBF pain, stigma, and isolation has been underemphasised.

a. Condense global statistics; shift focus towards Ethiopia’s context.
b. Integrate a paragraph on the psychosocial toll of OBF to humanise the issue.
c. Where ethically permissible, include patient quotes or anecdotal reflections to ground the narrative in lived experience.

### 3. Methods: The Sample size calculation appears mismatched to the study design and there is no explanation of handling missing data or competing risks. Also ethical considerations have been are presented procedurally, lacking empathy.

a. Clarify the rationale for sample size determination and any correction formula used.
b. Explain how missing data and competing risks were assessed and addressed.
c. Reframe ethical considerations to emphasise patient dignity, confidentiality, and sensitivity, not just procedural approvals.

### 4. Results: Some percentages and raw numbers are inconsistent, Survival curves lack Cis and interpretation of the Cox-Snell residual plots is vague.

a. Audit all numerical data for consistency.
b. Add CIs to survival curves.
c. Provide clearer interpretation of diagnostic plots and model assumptions.

### 5. Discussion: there is limited engagement with study limitations, superficial comparisons with other studies, and underdeveloped clinical implications.

a. Expand discussion of limitations, including retrospective bias, unmeasured confounders, and selection effects.
b. When comparing with other studies, critique methodological differences and their influence on findings.
c. Strengthen clinical implications: highlight how results may guide post-operative care, physiotherapy, counselling, and community reintegration.

### 6. Tables and Figures: there is inconsistent formatting of the tables and figures lack clarity and polish. There is also no flow diagram of patient selection.

a. Standardise table formatting, labelling, and style.
b. Improve figure resolution and add clear, descriptive captions.
c. Include a flowchart of patient inclusion/exclusion to enhance transparency.

### 7. Language and Style: there are a lot and frequent grammatical errors, awkward phrasing, inconsistent terminology (“OBF” vs. “obstetric fistula”) which makes readability very difficult.

a. Undertake thorough proofreading or professional language editing.
b. Maintain consistent terminology throughout.
c. Use active voice and empathetic language, particularly when describing patients’ experiences.

### 8. Human-Centred Enhancements:

a. Incorporate a brief patient vignette (with ethical clearance) to illustrate the journey from injury to recovery.
b. Discuss stigma, gendered vulnerabilities, and social reintegration, not just clinical outcomes.
c. Frame recovery as multidimensional—physical, psychological, and social.

## Structural Format Upgrade

| **Section** | **Purpose** | **Enhancement** |
| --- | --- | --- |
| **Abstract** | Summarise key findings | Add emotional framing and clinical relevance |
| **Introduction** | Contextualise the issue | Humanise with lived experiences, balance global/local context |
| **Methods** | Describe study design | Clarify statistical rationale; reframe ethics with empathy |
| **Results** | Present findings | Ensure numerical consistency; add CIs; improve diagnostic clarity |
| **Discussion** | Interpret results | Deepen critique; expand clinical and psychosocial implications |
| **Conclusion** | Wrap up and suggest next steps | Emphasise actionable insights and patient-centred outcomes |
