# Supplementary material for: Time to recovery from Obstetric Fistula and its predictors among patients admitted to Hamlin Fistula Center, Addis Ababa, Ethiopia: A survival analysis
Source: PLOS Glob Public Health. 2026 Jul 9;6(7):e0006848. doi: 10.1371/journal.pgph.0006848 (PMC13349304; doi:10.1371/journal.pgph.0006848)
Supplement: Attachment — Submitted filename: V1 Response to Reviewers OBF time to recovery Nov 14, 2025.docx [file pgph.0006848.s008.docx]

**PGPH-D-25-02015**

**Time to recovery from Obstetric Fistula and its Predictors among Obstetric Fistula patients admitted to Hamlin Fistula Center, Addis Ababa, Ethiopia: A Survival Analysis.**

**PLOS Global Public Health.**

**To: Editor of PLOS Global Public Health**

**Dear Sir/Madam,**

We wish to remind you of our submission of the manuscript entitled “*Time to recovery from Obstetric Fistula and its Predictors among Obstetric Fistula patients admitted to Hamlin Fistula Center, Addis Ababa, Ethiopia: A Survival Analysis.*” for publication consideration in PLOS Global Public Health. We express our gratitude for your efforts in reviewing and considering our article for further revision. In accordance with the feedback provided by the reviewers and editor, we have revised and modified the manuscript, addressing each comment individually as detailed below. The revised version, with tracked changes and clean version are attached for your reference. The responses to the reviewers' and editor's comments are outlined below.

**Gizachew Abdissa Bulto**

**Email:** [**gizachab@yahoo.com**](mailto:gizachab@yahoo.com)

**Corresponding Author**

**Editor’s Comment**

PGPH-D-25-02015
Time to recovery from Obstetric Fistula and its Predictors among Obstetric Fistula patients admitted to Hamlin Fistula Center, Addis Ababa, Ethiopia: A Survival Analysis.
PLOS Global Public Health

Thank you for submitting your manuscript to PLOS Global Public Health. After careful consideration, we feel that it has merit but does not fully meet PLOS Global Public Health’s publication criteria as it currently stands. Therefore, we invite you to submit a revised version of the manuscript that addresses the points raised during the review process.

Please carefully review the reviewers' comments and revise your manuscript accordingly, providing a point-by-point response to the reviewers upon resubmission. 

Please include the following items when submitting your revised manuscript:

- A rebuttal letter that responds to each point raised by the editor and reviewer(s). You should upload this letter as a separate file labeled 'Response to Reviewers'.
- A marked-up copy of your manuscript that highlights changes made to the original version. You should upload this as a separate file labeled 'Revised Manuscript with Track Changes'.
- An unmarked version of your revised paper without tracked changes. You should upload this as a separate file labeled 'Manuscript'.

***Author’s response:*** *Thank you very for reviewing our paper and giving us your valuable comments. We are grateful for considering our article for publication. We did our revision accordingly and attached a rebuttal letter, Marked paper with tracked and highlighted document and clean version of the Manuscript.
Journal Requirements:*

If the reviewer comments include a recommendation to cite specific previously published works, please review and evaluate these publications to determine whether they are relevant and should be cited. There is no requirement to cite these works unless the editor has indicated otherwise. 
***Authors response:*** Thank you we have well noted.
**Reviewers' comments:
Reviewer's Responses to Questions**

**Comments to the Author**
1. Does this manuscript meet PLOS Global Public Health’s [publication criteria](https://track.editorialmanager.com/CL0/https:%2F%2Fjournals.plos.org%2Fglobalpublichealth%2Fs%2Fcriteria-for-publication/1/010f0199572c10cc-56b23034-53e2-4479-8d36-8bcfe77a0629-000000/fZK9IG55Un4Sy37p9L_-k026PomDZfAN72J8P-iVm0M=229)? Is the manuscript technically sound, and do the data support the conclusions? The manuscript must describe methodologically and ethically rigorous research with conclusions that are appropriately drawn based on the data presented.

Reviewer #1: Yes

Reviewer #2: Yes

***Author’s response:*** Thank you!

2. Has the statistical analysis been performed appropriately and rigorously?

Reviewer #1: Yes

Reviewer #2: No

***Author’s response:*** Thank you, we have checked and revised the results specifically for tables and text accordingly.

3. Have the authors made all data underlying the findings in their manuscript fully available (please refer to the Data Availability Statement at the start of the manuscript PDF file)?
The [PLOS Data policy](https://track.editorialmanager.com/CL0/https:%2F%2Fjournals.plos.org%2Fglobalpublichealth%2Fs%2Fdata-availability/1/010f0199572c10cc-56b23034-53e2-4479-8d36-8bcfe77a0629-000000/mdDxMQJ5CSYiB9gMjzw5QMsw9Uc0-PI3N0zXwTMCGmk=229) requires authors to make all data underlying the findings described in their manuscript fully available without restriction, with rare exception. The data should be provided as part of the manuscript or its supporting information, or deposited to a public repository. For example, in addition to summary statistics, the data points behind means, medians and variance measures should be available. If there are restrictions on publicly sharing data—e.g. participant privacy or use of data from a third party—those must be specified.

Reviewer #1: No

Reviewer #2: Yes

***Author’s response:*** We have included the statement about data availability and made all the data underlying the findings available as supporting information.

4. Is the manuscript presented in an intelligible fashion and written in standard English?

PLOS Global Public Health does not copyedit accepted manuscripts, so the language in submitted articles must be clear, correct, and unambiguous. Any typographical or grammatical errors should be corrected at revision, so please note any specific errors here.

Reviewer #1: Yes

Reviewer #2: Yes

***Author’s response:*** Thank you, we are grateful for your time and forwarding us your valuable comments.

**5. Review Comments to the Author**
Please use the space provided to explain your answers to the questions above. You may also include additional comments for the author, including concerns about dual publication, research ethics, or publication ethics. (Please upload your review as an attachment if it exceeds 20,000 characters)

**Reviewer #1:**

First of all, I would like to sincerely thank the editor for inviting me to review the important paper titled " Time to recovery from Obstetric Fistula and its Predictors among Obstetric Fistula patients admitted to Hamlin Fistula Center, Addis Ababa, Ethiopia: A Survival Analysis." I also extend my appreciation to the author for providing such interesting and valuable evidence on an issue that is increasingly significant in developing countries like Ethiopia.

**Author’s response**: Dear Reviewer, we thank you for taking your time and giving us your invaluable comments and suggestions.
Here is a suggested revision for your comments section:
General comments:
1. The paper is well written and organized, though some modifications are advised.

**Author’s response**: Thank you!
2. From my search there are a lot of papers that say what is new for this paper in Ethiopia…

**Author’s response**: Thank you! We added statements on the revised version to address this issue. The statement now reads:

*“… Although fistula centers have been established in Ethiopia and the government has implemented strategic initiatives targeting risk reduction, identification and treatment, reintegration, and income generation for survivors, with the goal of eradicating OBF by 2025, the condition remains prevalent…*

*.... Even though there are few studies on survival time of obstetric fistula conducted in the Northern and eastern Ethiopia, the recovery rate and median recovery time have not been well studied, and there is specifically no evidence from the country's first and largest Fistula center. Therefore, this study will assess the recovery rate and median recovery time and identify factors that predict the recovery time from OBF at Hamlin Fistula Center in Addis Ababa…..”*

3. Attention is needed to improve sentence synthesis and grammar throughout the manuscript.

**Author’s response**: Dear reviewer, we have revised the whole section of the article and tried to also revise the sentences and grammatical issues as well. Thank you.

**Specific Comments:
Abstract**:
⎫ Well written and organized, but please put the subtitles, like "methods," "result," and "conclusion and recommendation."

**Author’s response:** Thank you, we formatted the abstract according to the journal guideline. In authors guideline section it was stated that: “*While the Abstract is conceptually divided into three sections (Background, Methodology/Principal Findings, and Conclusions/Significance), do not apply these distinct headings to the Abstract within the article file*.”
⎫ Please be specific about your recommendation.

**Author’s response**: Thank you. We have revised the conclusion and recommendation section. Now it reads:

“*Based on the findings, median recovery time from OBF was 5.9 weeks. Therefore, strengthening postoperative physiotherapy and providing specialized care for women with urethral injury or severe vaginal scarring could improve recovery time from OBF. These findings also highlighted the critical importance of individualized rehabilitation services in fistula treatment centers to optimize patient recovery and overall quality of life.*”

**Introduction**: Really, this section is also well written and organized. If possible, please include why this problem persists today, what has been done, what strategies are in place, what obstacles remain, and what the future implications for SGD are.

**Author’s response**: Thank you for your suggestions, we have revised the introduction accordingly. We added statements on the 2^nd^ and 4^th^ paragraphs of the introduction.

“… *According to those reports, Ethiopia may not only fail to eliminate obstetric fistula by 2025 but also might be unable to achieve the Sustainable Development Goals of eradicating this condition by 2030*…”

“… *Although fistula centers have been established in Ethiopia and the government has implemented strategic initiatives targeting risk reduction, identification and treatment, reintegration, and income generation for survivors, with the goal of eradicating OBF by 2025, the condition remains prevalent….*

*Even though there are few studies on survival time of obstetric fistula conducted in the Northern and eastern Ethiopia, the recovery rate and median recovery time have not been well studied, and there is specifically no evidence from the country's first and largest Fistula center. Therefore, this study will assess the recovery rate and median recovery time and identify factors that predict the recovery time from OBF at Hamlin Fistula Center in Addis Ababa* ….”

**Methods**: operational definition: Survival time: the time period from a starting treatment for obstetric fistula to recovery in weeks. Please, could you give the standard or duration follow-up?

**Author’s Response**: Thank you. We have revised the operational definition accordingly. The revised version now read:

“*Survival time: the time period in weeks from the initiation of treatment for obstetric fistula to documented clinical recovery. Women were followed from the start of treatment until recovery.*”

⎫ Include the overall goodness-of-fit value of the model.

**Author’s response**: Thank you very much. We have included the result in text under the result section.

*“The Omnibus Test of Model Coefficients also revealed that the final Cox proportional hazards model provided a significantly better fit than the null model (χ² = 55.12, df = 25, p < 0.001). This finding indicates that the predictors included in the model collectively explain a significant portion of the variation in recovery time among patients with obstetric fistula.”*

⎫ Since the issue is sensitive, you need to provide the IRB number.

**Author’s response**: Dear reviewer, thank you, we included the reference number from IRB under the Ethical approval section page 9.
**Result**: Well stated.
1. In such a study, it's advised to run the frequency both for the outcome and the censored with the present clearly.

**Author’s response**: Dear reviewer, we thank you. We have included the frequencies for the predictors clearly within table 5 for both the outcome and censored the 3^rd^ and 4^th^ column.

**Discussion**: from your result "this result was lower than studies conducted in other parts of Ethiopia such as: at 299 Gondar University Teaching (88.07%) (20), Jimma University Medical Center (81.4%) (25) and 300 Mekele Hamlin Fistula Center (89.33%) " AA, the capital city where advanced medical procedures may probably be given… Please revise your justification.

**Author’s response**: Thank you very much, it was a nice observation. We have accepted your comment and revised it accordingly. Now the revision read:

*“The study aimed to determine the median recovery time and its influencing factors among patients with obstetric fistula. The findings indicated that obstetric fistula was healed in 74.7% (95%CI: 71.1, 78.2) of the patients, with the median duration of 5.86 weeks. The recovery rate is consistent with the study done in Gondar teaching hospital fistula center (73.7%) (18). However, this result was lower than studies conducted in other parts of Ethiopia, including another study at Gondar University Teaching and referral hospital (88.07%) (20), Jimma University Medical Center (81.4%) (25) and Mekele Hamlin Fistula Center (89.33%) (19). This variation may be attributed to the differences in patient’s demographic and socio-economic characteristics, care seeking behavior and access to timely care across facilities. Although the Hamlin Fistula Center in Addis Ababa is situated in the capital, it receives complex and chronic cases referred from across the country, often involving extensive tissue damage or coexisting complications. These factors may contribute to the relatively lower recovery rate observed compared to other centers that serve less complicated cases.”*

*⎫ Please add implication after justification of all discussion points…*

**Author’s response**: Thank you for your suggestions. We have now added the implication of the study after discussion. Now it read as:

“*The findings of the current study underscore significant clinical implications for enhancing individualized rehabilitation services for patients with obstetric fistula, aiming to improve their recovery time and quality of life. Incorporating physiotherapy into the postoperative care of OBF patients and offering specialized care for women with urethral injuries or severe vaginal scarring can shorten the recovery time. Hospital administrators, health facilities, and healthcare providers must strengthen rehabilitation programs and adopt individualized women centered care for OBF patients*.”

**Reviewer #2:**

Thank you for the opportunity to review this retrospective cohort study on recovery time and predictors of obstetric fistula at Hamlin Fistula Center. The study tackles a critical maternal health issue with a robust dataset and appropriate survival analysis, but several methodological, stylistic, and presentation aspects require refinement. With clearer methods, stronger narrative coherence, and a more human-centred framing, it has the potential to make a meaningful contribution to maternal health and provide valuable insights for clinicians, policymakers, and women’s health advocates.

**Author’s response**: Dear reviewer, we are grateful for your constructive comments and suggestions on our article. We have addressed those concerns in our revision. The responses were included in the sections below one by one. Thank you!
**Section-by-Section Review**
1. **Abstract**: There are inconsistent confidence interval (CI) formatting (commas vs. colons), fragmented presentation of results, and limited narrative flow. The abstract omits the human dimension and clinical implications of obstetric fistula (OBF).
a. Standardise statistical reporting for CIs.
b. Begin with a compelling sentence highlighting the human cost of OBF.
c. Present results in a coherent narrative rather than disjointed statistics.
d. Conclude with a statement on how findings could inform patient care, rehabilitation, or health policy.

**Author’s response**: Dear reviewer, thank you very much for your constructive comments and suggestions. We have accepted your comment and revised it accordingly. We have revised the whole abstract; background, methods, results and conclusion and recommendation sections. The whole revision we did could be seen from the documents with tracked changes.
2. **Introduction**: this section is informative but overly dense. Transition from global to local context is abrupt. The human experience of OBF pain, stigma, and isolation has been under emphasised.
a. Condense global statistics; shift focus towards Ethiopia’s context.
b. Integrate a paragraph on the psychosocial toll of OBF to humanise the issue.
c. Where ethically permissible, include patient quotes or anecdotal reflections to ground the narrative in lived experience.

**Author’s response**: Thank you very much for your constructive comments. We have revised making it briefer than before. On the first paragraph of our introduction, we have included the devastating effects of obstetric fistula on women which includes medical, psychological traumas, and socio-economic consequences. Also added women’s quote from the study by Animut et al., (2019). The paragraph reads as:

*“… Untreated, obstetric fistula leads to severe, lifetime morbidities with profound medical, psychological traumas and socio-economic consequences (3). It is one of the most neglected human rights and public health problems, causing tremendous challenges such as emotional distress, social isolation, family abandonment, and stigma. As reported by Animut et al., women with obstetric fistula experience profound social isolation, divorce, health problems, and psychological trauma. One woman expressed, “I am a person who is standing but not alive… however, I understand one thing darkness surrounds us despite the sun keeps rising” (crying). The majority (91%) of affected women experience stillbirths, while many also face anxiety, depression, discrimination, and worsening poverty. Common physical complications include nerve damage, foot drop, urinary and fecal incontinence, infertility, renal failure, and other pelvic disorders (4–8)…”*

**3. Methods**: The Sample size calculation appears mismatched to the study design and there is no explanation of handling missing data or competing risks. Also ethical considerations have been presented procedurally, lacking empathy.

a. Clarify the rationale for sample size determination and any correction formula used.

**Author’s response**: Thank you for the comment. The sample size was determined using OpenEpi, based on the assumptions described in the manuscript. The initial calculated sample size was 918. However, the total number of women who underwent obstetric fistula surgery between January 1, 2017, and June 30, 2023, was only 945. Therefore, we applied a finite population correction formula to obtain a more precise estimate appropriate for our study population. After applying the correction and adding a 10% non-response, the final sample size was found to be 513.

We have included our revision in the document which now read:

*“… Given that the study population available was limited to 945 individuals, which is below 10,000, we applied the finite population correction formula to adjust the sample size. By employing the standard correction formula, we calculated the sample size to be 466. Subsequently, after accounting for a 10% non-response rate, the final sample size was determined to be 513….”*

b. Explain how missing data and competing risks were assessed and addressed.

**Author’s response**: Thank you. We have included statements in the revised version. We have excluded those patients records with an incomplete record and data with significant missing values (18). Additionally, those woman who does not experience the event (recovery from obstetric fistula) before the study ends, a person who died after being admitted for obstetric fistula treatment and patients whose recovery status was unknown at discharge was censored in the current study.

We included a statement under the data processing and analysis section: “*The data cleaning was done to check for missing values and outliers. Those women with incomplete record and data with significant missing values were excluded from the study and we analyzed a complete case analysis*.”

c. Reframe ethical considerations to emphasise patient dignity, confidentiality, and sensitivity, not just procedural approvals.

**Author’s response**: Thank you for your constructive comment. We have added your comment on the revised version. Now it reads as:

*“… As the current study relied on data collected from patient’s record retrospectively, the requirement for the informed consent was waived by Ethical review board of College of Health Sciences and Referral Hospital, Ambo University. After submitting the proposal and explaining the study's purpose, benefits, and risks, permission and signed approval was also obtained from the Hamlin Fistula Hospital’s administration. The study used existing patients’ records or charts. Recognizing the sensitivity of the information, the trained data collectors took the patients' files to a separate room, where access was restricted to only the data collectors and the investigator. Once the data was collected, the records were placed back in their original place. We ensured patient confidentiality strictly through removing personal identifiers such as their names, addresses, phone numbers, or the names of healthcare providers. Additionally, the confidentiality of the data was ensured by using anonymous questionnaires and storing the collected information in a password-protected folder where only researchers can get access to it.”*

4. **Results**: Some percentages and raw numbers are inconsistent, Survival curves lack Cis and interpretation of the Cox-Snell residual plots is vague.
a. Audit all numerical data for consistency.

**Author’s response**: Thank you! We have checked all the data, percentages and tables for consistency and revised the numbers, percentages and tables accordingly (We did this for the whole result section).
b. Add CIs to survival curves.

c. Provide clearer interpretation of diagnostic plots and model assumptions.

**Author’s response**: Thank you very much! We have added brief interpretation of the plot.

The revised statement now reads as:

“*Cox-Snell residuals were used to assess the goodness of fit of the model. The log logistic Cox-Snell residual plot appears to align closely with a 45-degree line passing through the reference line. This shows the observed line closely aligns with the reference line across the majority of the distribution, with only minor deviations at higher residual values. Therefore, the log-logistic model adequately fits the data, indicating that the model assumptions are reasonably met*”
5. **Discussion**: there is limited engagement with study limitations, superficial comparisons with other studies, and underdeveloped clinical implications.
a. Expand discussion of limitations, including retrospective bias, unmeasured confounders, and selection effects.

**Author’s response**: Thank you very much. We have expanded the limitation section accordingly. The revised version now read:

*“As strengths the current study included data from randomly selected patients treated over seven years at a specialized fistula center and used survival analysis for time to recovery and its predictors, strengthening the reliability of findings. However, this study has several limitations. The study was based on retrospective design using secondary data, which limits accuracy and completeness of information from records. There is potential bias from incomplete documentation, data quality or inconsistent clinical recording practices across time. Furthermore, potential predictors for obstetric fistula such as household wealth, sociocultural factors, nutritional status, distance from health facilities, and psychosocial supports were not collected in this study. The absence of these factors on patients’ chart may have introduced unmeasured confounding, limiting ability to fully adjust for potential determinants influencing the time to recovery time from OBF. Additionally, since the study included only patients who received surgical treatment and had complete follow-up data, selection bias is possible, as women without care or incomplete records were excluded. This may affect generalizability of findings to all obstetric fistula patients in Ethiopia.”*

b. When comparing with other studies, critique methodological differences and their influence on findings.

**Author’s response:** Thank you very much we did this throughout the discussion section from page 14 to 17.

c. Strengthen clinical implications: highlight how results may guide post-operative care, physiotherapy, counselling, and community reintegration.

**Author’s response:** We thank you for your suggestions. Depending on the findings of the study, we included implications of the study in the revised version. The statement now reads as follows:

“*The findings of the current study underscore significant clinical implications for enhancing individualized rehabilitation services for patients with obstetric fistula, aiming to improve their recovery time and quality of life. Incorporating physiotherapy into the postoperative care of OBF patients and offering specialized care for women with urethral injuries or severe vaginal scarring can shorten the recovery time. Hospital administrators, health facilities, and healthcare providers must strengthen rehabilitation programs and adopt individualized women centered care for OBF patients*.”

6. **Tables and Figures**: there is inconsistent formatting of the tables and figures lack clarity and polish. There is also no flow diagram of patient selection.
a. Standardise table formatting, labelling, and style.

**Author’s response**: Thank you very much! We did the formatting, labelling and style for the whole tables in the revised version. We checked and revised for the consistency in the numbers across the document and within tables as well.
b. Improve figure resolution and add clear, descriptive captions.

Author’s response: Thank you, we improved the resolution and revised it accordingly.
c. Include a flowchart of patient inclusion/exclusion to enhance transparency.

**Author’s response**: We appreciate your suggestion regarding the use of a flow chart for selecting study subjects. In this study, we chose study subjects through simple random sampling techniques after obtaining their medical registration numbers. Consequently, we decided not to include the flow diagram in the manuscript.

7**. Language and Style**: there are a lot and frequent grammatical errors, awkward phrasing, inconsistent terminology (“OBF” vs. “obstetric fistula”) which makes readability very difficult.
a. Undertake thorough proofreading or professional language editing.
b. Maintain consistent terminology throughout.
c. Use active voice and empathetic language, particularly when describing patients’ experiences.

**Author’s response**: Thank you very much for your constructive comments and suggestions. We all authors did the proofreading and editing for language and also revised for consistency throughout the paper.

**8. Human-Centred Enhancements:**
a. Incorporate a brief patient vignette (with ethical clearance) to illustrate the journey from injury to recovery.
b. Discuss stigma, gendered vulnerabilities, and social reintegration, not just clinical outcomes.
c. Frame recovery as multidimensional—physical, psychological, and social.

Author’s response: Thank you! This is a good observation. We included this especially in the background section of the paper.

Figure Resubmissions:

While revising your submission, we strongly recommend that you use PLOS’s NAAS tool (https://ngplosjournals.pagemajik.ai/artanalysis) to test your figure files. NAAS can convert your figure files to the TIFF file type and meet basic requirements (such as print size, resolution), or provide you with a report on issues that do not meet our requirements and that NAAS cannot fix.

After uploading your figures to PLOS’s NAAS tool - https://ngplosjournals.pagemajik.ai/artanalysis, NAAS will process the files provided and display the results in the "Uploaded Files" section of the page as the processing is complete. If the uploaded figures meet our requirements (or NAAS is able to fix the files to meet our requirements), the figure will be marked as "fixed" above. If NAAS is unable to fix the files, a red "failed" label will appear above. When NAAS has confirmed that the figure files meet our requirements, please download the file via the download option, and include these NAAS processed figure files when submitting your revised manuscript.

Thank you very we used this tool to improve our figures resolution and to adjust it to the journal standard.
