# Supplementary material for: Time to recovery from Obstetric Fistula and its predictors among patients admitted to Hamlin Fistula Center, Addis Ababa, Ethiopia: A survival analysis
Source: PLOS Glob Public Health. 2026 Jul 9;6(7):e0006848. doi: 10.1371/journal.pgph.0006848 (PMC13349304; doi:10.1371/journal.pgph.0006848)
Supplement: Attachment — Submitted filename: Dr Nkhatas report.docx [file pgph.0006848.s006.docx]

Thank you for your manuscript examining recovery time from obstetric fistula at Hamlin Fistula Center. While the research question is valuable and the methodology generally sound, there are still **critical errors and incomplete reporting in the current form**.

**1. Mathematical Errors in Tables:** Table 3, line 531 shows "once: 62 (172.59%)" - impossible percentage. Recalculate ALL percentages in ALL tables, Correct: 62/85 = 72.9%, not 172.59%, Verify every calculation with independent audit

**2. Missing Confidence Intervals on Figures:** Figures 1-3 lack 95% CI bands on Kaplan-Meier curves. Regenerate all K-M curves with 95% CI shaded regions, Update legends: "Figure 1: Kaplan-Meier survival curve (n=495). Shaded area = 95% CI. Median recovery: 5.9 weeks (95% CI: 5.1–6.6)."

**3. Data Inconsistencies:** Numbers don't match between text, tables, and abstract. Create master verification showing: Total enrolled: 513, Analyzed: 495 (96.5%), Excluded: 18 (state reasons), Recovered: 370 (74.7%), Censored: 125 (25.3%), Median recovery: 5.9 weeks (95% CI: 5.1–6.6). Cross-check every mention of these numbers.

**4. Sample Size Calculation:** Jump from 918→513 unexplained. Add to Methods: *"Initial sample size: 918 (OpenEpi, 1:1 ratio, 95% CI, 80% power, HR=1.59). Since available population was 945 (N<10,000), we applied finite population correction: n_adjusted = (918×945)/(918+945-1) = 466. Adding 10% for incomplete records: final n=513."* Explain why 10% for retrospective review.

**5. Table 5 Verification:** Recovered + Censored ≠ expected totals in multiple rows. Verify EVERY row: Recovered + Censored = Total, Example: Married: 292+85=377 ✓, Fix "Distance from hymen" totals

**6. Patient Selection Flowchart**

Create Figure 0 (CONSORT-style):

Total OBF patients (n=945)

↓

Random sampling (n=513)

↓

Excluded incomplete records (n=18)

• Missing admission date: n=?

• Missing discharge date: n=?

• Missing outcome: n=?

↓

Final sample (n=495)

↓

├─ Recovered (n=370, 74.7%)

├─ Censored-alive (n=?)

├─ Censored-unknown (n=?)

└─ Died (n=?)

**7. Selection Bias Analysis:** Create **Supplementary Table S1** and Add to Discussion: "Comparison revealed [no differences/differences in X], suggesting [minimal/potential] selection bias."

| **Characteristic** | **Included (n=495)** | **Excluded (n=18)** | **p-value** |
| --- | --- | --- | --- |
| Age (mean±SD) |  |  |  |
| VVF (%) |  |  |  |
| Urethral damage (%) |  |  |  |
| Severe scarring (%) |  |  |  |

**8. Statistical Reporting Gaps**

**A. Proportional Hazards Testing** Add to Results: "Proportional hazards assumption verified using log-minus-log plots and Schoenfeld residuals. All variables satisfied assumption (p>0.05) except [list violations]. Violating variables were [handled how]."

**B. Model Goodness-of-Fit** Add to Results: "Final Cox model showed better fit than null model (Omnibus: χ²=55.12, df=25, p<0.001). Cox-Snell residuals confirmed adequate fit (Figure 4), with observed values following 45° reference line. Minor deviations at residuals >4 reflect fewer long-term observations but don't invalidate model."

**C. Subgroup Sizes in Table 5** Add column showing n for each category, especially for wide CIs (fistula length AHR=5.23, CI: 1.17–12.33).

**9. Mortality Data**

**Problem:** Deaths buried in "censored" without details. Report: "Of 125 censored, XXX died (XX%), XXX unknown status, XXX incomplete recovery." Split Table 3 "Outcomes": Recovered | Censored-alive | Died Add to Limitations: "Death treated as censoring. If mortality differs by predictors, hazard ratios may be biased. However, death rate was low (XX%). Future studies should use competing risk models."

**10. Context for Lower Recovery Rate:** 74.7% recovery vs. 81–89% at other centers, inadequately explained. **Add to Methods:** "Hamlin serves as Ethiopia's national referral center, receiving complex cases including failed repairs, extensive tissue damage, and prolonged fistula duration from all regions." **Expand in Discussion:** "Our 74.7% rate is lower than Gondar (88.07%), Jimma (81.4%), and Mekelle (89.33%). This reflects Hamlin's referral center role. Our cohort included: 17.2% with prior failed surgery, 10.1% complete urethral destruction, 16.4% with fistula >1 year. Median recovery time (5.9 weeks) matches other centers (5.14–5.19 weeks), suggesting comparable surgical quality. Lower overall rate reflects case-mix, not inferior care."

**Add Limitation:** "As tertiary center, findings may not generalize to primary-level repairs."

**11. Clinical Implications; Current:** Too generic: be Specific, actionable e.g

"**Implications for optimizing OBF care:**

**1. Scale physiotherapy:** Only 7.3% received physiotherapy despite 85% faster recovery (AHR=1.85). Scaling to all patients could improve recovery from 74.7% to [estimate]. Address barriers through: training staff, low-cost alternatives (self-administered exercises), community health worker support.

**2. Counsel complex cases:** Women with urethral destruction (10.1%) recover 57% slower. Set realistic expectations (8 vs. 5.4 weeks) and organize support groups.

**3. Triage resources:** Identify high-risk patients (severe scarring, 17.6%) at admission for prioritized physiotherapy, extended hospitalization, nutritional support.

**4. Research priorities:** Confirm fistula size effect (AHR=5.23, wide CI) in larger studies. Develop standardized severity classification.

**5. Policy shift:** Ethiopia's 2030 OBF elimination requires shifting from surgical access to post-operative care quality. <8% receiving evidence-based physiotherapy shows critical gap."

**12. Expand Limitations:** Add

- **Retrospective:** Missing confounders (nutrition, wealth, distance, surgeon experience, year)
- **Temporal:** 2017–2023 protocols may have evolved
- **Single-center:** Tertiary referral, may not generalize
- **Recovery definition:** Measured continence only, not psychological/social dimensions
- **Excluded cases:** 18 (3.5%) may represent most complex

**13. Improve Abstract**

**Restructure as:**

**Background:** Start with human impact, state knowledge gap, study aim.

**Methods:** Concise study design, analysis approach (K-M, Cox regression).

**Results:** Lead with recovery rate/time, then key predictors. **Highlight physiotherapy finding upfront.**

**Conclusions:** Specific recommendations (scale physiotherapy, counsel complex cases, individualized rehabilitation).

**14. Comparison Table**

Create **Supplementary Table S2** comparing all Ethiopian OBF studies:

| **Study** | **Location** | **N** | **Recovery Rate** | **Median Time** | **Notes** |
| --- | --- | --- | --- | --- | --- |
| Current | Hamlin | 495 | 74.7% | 5.9 wk | Tertiary referral |
| Derso 2020 | Gondar |  | 73.7% | 5.19 wk |  |
| Yismaw | Gondar U |  | 88.07% | 5.14 wk |  |
| Hussen 2017 | Harar |  |  | 2.67 wk | Mean, not median |
| Bihon 2022 | Mekelle |  | 89.33% |  |  |

Reference in Discussion.

**QUESTIONS TO ANSWER**

1. How many died of the 125 censored?
2. Why were 18 records incomplete? (breakdown by reason)
3. Clinical criteria for "recovery" at Hamlin?
4. Did surgical protocols change 2017–2023?
5. Year-stratified recovery rates?
6. Current physiotherapy rate (2024–2025)?
7. Prior surgeries at Hamlin vs. elsewhere (of 85)?
8. Findings shared with Hamlin administration? Response?
