# Supplementary material for: Time to recovery from Obstetric Fistula and its predictors among patients admitted to Hamlin Fistula Center, Addis Ababa, Ethiopia: A survival analysis
Source: PLOS Glob Public Health. 2026 Jul 9;6(7):e0006848. doi: 10.1371/journal.pgph.0006848 (PMC13349304; doi:10.1371/journal.pgph.0006848)
Supplement: Attachment — Submitted filename: V2 Response to Reviewers Comment.docx [file pgph.0006848.s010.docx]

**Time to recovery from Obstetric Fistula and its Predictors among Obstetric Fistula patients admitted to Hamlin Fistula Center, Addis Ababa, Ethiopia: A Survival Analysis**

**PGPH-D-25-02015R1**

**To: PLOS Global Public Health**

**Dear Dr Rahul Gajbhiye,**

**(Academic Editor PLOS Global Public Health)**

We sincerely express our gratitude for your efforts in reviewing our article and forwarding your invaluable comments and suggestions. We are also excited for considering our article further for publication. In accordance with the feedback provided by the reviewers and editor, we have revised and modified the manuscript, addressing each comment individually as detailed below. The revised version, with tracked changes and clean manuscript version are attached for your reference. The responses to the reviewers' and editor comments are outlined below.

Thank you very much.

Sincerely,

Gizachew Abdissa Bulto

Email: [gizachab@yahoo.com](mailto:gizachab@yahoo.com)

Corresponding author

**Reviewers’ and Editor Comments**

**Comments to the Author**
1. If the authors have adequately addressed your comments raised in a previous round of review and you feel that this manuscript is now acceptable for publication, you may indicate that here to bypass the “Comments to the Author” section, enter your conflict of interest statement in the “Confidential to Editor” section, and submit your "Accept" recommendation.

Reviewer #2: (No Response)

Reviewer #3: (No Response)

2. Does this manuscript meet PLOS Global Public Health’s [publication criteria](https://journals.plos.org/globalpublichealth/s/criteria-for-publication)? Is the manuscript technically sound, and do the data support the conclusions? The manuscript must describe methodologically and ethically rigorous research with conclusions that are appropriately drawn based on the data presented.

Reviewer #2: Yes

Reviewer #3: Yes

**Author’s response**: Thank you very much.

3. Has the statistical analysis been performed appropriately and rigorously?

Reviewer #2: No

Reviewer #3: Yes

**Author’s response**: Thank you very much. We have improved it through clarifying those concerns and adding details.

4. Have the authors made all data underlying the findings in their manuscript fully available (please refer to the Data Availability Statement at the start of the manuscript PDF file)?

The [PLOS Data policy](https://journals.plos.org/globalpublichealth/s/data-availability) requires authors to make all data underlying the findings described in their manuscript fully available without restriction, with rare exception. The data should be provided as part of the manuscript or its supporting information, or deposited to a public repository. For example, in addition to summary statistics, the data points behind means, medians and variance measures should be available. If there are restrictions on publicly sharing data—e.g. participant privacy or use of data from a third party—those must be specified.

Reviewer #2: Yes

Reviewer #3: Yes

**Author’s response**: Thank you.

5. Is the manuscript presented in an intelligible fashion and written in standard English?

PLOS Global Public Health does not copyedit accepted manuscripts, so the language in submitted articles must be clear, correct, and unambiguous. Any typographical or grammatical errors should be corrected at revision, so please note any specific errors here.

Reviewer #2: Yes

Reviewer #3: Yes

**Author’s response**: Thank you.

6. Review Comments to the Author

Please use the space provided to explain your answers to the questions above. You may also include additional comments for the author, including concerns about dual publication, research ethics, or publication ethics. (Please upload your review as an attachment if it exceeds 20,000 characters)

Reviewer #2: Refer to attached file

Reviewer #3: manuscript is suitable for publication with minor changes. comment is inserted in attached pdf file

**Author’s response**: Thank you, we are grateful for all your comments and suggestions.

**Reviewer 2**

Thank you for your manuscript examining recovery time from obstetric fistula at Hamlin Fistula Center. While the research question is valuable and the methodology generally sound, there are still **critical errors and incomplete reporting in the current form**.

**1. Mathematical Errors in Tables:** Table 3, line 531 shows "once: 62 (172.59%)" - impossible percentage. Recalculate ALL percentages in ALL tables, Correct: 62/85 = 72.9%, not 172.59%, Verify every calculation with independent audit

**Authors’ Response**: Thank you so much for your valuable comments. We have checked all the tables and cells for correct numbers and percentages and checked for the consistency. We have modified table 3 and Table 4 accordingly. The error happened as we calculated the proportion of all 495 participants. Now we corrected it in the revised version.

**2. Missing Confidence Intervals on Figures:** Figures 1-3 lack 95% CI bands on Kaplan-Meier curves. Regenerate all K-M curves with 95% CI shaded regions, Update legends: "Figure 1: Kaplan-Meier survival curve (n=495). Shaded area = 95% CI. Median recovery: 5.9 weeks (95% CI: 5.1–6.6)."

**Authors’ response**: Thank you very much for your valuable suggestions! We accepted your comment and updated the figures accordingly. We have added the 95% confidence interval with shaded part and revised the legends accordingly for figures 1, 2 and 3.

**3. Data Inconsistencies:** Numbers don't match between text, tables, and abstract. Create master verification showing: Total enrolled: 513, Analyzed: 495 (96.5%), Excluded: 18 (state reasons), Recovered: 370 (74.7%), Censored: 125 (25.3%), Median recovery: 5.9 weeks (95% CI: 5.1–6.6). Cross-check every mention of these numbers.

**Authors’ Response**: Thank you very much. We have added a flow diagram showing the selection of the study participants which would help to clarify the sampling procedure. We have consistently rounded the median time of recovery to 5.9 throughout the document. We added it as Additional file: SI 1.

**4. Sample Size Calculation:** Jump from 918→513 unexplained. Add to Methods: *"Initial sample size: 918 (OpenEpi, 1:1 ratio, 95% CI, 80% power, HR=1.59). Since available population was 945 (N<10,000), we applied finite population correction: n_adjusted = (918×945)/(918+945-1) = 466. Adding 10% for incomplete records: final n=513."* Explain why 10% for retrospective review.

**Authors’ Response**: Thank you! We have revised the sample size calculation accordingly. The revised version now reads as:

“*Given that the available study population was limited to 945 individuals, which is below 10,000, we applied the finite population correction formula to adjust the sample size. By employing the standard correction formula which is: Nf=n/(1+[n⁄N]), =918/(1+[918⁄945]. Then, we calculated the final sample size to be 466. After accounting for a 10% non-response rate due to missing values and incomplete patient records, given that the data were collected from records. The final sample size was determined to be 513.*”

**5. Table 5 Verification:** Recovered + Censored ≠ expected totals in multiple rows. Verify EVERY row: Recovered + Censored = Total, Example: Married: 292+85=377 ✓, Fix "Distance from hymen" totals

**Authors’ Response**: Thank you very much! We greatly appreciate the depth of your review and your insightful observations. We acknowledge that there was a typographical error where we mistakenly interchanged the positions for the "No VVF" row between "recovered" and "censored." We have verified all other entries and confirmed their accuracy. All cells now add 370 for recovered, 125 for censored, and a total of 495.

**6. Patient Selection Flowchart**

Create Figure 0 (CONSORT-style):

Total OBF patients (n=945)

↓

Random sampling (n=513)

↓

Excluded incomplete records (n=18)

• Missing admission date: n= 0

• Missing discharge date: n= 6

• Missing outcome: n= 14 (Incomplete data were the main reason for exclusion including discharge date)

↓

Final sample included in the study (n=495)

↓

├─ Recovered (n=370, 74.7%)

├─ Censored-alive (n= 108 not recovered)

├─ Censored-unknown (n= 15 lost to follow-up and 2 transferred out)

└─ Died (n=0)

**Author’s Response**: Thank you very much! We have included the figure showing the flow chart of the selection and reporting of study participants as a supplementary file along with this submission. Those cases included Fistula from Rape 2 girls and one male child. The most common reasons for exclusion were multiple incomplete data including date of discharge and non-obstetric causes. We excluded some of the non-obstetric causes of Fistula treated at Hamlin Fistula Center. No death was reported among the selected participants. Here is the diagram below.

Figure SI1: Flow diagram of the selection of study participants and outcomes for the survival analysis of time to recovery from Obstetric Fistula at Hamlin Fistula Hospital Addis Ababa 2023.

**7. Selection Bias Analysis:** Create **Supplementary Table S1** and Add to Discussion: "Comparison revealed [no differences/differences in X], suggesting [minimal/potential] selection bias."

| **Characteristic** | **Included (n=495)** | **Excluded (n=18)** | **p-value** |
| --- | --- | --- | --- |
| Age (mean±SD) |  |  |  |
| VVF (%) |  |  |  |
| Urethral damage (%) |  |  |  |
| Severe scarring (%) |  |  |  |

**Authors’ response:** Thank you very much for your valuable suggestions, which we believe will strengthen the current findings. However, we were unable to retrieve data for the 18 excluded patients. For some of these patients, data were not collected if we found the patient selected through simple random sampling had incomplete data during data collection time. Some of them were excluded because of multiple incomplete information and the causes were non-obstetric for some of them. The non obstetric causes include two girls and one male children after rape cases). We believe that this limitation is unlikely to have a significant impact on the overall results, as a 10% non-response rate was already accounted for in the sample size calculation.

**8. Statistical Reporting Gaps**

**A. Proportional Hazards Testing** Add to Results: "Proportional hazards assumption verified using log-minus-log plots and Schoenfeld residuals. All variables satisfied assumption (p>0.05) except [list violations]. Violating variables were [handled how]."

**Authors’ Response**: Thank you for your suggestions. We have incorporated it in the revised version of the article. The revised version now reads as:

“*The adequacy of the Cox proportional hazard model was evaluated using a log-minus-log plot for each variable prior to entering into the final model. For each covariate incorporated into the model, a plot was used to estimate the recovery period for patients suffering from OBF. The log minus log plot assumption was satisfied for that covariate if the plot demonstrates random parallel or not cross each other’s. Proportional hazards assumption verified using log-minus-log plots and Schoenfeld residuals.*

*On plot checking: Body Mass Index (BMI), place of delivery, outcome of delivery, age at development of fistula in years, got physiotherapy after surgery, got psychotherapy before surgery, got psychotherapy after surgery, length and width of fistula, rectovaginal fistula and status of urethra were predictors that satisfied the PH assumption. Moreover, marital status, occupational status, educational status, distance from hymen, stage of vaginal scarring, fistula fibrosis and surgical approach were satisfied after adjustments made. However, getting ureteral catheterization before surgery, vesico-vaginal fistula and bladder size was removed due to violation of the assumption even after adjustments made*.”

**B. Model Goodness-of-Fit** Add to Results: "Final Cox model showed better fit than null model (Omnibus: χ²=55.12, df=25, p<0.001). Cox-Snell residuals confirmed adequate fit (Figure 4), with observed values following 45° reference line. Minor deviations at residuals >4 reflect fewer long-term observations but don't invalidate model."

**Authors’ Response**: Dear reviewer, we really appreciate your insight into improving our article. We added your suggestions under the result section on page 13 and 14.

**C. Subgroup Sizes in Table 5** Add column showing n for each category, especially for wide CIs (fistula length AHR=5.23, CI: 1.17–12.33).

**Authors’ Response**: We thank you for this suggestion. In Table 5, we have already included the number for the recovered and censored for all categories, which constitute total number or sample for all subgroups. We therefore believe that the subgroup sizes are adequately reported. Adding an additional column for total n would duplicate existing information and may increase table complexity without providing substantial additional value. We would be happy to revise if there are any more suggestions.

**9. Mortality Data**

**Problem:** Deaths buried in "censored" without details. Report: "Of 125 censored, XXX died (XX%), XXX unknown status, XXX incomplete recovery." Split Table 3 "Outcomes": Recovered | Censored-alive | Died Add to Limitations: "Death treated as censoring. If mortality differs by predictors, hazard ratios may be biased. However, death rate was low (XX%). Future studies should use competing risk models."

**Authors’ Response**: Thank you for your suggestion. We have explored the details behind those censored and added their reason. Accordingly, out of 125 censored108 (86.4%) were with incomplete recovery, 15 (12.0%) lost to follow-up, 2 (1.6%) transferred out to other facilities and no death. We have added one sentence about this information to the result section of the article.

“Among the 125 censored patients, 108 (86.4%) had incomplete recovery, 15 (12.0%) were lost to follow‑up, and 2 (1.6%) were transferred to other facilities, with no recorded deaths.”

**10. Context for Lower Recovery Rate:** 74.7% recovery vs. 81–89% at other centers, inadequately explained. **Add to Methods:** "Hamlin serves as Ethiopia's national referral center, receiving complex cases including failed repairs, extensive tissue damage, and prolonged fistula duration from all regions."

**Authors’ response**: Thank you! We are grateful for your guidance and support sharing invaluable suggestions. We accepted your comment and included under the study setting of the methods section on page 5.

**Expand in Discussion:** "Our 74.7% rate is lower than Gondar (88.07%), Jimma (81.4%), and Mekelle (89.33%). This reflects Hamlin Hospital’s referral center role. Our cohort included: 17.2% with prior failed surgery, 10.1% complete urethral destruction, 16.4% with fistula >1 year. Median recovery time (5.9 weeks) matches other centers (5.14–5.19 weeks), suggesting comparable surgical quality. Lower overall rate reflects case-mix, not inferior care."

**Authors’ response**: We are truly grateful for your insightful suggestions! We accepted your comment and included them under the discussion section on page 15.

**Add Limitation:** "As tertiary center, findings may not generalize to primary-level repairs."

**Authors’ response**: Thank you very much, we have added under the limitation of the study.

**11. Clinical Implications; Current:** Too generic: be Specific, actionable e.g

"**Implications for optimizing OBF care:**

**1. Scale physiotherapy:** Only 7.3% received physiotherapy despite 85% faster recovery (AHR=1.85). Scaling to all patients could improve recovery from 74.7% to [estimate]. Address barriers through: training staff, low-cost alternatives (self-administered exercises), community health worker support.

**2. Counsel complex cases:** Women with urethral destruction (10.1%) recover 57% slower. Set realistic expectations (8 vs. 5.4 weeks) and organize support groups.

**3. Triage resources:** Identify high-risk patients (severe scarring, 17.6%) at admission for prioritized physiotherapy, extended hospitalization, nutritional support.

**4. Research priorities:** Confirm fistula size effect (AHR=5.23, wide CI) in larger studies. Develop standardized severity classification.

**5. Policy shift:** Ethiopia's 2030 OBF elimination requires shifting from surgical access to post-operative care quality. <8% receiving evidence-based physiotherapy shows critical gap."

**Authors’ Response**: Thank you! We have incorporated your comment in the revised version of the manuscript. We have included under the implication and recommendation part of the conclusion on page 13 and 14. We did not include the policy shift statement because it was already considered in the existing Elimination strategic plan 2030.

**12. Expand Limitations:** Add

- **Retrospective:** Missing confounders (nutrition, wealth, distance, surgeon experience, year)
- **Temporal:** 2017–2023 protocols may have evolved
- **Single-center:** Tertiary referral, may not generalize
- **Recovery definition:** Measured continence only, not psychological/social dimensions
- **Excluded cases:** 18 (3.5%) may represent most complex

**Authors’ Response:** Thank you, we appreciate your constructive suggestions. We have already included some of them and revised others according to your suggestions. Data were collected from patients treated since 2017, during which we didn’t observe change in the management of obstetric fistula or protocol. Therefore, we did not consider the change in protocols as a limitation for the current study. As explained above in the diagram most of the excluded patients have multiple incomplete data including the discharge date and for some non-obstetric causes were identified. Additionally, a 10% non-response rate was incorporated into the sample size calculation, given that data were extracted from patient records selected using simple random sampling. We therefore believe that the exclusion of 18 patients is unlikely to have a significant impact on the study findings.

The limitation section now read as:

***“****As strengths the current study included data from randomly selected patients treated over seven years at a specialized fistula center and used survival analysis for time to recovery and its predictors, strengthening the reliability of findings. However, this study has several limitations. The study was based on retrospective design using secondary data, which limits accuracy and completeness of information from records. There is potential bias from incomplete documentation, data quality or inconsistent clinical recording practices across time. Furthermore, potential predictors for OBF such as household wealth, sociocultural factors, nutritional status, distance from health facilities, antenatal care follow-up, psychosocial supports and providers skill and other related factors were not collected in this study. The absence of these factors on patients’ chart may have introduced unmeasured confounding, limiting ability to fully adjust for potential determinants influencing the time to recovery time from OBF. Additionally, since the study included only patients who received surgical treatment and had complete follow-up data, selection bias is possible, as women without care or incomplete records were excluded. As the current study was conducted in one tertiary center, findings may not reflect the situation at primary-level repairs. The current study used continence only to consider OBF patients as recovered and no other dimensions of health such as psychosocial dimensions. This may affect generalizability of findings to all OBF patients in Ethiopia.****”***

**13. Improve Abstract**

**Restructure as:**

**Background:** Start with human impact, state knowledge gap, study aim.

**Methods:** Concise study design, analysis approach (K-M, Cox regression).

**Results:** Lead with recovery rate/time, then key predictors. **Highlight physiotherapy finding upfront.**

**Conclusions:** Specific recommendations (scale physiotherapy, counsel complex cases, individualized rehabilitation).

**Authors’ Response**: Dear Reviewer, we appreciate your suggestions. We did not restructure the Abstract section into different parts as per your earlier comment because the editorial office advised us to remove those divisions. However, we believe the current format still reflects that structure, even though we did not label the sections explicitly. We have now shortened the Methods section by removing a sentence and have revised the Recommendation section accordingly. The Background was crafted with consideration of human impact, highlighting the knowledge gaps and aims of the study.

**14. Comparison Table**

Create **Supplementary Table S2** comparing all Ethiopian OBF studies:

| **Study** | **Location** | **Sample** | **Recovery Rate** | **Median Time** | **Notes** |
| --- | --- | --- | --- | --- | --- |
| Current study findings | Hamlin | 495 | 74.7% | 5.9 weeks | Tertiary referral |
| Derso EA et al., 2020 | Gondar | 289 | 73.7% | 5.19 weeks |  |
| Yismaw L et al., | Gondar University | 612 | 88.07% | 5.14 weeks |  |
| Hussen S et al., 2017 | Harar | 433 | 67.21% | 2.67 weeks | Mean, not median |
| Bihon Am et al., 2022 | Mekelle | 228 | 89.33% | 4.7 weeks | Mean 6 weeks |
| Areba et al., 2022 | Jimma University | 270 | 81.4% | --- | Not reported |

Reference in Discussion.

**Authors’ Response**: Thank you! We truly appreciate your suggestions and time in compiling this data into table to simplify our revision. We will upload it as a supplementary file accordingly. We have added it as “SI 3: Table comparing findings with other Ethiopian studies”

**QUESTIONS TO ANSWER**

1. How many died of the 125 censored?

**Author’s response**: Thank you! There was no death reported among the included participants. We have added this statement under our result section. “Among the 125 censored patients, 108 (86.4%) had incomplete recovery, 15 (12.0%) were lost to follow‑up, and 2 (1.6%) were transferred to other facilities, with no recorded deaths.”

2. Why were 18 records incomplete? (breakdown by reason)

**Author’s response:** Thank you for requesting us to clarify on this issue. The most common reasons for exclusion for those 18 selected individuals were their records had multiple incomplete data from their records including discharge date and for three of them the causes were non-obstetric (due to rape case referred to the center).

3. Clinical criteria for "recovery" at Hamlin?

**Author’s response:** The current study used diagnoses made by clinicians working at Hamlin Fistula Hospital. Recovery was considered when there was a complete restoration of continence following successful closure of the fistula, with no related complications.

4. Did surgical protocols change 2017–2023?

**Author’s response**: There was no observed change in the surgical protocol at the Hamlin Fistula Hospital during our data collection period (2017 to 2023).

5. Year-stratified recovery rates?

**Author’s response:** We did not perform a year‑stratified recovery rate analysis. We believe that no major changes occurred in the services provided at Hamlin Fistula Hospital during the 2017 to 2023 study period that would result in significant variations in recovery rates across the years.

6. Current physiotherapy rate (2024–2025)?

**Author’s response:** Thank you for this important question. It is highly relevant for assessing the implementation of the study findings within the hospital and determining whether they have been translated into practice. However, we did not assess physiotherapy status after the data collection period. This study was conducted as part of the first author’s thesis, so we could not get opportunity to reassess the changes.

7. Prior surgeries at Hamlin vs. elsewhere (of 85)?

**Author’s response:** This data represents the number of obstetric fistula patients who had undergone previous surgical repairs prior to the current procedure. The previous surgeries could have been performed at any fistula treatment centers in Ethiopia, including Addis Ababa Hamlin Fistula Hospital. Overall, 85 (17.2%) of the patients had a history of previous surgery, while 410 (82.8%) had no such history. Out of those who had previous surgery 62 (72.9%) of them had one previous surgery and the rest had more than two surgeries (described in Table 3).

8. Findings shared with Hamlin administration? Response?

**Author’s response:** Thank you for this question. We appreciate your interest in seeing the study findings translated into practice. A copy of the full thesis was submitted to the Research Directorate of Addis Ababa Hamlin Fistula Center following the thesis defense. The Directorate acknowledged receipt of the submission; however, we have not yet received formal feedback. They invited the first author through phone call to present the findings at their upcoming conference scheduled in June 2026.

**Reviewer 3**

- These number of fistula were noted in how many years, please specify.

**Author’s Response**: Thank you very much! Now we have made it clear by revising the statement. The data was taken from Deribe et al., 20202, they used statistical mapping models using 2005 and 2016 Ethiopia Demographic Health Surveys data combined to estimate those numbers.

The new statement now read:

“In Ethiopia, the lifetime estimated risk of obstetric fistula in 2016 indicated that 72,533 women of reproductive age had experienced this condition.”

Thank you very for reviewing our article and your valuable feedback.
