# Supplementary material for: Time to recovery from Obstetric Fistula and its predictors among patients admitted to Hamlin Fistula Center, Addis Ababa, Ethiopia: A survival analysis
Source: PLOS Glob Public Health. 2026 Jul 9;6(7):e0006848. doi: 10.1371/journal.pgph.0006848 (PMC13349304; doi:10.1371/journal.pgph.0006848)
Supplement: Attachment — Submitted filename: OBF_Review_PGPH-D-25-02015_R2_Nkhata.docx [file pgph.0006848.s009.docx]

**PGPH-D-25-02015 R2: Recommendation: Accept Pending Minor Revisions**

*Time to recovery from Obstetric Fistula and its Predictors among patients admitted to Hamlin Fistula Center, Addis Ababa, Ethiopia: A Survival Analysis*

The survival analysis provides highly valuable, actionable insights for clinical care at fistula centers. To enhance the clarity, precision, and impact of your findings before publication, please address the following statistical and interpretive points regarding your multi-variable model results:

| **Category** | **Issue** | **Detail** | **Required action** |
| --- | --- | --- | --- |
| **Critical revisions** | | | |
| Abstract error | Physiotherapy AHR misreported | Abstract states AHR: 1.13 — the CI lower bound used as the point estimate. | Correct to AHR: 1.85, 95% CI: 1.13–3.02. Verify no other abstract statistics are similarly misreported. |
| Table 5 | Formatting & transposed values | AHR "2.60" misplaced outside CI column; "No VVF" recovered/censored counts transposed; row header splits across page break. | Fix cell placement; verify recovered + censored = 495 per row; keep row headers on one page. |
| Table 3 | Track-change artefacts remain | Highlighted cells show both corrected and uncorrected values side-by-side. | Resolve all tracked changes. Verify percentages use correct denominator (n=85 for previous surgery subgroup, not n=495). |
| Conclusions | Duplicate text | Paragraphs appear twice before and after tracked edits. | Remove all duplicated sentences; ensure conclusions flow logically. |
| **Minor revisions** | | | |
| Typos | Three recurring errors | Line 246: "p-value = 045" (missing decimal). Multiple instances of "long rank" throughout. | Correct to "p-value = 0.045"; replace all "long rank" with "log-rank". |
| Sample size | Calculation step not shown | Jump from n=466 to n=513 is arithmetically unclear (lines 123–127). | Show explicitly: 466 × 1.10 ≈ 513. Clarify this accounts for missing records in retrospective design. |
| Inconsistency | Median recovery time varies | "5.86" and "5.9" weeks used interchangeably across text and discussion. | Standardise to 5.9 weeks throughout the clean manuscript. |
| Reference 27 | Incomplete citation | Listed only as bare URL "http://hamlin.org.au/" — insufficient for peer-reviewed citation. | Replace with formal citation (author, year, title, publisher) or official Hamlin annual report. |
| **Editorial / discussion points** | | | |
| Key finding | Physiotherapy gap undersold | Only 7.3% received physiotherapy despite 85% faster recovery (AHR=1.85). Contrast not prominent enough. | State gap prominently in abstract and discussion; frame as key modifiable clinical lever. |
| Interpretation | Wide CI on fistula length | AHR 5.23 (95% CI: 1.17–12.33) rests on small reference group (n=76); estimate is imprecise. | Note in discussion as hypothesis-generating; flag subgroup size in limitations. |
| Discussion | Delayed-recovery profile fragmented | Three risk factors discussed separately: complete urethral distraction (AHR=0.43), severe vaginal scarring (AHR=0.60), rectovaginal fistula (AHR=0.61). | Group to paint a unified clinical picture of patients needing specialised, individualised care. |
| Discussion | Recovery definition narrow | Recovery defined only as restoration of continence; psychosocial dimensions not contextualised. | Add 1–2 sentences linking to WHO multidimensional health concept and holistic fistula outcome literature. |
| Language | Copyedit needed | Minor grammatical issues persist (tense inconsistency, run-on sentences). PLOS does not copyedit accepted manuscripts. | Request a final professional English language edit, focusing on Methods and Discussion. |
