# Supplementary material for: Time to recovery from Obstetric Fistula and its predictors among patients admitted to Hamlin Fistula Center, Addis Ababa, Ethiopia: A survival analysis
Source: PLOS Glob Public Health. 2026 Jul 9;6(7):e0006848. doi: 10.1371/journal.pgph.0006848 (PMC13349304; doi:10.1371/journal.pgph.0006848)
Supplement: Attachment — Submitted filename: V3 Response to Reviewers Comment.docx [file pgph.0006848.s011.docx]

**PGPH-D-25-02015 R2: Recommendation: Accept Pending Minor Revisions**

*Time to recovery from Obstetric Fistula and its Predictors among patients admitted to Hamlin Fistula Center, Addis Ababa, Ethiopia: A Survival Analysis*

The survival analysis provides highly valuable, actionable insights for clinical care at fistula centers. To enhance the clarity, precision, and impact of your findings before publication, please address the following statistical and interpretive points regarding your multi-variable model results:

**Authors response:** Thank you very much for providing us with your constructive feedback.

| **Category** | **Issue** | **Detail** | **Required action** | **Response to reviewer comments** |
| --- | --- | --- | --- | --- |
| **Critical revisions** | | | |  |
| Abstract error | Physiotherapy AHR misreported | Abstract states AHR: 1.13 — the CI lower bound used as the point estimate. | Correct to AHR: 1.85, 95% CI: 1.13–3.02. Verify no other abstract statistics are similarly misreported. | Dear Reviewer thank you so much. This is a nice observation. We have corrected and also verified for all results. |
| Table 5 | Formatting & transposed values | AHR "2.60" misplaced outside CI column; "No VVF" recovered/censored counts transposed; row header splits across page break. | Fix cell placement; verify recovered + censored = 495 per row; keep row headers on one page. | Thank you! We had checked and revised the whole table 5 cells. We revised the p-value through adding 0 before the decimal places. Corrected the mistakenly put p-value “2.60” to 0.260. |
| Table 3 | Track-change artefacts remain | Highlighted cells show both corrected and uncorrected values side-by-side. | Resolve all tracked changes. Verify percentages use correct denominator (n=85 for previous surgery subgroup, not n=495). | Thank you! We have checked the data and found it was correct on the clean version of our main document. The denominator was 85 for the frequency of previous surgery. The artefacts remained on the tracked change only. |
| Conclusions | Duplicate text | Paragraphs appear twice before and after tracked edits. | Remove all duplicated sentences; ensure conclusions flow logically. | Thank you! We have checked and it appears there are no tracked changes in our clean version of the main document. |
| **Minor revisions** | | | |  |
| Typos | Three recurring errors | Line 246: "p-value = 045" (missing decimal). Multiple instances of "long rank" throughout. | Correct to "p-value = 0.045"; replace all "long rank" with "log-rank". | That’s a great observation; we have corrected it accordingly in the revised version. |
| Sample size | Calculation step not shown | Jump from n=466 to n=513 is arithmetically unclear (lines 123–127). | Show explicitly: 466 × 1.10 ≈ 513. Clarify this accounts for missing records in retrospective design. | Thank you very much, we revised it to: “After accounting for a 10% non-response rate due to missing values and incomplete patient records (as the data were collected from records), the final sample size was determined to be 466 x 1.10, which is approximately 513.” |
| Inconsistency | Median recovery time varies | "5.86" and "5.9" weeks used interchangeably across text and discussion. | Standardise to 5.9 weeks throughout the clean manuscript. | Thank you, we have checked this in the clean version of the manuscript, and we found consistently used 5.9 weeks as a median time of recovery from the obstetric fistula. |
| Reference 27 | Incomplete citation | Listed only as bare URL "http://hamlin.org.au/" — insufficient for peer-reviewed citation. | Replace with formal citation (author, year, title, publisher) or official Hamlin annual report. | Thank you very much, we modified the reference accordingly. We revised it to: “Cathrine Hamlin Fistula Foundation. Hamlin Fistula Hospital Addis Ababa Impact Report June 2025 [Internet]. Addis Ababa; 2025. Available from: https://hamlinfistula.org/en/” |
| **Editorial / discussion points** | | | |  |
| Key finding | Physiotherapy gap undersold | Only 7.3% received physiotherapy despite 85% faster recovery (AHR=1.85). Contrast not prominent enough. | State gap prominently in abstract and discussion; frame as key modifiable clinical lever. | Thank you! We added one statement in the abstract and also added this to the discussion section. “In this study, only 7.3% of OBF patients received physiotherapy despite 85% faster recovery. This suggests that physiotherapy is an important clinical intervention that could substantially improve recovery outcomes if integrated into routine post operative OBF patients care.” We revised also to the implication section.  Additionally, we revised the first paragraph of discussion adding the key findings. “The study found that the median time of recovery for fistula patients was 5.9 weeks (95%CI=5.1,6.6) among OBF patients in Hamlin fistula center Addis Ababa. The time to recovery from OBF was significantly affected by predictors such as physiotherapy after surgery, VVF length, urethral status, vaginal scaring stage, and rectal fistula fibrosis.” |
| Interpretation | Wide CI on fistula length | AHR 5.23 (95% CI: 1.17–12.33) rests on small reference group (n=76); estimate is imprecise. | Note in discussion as hypothesis-generating; flag subgroup size in limitations. | Thank you we have included under the limitation section. The added statement reads: “The wide confidence interval for length of VVF (AHR: 5.23, 95% CI: 1.17–12.33) may have limited the precision of the estimated effect, and the result should be interpreted with caution.” |
| Discussion | Delayed-recovery profile fragmented | Three risk factors discussed separately: complete urethral distraction (AHR=0.43), severe vaginal scarring (AHR=0.60), rectovaginal fistula (AHR=0.61). | Group to paint a unified clinical picture of patients needing specialised, individualised care. | Thank you, we have revised the whole discussion section accordingly. |
| Discussion | Recovery definition narrow | Recovery defined only as restoration of continence; psychosocial dimensions not contextualised. | Add 1–2 sentences linking to WHO multidimensional health concept and holistic fistula outcome literature. | Thank you we have revised this under the limitations section of the discussion. The new statement now read; “*The current study used physical repair and continence only to consider OBF patients as recovered and did not consider the World Health Organizations multidimensional outcomes of health such as psychological wellbeing, social re-integration and functional or economic restorations (35)*.” Also added one citation for the “WHO Obstetric Fistula Guiding Principles ….” Into the reference list. |
| Language | Copyedit needed | Minor grammatical issues persist (tense inconsistency, run-on sentences). PLOS does not copyedit accepted manuscripts. | Request a final professional English language edit, focusing on Methods and Discussion. | The authors have carefully reviewed and revised the final manuscript. In addition, the entire manuscript was critically evaluated by a colleague (PhD student) from Australia, and all recommendations provided have been fully incorporated into the revised version. |

Dear Reviewer,

We sincerely appreciate your valuable comments and insightful suggestions on our manuscript. Thank you for taking the time to carefully review our work and for your constructive feedback, which has significantly contributed to improving the quality and strength of the manuscript.
