# Supplementary material for: Time to recovery from Obstetric Fistula and its predictors among patients admitted to Hamlin Fistula Center, Addis Ababa, Ethiopia: A survival analysis
Source: PLOS Glob Public Health. 2026 Jul 9;6(7):e0006848. doi: 10.1371/journal.pgph.0006848 (PMC13349304; doi:10.1371/journal.pgph.0006848)
Supplement: Peer review history

# Peer review: Decision Letter 0

**DOI:** 10.1371/journal.pgph.0006848.r001

**Authors:** Sarah Jose

17 Sep 2025

PGPH-D-25-02015

Time to recovery from Obstetric Fistula and its Predictors among Obstetric Fistula patients admitted to Hamlin Fistula Center, Addis Ababa, Ethiopia: A Survival Analysis.

PLOS Global Public Health

Dear Dr. Bulto,

Thank you for submitting your manuscript to PLOS Global Public Health. After careful consideration, we feel that it has merit but does not fully meet PLOS Global Public Health’s publication criteria as it currently stands. Therefore, we invite you to submit a revised version of the manuscript that addresses the points raised during the review process.

Please carefully review the reviewers' comments and revise your manuscript accordingly, providing a point-by-point response to the reviewers upon resubmission.

Please submit your revised manuscript by Oct 31 2025 11:59PM. If you will need more time than this to complete your revisions, please reply to this message or contact the journal office at globalpubhealth@plos.org. When you're ready to submit your revision, log on to https://www.editorialmanager.com/pgph/ and select the 'Submissions Needing Revision' folder to locate your manuscript file.

Please include the following items when submitting your revised manuscript:

- A rebuttal letter that responds to each point raised by the editor and reviewer(s). You should upload this letter as a separate file labeled 'Response to Reviewers'.
- A marked-up copy of your manuscript that highlights changes made to the original version. You should upload this as a separate file labeled 'Revised Manuscript with Track Changes'.
- An unmarked version of your revised paper without tracked changes. You should upload this as a separate file labeled 'Manuscript'.

Guidelines for resubmitting your figure files are available below the reviewer comments at the end of this letter.

We look forward to receiving your revised manuscript.

Kind regards,

Sarah Jose, Ph.D.

Staff Editor

PLOS Global Public Health

Journal Requirements:

If the reviewer comments include a recommendation to cite specific previously published works, please review and evaluate these publications to determine whether they are relevant and should be cited. There is no requirement to cite these works unless the editor has indicated otherwise.

Additional Editor Comments (if provided):

Reviewer #1:

Reviewer #2:

[Note: HTML markup is below. Please do not edit.]

Reviewers' comments:

Reviewer's Responses to Questions

**Comments to the Author**

1. Does this manuscript meet PLOS Global Public Health’s publication criteria? Is the manuscript technically sound, and do the data support the conclusions? The manuscript must describe methodologically and ethically rigorous research with conclusions that are appropriately drawn based on the data presented.

Reviewer #1: Yes

Reviewer #2: Yes

**********

2. Has the statistical analysis been performed appropriately and rigorously?

Reviewer #1: Yes

Reviewer #2: No

**********

3. Have the authors made all data underlying the findings in their manuscript fully available (please refer to the Data Availability Statement at the start of the manuscript PDF file)?

The PLOS Data policy requires authors to make all data underlying the findings described in their manuscript fully available without restriction, with rare exception. The data should be provided as part of the manuscript or its supporting information, or deposited to a public repository. For example, in addition to summary statistics, the data points behind means, medians and variance measures should be available. If there are restrictions on publicly sharing data—e.g. participant privacy or use of data from a third party—those must be specified.

Reviewer #1: No

Reviewer #2: Yes

**********

4. Is the manuscript presented in an intelligible fashion and written in standard English?

PLOS Global Public Health does not copyedit accepted manuscripts, so the language in submitted articles must be clear, correct, and unambiguous. Any typographical or grammatical errors should be corrected at revision, so please note any specific errors here.

Reviewer #1: Yes

Reviewer #2: Yes

**********

5. Review Comments to the Author

Please use the space provided to explain your answers to the questions above. You may also include additional comments for the author, including concerns about dual publication, research ethics, or publication ethics. (Please upload your review as an attachment if it exceeds 20,000 characters)

Reviewer #1: First of all, I would like to sincerely thank the editor for inviting me to review the important paper titled " Time to recovery from Obstetric Fistula and its Predictors among Obstetric Fistula patients admitted to Hamlin Fistula Center, Addis Ababa, Ethiopia: A Survival Analysis." I also extend my appreciation to the author for providing such interesting and valuable evidence on an issue that is increasingly significant in developing countries like Ethiopia.

Here is a suggested revision for your comments section:

General comments:

1. The paper is well written and organized, though some modifications are advised.

2. From my search there are a lot of papers that say what is new for this paper in Ethiopia…

3. Attention is needed to improve sentence synthesis and grammar throughout the manuscript.

Specific Comments:

Abstract:

Well written and organized, but please put the subtitles, like "methods," "result," and "conclusion and recommendation."

Please be specific about your recommendation.

Introduction: Really, this section is also well written and organized. If possible, please include why this problem persists today, what has been done, what strategies are in place, what obstacles remain, and what the future implications for SGD are.

Methods: operational definition: Survival time: the time period from a starting treatment for obstetric fistula to recovery in weeks. Please, could you give the standard or duration follow-up?

Include the overall goodness-of-fit value of the model.

Since the issue is sensitive, you need to provide the IRB number.

Result: Well stated.

1. In such a study, it's advised to run the frequency both for the outcome and the censored with the present clearly.

Discussion: from your result "this result was lower than studies conducted in other parts of Ethiopia such as: at

299 Gondar University Teaching (88.07%) (20), Jimma University Medical Center (81.4%) (25) and 300 Mekele Hamlin Fistula Center (89.33%) " AA, the capital city where advanced medical procedures may probably be given… Please revise your justification.

Please add implication after justification of all discussion points…

Reviewer #2: Thank you for the opportunity to review this retrospective cohort study on recovery time and predictors of obstetric fistula at Hamlin Fistula Center. The study tackles a critical maternal health issue with a robust dataset and appropriate survival analysis, but several methodological, stylistic, and presentation aspects require refinement. With clearer methods, stronger narrative coherence, and a more human-centred framing, it has the potential to make a meaningful contribution to maternal health and provide valuable insights for clinicians, policymakers, and women’s health advocates.

Section-by-Section Review

1. Abstract: There are inconsistent confidence interval (CI) formatting (commas vs. colons), fragmented presentation of results, and limited narrative flow. The abstract omits the human dimension and clinical implications of obstetric fistula (OBF).

a. Standardise statistical reporting for CIs.

b. Begin with a compelling sentence highlighting the human cost of OBF.

c. Present results in a coherent narrative rather than disjointed statistics.

d. Conclude with a statement on how findings could inform patient care, rehabilitation, or health policy.

2. Introduction: this section is informative but overly dense. Transition from global to local context is abrupt. The human experience of OBF pain, stigma, and isolation has been underemphasised.

a. Condense global statistics; shift focus towards Ethiopia’s context.

b. Integrate a paragraph on the psychosocial toll of OBF to humanise the issue.

c. Where ethically permissible, include patient quotes or anecdotal reflections to ground the narrative in lived experience.

3. Methods: The Sample size calculation appears mismatched to the study design and there is no explanation of handling missing data or competing risks. Also ethical considerations have been are presented procedurally, lacking empathy.

a. Clarify the rationale for sample size determination and any correction formula used.

b. Explain how missing data and competing risks were assessed and addressed.

c. Reframe ethical considerations to emphasise patient dignity, confidentiality, and sensitivity, not just procedural approvals.

4. Results: Some percentages and raw numbers are inconsistent, Survival curves lack Cis and interpretation of the Cox-Snell residual plots is vague.

a. Audit all numerical data for consistency.

b. Add CIs to survival curves.

c. Provide clearer interpretation of diagnostic plots and model assumptions.

5. Discussion: there is limited engagement with study limitations, superficial comparisons with other studies, and underdeveloped clinical implications.

a. Expand discussion of limitations, including retrospective bias, unmeasured confounders, and selection effects.

b. When comparing with other studies, critique methodological differences and their influence on findings.

c. Strengthen clinical implications: highlight how results may guide post-operative care, physiotherapy, counselling, and community reintegration.

6. Tables and Figures: there is inconsistent formatting of the tables and figures lack clarity and polish. There is also no flow diagram of patient selection.

a. Standardise table formatting, labelling, and style.

b. Improve figure resolution and add clear, descriptive captions.

c. Include a flowchart of patient inclusion/exclusion to enhance transparency.

7. Language and Style: there are a lot and frequent grammatical errors, awkward phrasing, inconsistent terminology (“OBF” vs. “obstetric fistula”) which makes readability very difficult.

a. Undertake thorough proofreading or professional language editing.

b. Maintain consistent terminology throughout.

c. Use active voice and empathetic language, particularly when describing patients’ experiences.

8. Human-Centred Enhancements:

a. Incorporate a brief patient vignette (with ethical clearance) to illustrate the journey from injury to recovery.

b. Discuss stigma, gendered vulnerabilities, and social reintegration, not just clinical outcomes.

c. Frame recovery as multidimensional—physical, psychological, and social.

**********

6. PLOS authors have the option to publish the peer review history of their article (what does this mean?). If published, this will include your full peer review and any attached files.

**Do you want your identity to be public for this peer review?**  If you choose “no”, your identity will remain anonymous but your review may still be made public.

For information about this choice, including consent withdrawal, please see our Privacy Policy.

Reviewer #1: No

Reviewer #2: **Yes:**  Loveness Anila Nkhata

**********

[NOTE: If reviewer comments were submitted as an attachment file, they will be attached to this email and accessible via the submission site. Please log into your account, locate the manuscript record, and check for the action link "View Attachments". If this link does not appear, there are no attachment files.]

-->Figure Resubmissions:

While revising your submission, we strongly recommend that you use PLOS’s NAAS tool (https://ngplosjournals.pagemajik.ai/artanalysis) to test your figure files. NAAS can convert your figure files to the TIFF file type and meet basic requirements (such as print size, resolution), or provide you with a report on issues that do not meet our requirements and that NAAS cannot fix.

After uploading your figures to PLOS’s NAAS tool - https://ngplosjournals.pagemajik.ai/artanalysis, NAAS will process the files provided and display the results in the "Uploaded Files" section of the page as the processing is complete. If the uploaded figures meet our requirements (or NAAS is able to fix the files to meet our requirements), the figure will be marked as "fixed" above. If NAAS is unable to fix the files, a red "failed" label will appear above. When NAAS has confirmed that the figure files meet our requirements, please download the file via the download option, and include these NAAS processed figure files when submitting your revised manuscript.

Attachment Submitted filename: Time to Recovery from Obstetric Fistula.docx

# Author comment: Author response to Decision Letter 1

**DOI:** 10.1371/journal.pgph.0006848.r002

1 Dec 2025

Attachment Submitted filename: V1 Response to Reviewers OBF time to recovery Nov 14, 2025.docx

# Peer review: Decision Letter 1

**DOI:** 10.1371/journal.pgph.0006848.r003

**Authors:** Sarah Jose, Rahul Gajbhiye

29 Dec 2025

PGPH-D-25-02015R1

Time to recovery from Obstetric Fistula and its Predictors among Obstetric Fistula patients admitted to Hamlin Fistula Center, Addis Ababa, Ethiopia: A Survival Analysis.

PLOS Global Public Health

Dear Dr. Bulto,

Thank you for submitting your manuscript to PLOS Global Public Health. After careful consideration, we feel that it has merit but does not fully meet PLOS Global Public Health’s publication criteria as it currently stands. Therefore, we invite you to submit a revised version of the manuscript that addresses the points raised during the review process.

Please submit your revised manuscript by Feb 12 2026 11:59PM. If you will need more time than this to complete your revisions, please reply to this message or contact the journal office at globalpubhealth@plos.org. When you're ready to submit your revision, log on to https://www.editorialmanager.com/pgph/ and select the 'Submissions Needing Revision' folder to locate your manuscript file.

Please include the following items when submitting your revised manuscript:

- A letter that responds to each point raised by the editor and reviewer(s). You should upload this letter as a separate file labeled 'Response to Reviewers'.
- A marked-up copy of your manuscript that highlights changes made to the original version. You should upload this as a separate file labeled 'Revised Manuscript with Track Changes'.
- An unmarked version of your revised paper without tracked changes. You should upload this as a separate file labeled 'Manuscript'.

Guidelines for resubmitting your figure files are available below the reviewer comments at the end of this letter.

We look forward to receiving your revised manuscript.

Kind regards,

Rahul Gajbhiye, MBBS PhD

Academic Editor

PLOS Global Public Health

Journal Requirements:

1. If the reviewer comments include a recommendation to cite specific previously published works, please review and evaluate these publications to determine whether they are relevant and should be cited. There is no requirement to cite these works unless the editor has indicated otherwise.

Additional Editor Comments (if provided):

[Note: HTML markup is below. Please do not edit.]

Reviewers' comments:

Reviewer's Responses to Questions

**Comments to the Author**

1. If the authors have adequately addressed your comments raised in a previous round of review and you feel that this manuscript is now acceptable for publication, you may indicate that here to bypass the “Comments to the Author” section, enter your conflict of interest statement in the “Confidential to Editor” section, and submit your "Accept" recommendation.

Reviewer #2: (No Response)

Reviewer #3: (No Response)

**********

2. Does this manuscript meet PLOS Global Public Health’s publication criteria? Is the manuscript technically sound, and do the data support the conclusions? The manuscript must describe methodologically and ethically rigorous research with conclusions that are appropriately drawn based on the data presented.

Reviewer #2: Yes

Reviewer #3: Yes

**********

3. Has the statistical analysis been performed appropriately and rigorously?

Reviewer #2: No

Reviewer #3: Yes

**********

4. Have the authors made all data underlying the findings in their manuscript fully available (please refer to the Data Availability Statement at the start of the manuscript PDF file)?

The PLOS Data policy requires authors to make all data underlying the findings described in their manuscript fully available without restriction, with rare exception. The data should be provided as part of the manuscript or its supporting information, or deposited to a public repository. For example, in addition to summary statistics, the data points behind means, medians and variance measures should be available. If there are restrictions on publicly sharing data—e.g. participant privacy or use of data from a third party—those must be specified.

Reviewer #2: Yes

Reviewer #3: Yes

**********

5. Is the manuscript presented in an intelligible fashion and written in standard English?

PLOS Global Public Health does not copyedit accepted manuscripts, so the language in submitted articles must be clear, correct, and unambiguous. Any typographical or grammatical errors should be corrected at revision, so please note any specific errors here.

Reviewer #2: Yes

Reviewer #3: Yes

**********

6. Review Comments to the Author

Please use the space provided to explain your answers to the questions above. You may also include additional comments for the author, including concerns about dual publication, research ethics, or publication ethics. (Please upload your review as an attachment if it exceeds 20,000 characters)

Reviewer #2: Refer to attached file

Reviewer #3: manuscript is suitable for publication with minor changes. comment is inserted in attached pdf file

**********

7. PLOS authors have the option to publish the peer review history of their article (what does this mean?). If published, this will include your full peer review and any attached files.

**Do you want your identity to be public for this peer review?**  If you choose “no”, your identity will remain anonymous but your review may still be made public.

For information about this choice, including consent withdrawal, please see our Privacy Policy.

Reviewer #2: **Yes:**  Dr Loveness A. Nkhata

Reviewer #3: No

**********

[NOTE: If reviewer comments were submitted as an attachment file, they will be attached to this email and accessible via the submission site. Please log into your account, locate the manuscript record, and check for the action link "View Attachments". If this link does not appear, there are no attachment files.]

-->Figure Resubmissions:

While revising your submission, we strongly recommend that you use PLOS’s NAAS tool (https://ngplosjournals.pagemajik.ai/artanalysis) to test your figure files. NAAS can convert your figure files to the TIFF file type and meet basic requirements (such as print size, resolution), or provide you with a report on issues that do not meet our requirements and that NAAS cannot fix.

After uploading your figures to PLOS’s NAAS tool - https://ngplosjournals.pagemajik.ai/artanalysis, NAAS will process the files provided and display the results in the "Uploaded Files" section of the page as the processing is complete. If the uploaded figures meet our requirements (or NAAS is able to fix the files to meet our requirements), the figure will be marked as "fixed" above. If NAAS is unable to fix the files, a red "failed" label will appear above. When NAAS has confirmed that the figure files meet our requirements, please download the file via the download option, and include these NAAS processed figure files when submitting your revised manuscript.

Attachment Submitted filename: Dr Nkhatas report.docx

Attachment Submitted filename: PGPH-D-25-02015_R1 (1).pdf

# Author comment: Author response to Decision Letter 2

**DOI:** 10.1371/journal.pgph.0006848.r004

18 May 2026

Attachment Submitted filename: V2 Response to Reviewers Comment.docx

# Peer review: Decision Letter 2

**DOI:** 10.1371/journal.pgph.0006848.r005

**Authors:** Sarah Jose, Rahul Gajbhiye, Emma Campbell

2 Jun 2026

PGPH-D-25-02015R2

Time to recovery from Obstetric Fistula and its Predictors among Obstetric Fistula patients admitted to Hamlin Fistula Center, Addis Ababa, Ethiopia: A Survival Analysis

PLOS Global Public Health

Dear Dr. Bulto,

Thank you for submitting your manuscript to PLOS Global Public Health. After careful consideration, we feel that it has merit but does not fully meet PLOS Global Public Health’s publication criteria as it currently stands. Therefore, we invite you to submit a revised version of the manuscript that addresses the points raised during the review process.

The reviewer has provided some additional comments that need to be addressed prior to publication, including a request to copy edit your manuscript. Please ensure all concerns are addressed before you resubmit.

Please submit your revised manuscript by Jul 02 2026 11:59PM. If you will need more time than this to complete your revisions, please reply to this message or contact the journal office at globalpubhealth@plos.org. When you're ready to submit your revision, log on to https://www.editorialmanager.com/pgph/ and select the 'Submissions Needing Revision' folder to locate your manuscript file.

Please include the following items when submitting your revised manuscript:

- A letter that responds to each point raised by the editor and reviewer(s). You should upload this letter as a separate file labeled 'Response to Reviewers'.
- A marked-up copy of your manuscript that highlights changes made to the original version. You should upload this as a separate file labeled 'Revised Manuscript with Track Changes'.
- An unmarked version of your revised paper without tracked changes. You should upload this as a separate file labeled 'Manuscript'.

Guidelines for resubmitting your figure files are available below the reviewer comments at the end of this letter.

As the corresponding author, your ORCID iD is verified in the submission system and will appear in the published article. PLOS supports the use of ORCID, and we encourage all coauthors to register for an ORCID iD and use it as well. Please encourage your coauthors to verify their ORCID iD within the submission system before final acceptance, as unverified ORCID iDs will not appear in the published article. *Only*  the individual author can complete the verification step; PLOS staff *cannot*  verify ORCID iDs on behalf of authors.

We look forward to receiving your revised manuscript.

Kind regards,

Emma Campbell, Ph.D

Staff Editor

PLOS Global Public Health

**Journal Requirements:**

If the reviewer comments include a recommendation to cite specific previously published works, please review and evaluate these publications to determine whether they are relevant and should be cited. There is no requirement to cite these works unless the editor has indicated otherwise.

Please review your reference list to ensure that it is complete and correct. If you have cited papers that have been retracted, please include the rationale for doing so in the manuscript text, or remove these references and replace them with relevant current references. Any changes to the reference list should be mentioned in the rebuttal letter that accompanies your revised manuscript. If you need to cite a retracted article, indicate the article’s retracted status in the References list and also include a citation and full reference for the retraction notice.

**Additional Editor Comments (if provided):**

[Note: HTML markup is below. Please do not edit.]

Reviewers' comments:

Reviewer's Responses to Questions

**Comments to the Author**

1. If the authors have adequately addressed your comments raised in a previous round of review and you feel that this manuscript is now acceptable for publication, you may indicate that here to bypass the “Comments to the Author” section, enter your conflict of interest statement in the “Confidential to Editor” section, and submit your "Accept" recommendation.

Reviewer #2: All comments have been addressed

**********

2. Does this manuscript meet PLOS Global Public Health’s publication criteria? Is the manuscript technically sound, and do the data support the conclusions? The manuscript must describe methodologically and ethically rigorous research with conclusions that are appropriately drawn based on the data presented.

Reviewer #2: Yes

**********

3. Has the statistical analysis been performed appropriately and rigorously?

Reviewer #2: Yes

**********

4. Have the authors made all data underlying the findings in their manuscript fully available (please refer to the Data Availability Statement at the start of the manuscript PDF file)?

The PLOS Data policy requires authors to make all data underlying the findings described in their manuscript fully available without restriction, with rare exception. The data should be provided as part of the manuscript or its supporting information, or deposited to a public repository. For example, in addition to summary statistics, the data points behind means, medians and variance measures should be available. If there are restrictions on publicly sharing data—e.g. participant privacy or use of data from a third party—those must be specified.

Reviewer #2: Yes

**********

5. Is the manuscript presented in an intelligible fashion and written in standard English?

PLOS Global Public Health does not copyedit accepted manuscripts, so the language in submitted articles must be clear, correct, and unambiguous. Any typographical or grammatical errors should be corrected at revision, so please note any specific errors here.

Reviewer #2: Yes

**********

6. Review Comments to the Author

Please use the space provided to explain your answers to the questions above. You may also include additional comments for the author, including concerns about dual publication, research ethics, or publication ethics. (Please upload your review as an attachment if it exceeds 20,000 characters)

**Reviewer #2:**  There are a few concerns highlighted in the attached report that must be addressed before publication

**********

7. PLOS authors have the option to publish the peer review history of their article (what does this mean?). If published, this will include your full peer review and any attached files.

**Do you want your identity to be public for this peer review?**  If you choose “no”, your identity will remain anonymous but your review may still be made public.

For information about this choice, including consent withdrawal, please see our Privacy Policy.

Reviewer #2: **Yes:**  Dr. Loveness Anila Nkhata

**********

[NOTE: If reviewer comments were submitted as an attachment file, they will be attached to this email and accessible via the submission site. Please log into your account, locate the manuscript record, and check for the action link "View Attachments". If this link does not appear, there are no attachment files.]

**Figure Resubmissions:**

While revising your submission, we strongly recommend that you use PLOS’s NAAS tool (https://ngplosjournals.pagemajik.ai/artanalysis) to test your figure files. NAAS can convert your figure files to the TIFF file type and meet basic requirements (such as print size, resolution), or provide you with a report on issues that do not meet our requirements and that NAAS cannot fix.

After uploading your figures to PLOS’s NAAS tool - https://ngplosjournals.pagemajik.ai/artanalysis, NAAS will process the files provided and display the results in the "Uploaded Files" section of the page as the processing is complete. If the uploaded figures meet our requirements (or NAAS is able to fix the files to meet our requirements), the figure will be marked as "fixed" above. If NAAS is unable to fix the files, a red "failed" label will appear above. When NAAS has confirmed that the figure files meet our requirements, please download the file via the download option, and include these NAAS processed figure files when submitting your revised manuscript.

Attachment Submitted filename: OBF_Review_PGPH-D-25-02015_R2_Nkhata.docx

# Author comment: Author response to Decision Letter 3

**DOI:** 10.1371/journal.pgph.0006848.r006

21 Jun 2026

Attachment Submitted filename: V3 Response to Reviewers Comment.docx

# Editor report: Decision Letter 3

**DOI:** 10.1371/journal.pgph.0006848.r007

**Authors:** Sarah Jose, Rahul Gajbhiye, Emma Campbell, Julia Robinson

24 Jun 2026

Time to recovery from Obstetric Fistula and its Predictors among patients admitted to Hamlin Fistula Center, Addis Ababa, Ethiopia: A Survival Analysis

PGPH-D-25-02015R3

Dear Bulto,

We are pleased to inform you that your manuscript 'Time to recovery from Obstetric Fistula and its Predictors among patients admitted to Hamlin Fistula Center, Addis Ababa, Ethiopia: A Survival Analysis' has been provisionally accepted for publication in PLOS Global Public Health.

Before your manuscript can be formally accepted you will need to complete some formatting changes, which you will receive in a follow up email. A member of our team will be in touch with a set of requests.

Please note that your manuscript will not be scheduled for publication until you have made the required changes, so a swift response is appreciated.

IMPORTANT: The editorial review process is now complete. PLOS will only permit corrections to spelling, formatting or significant scientific errors from this point onwards. Requests for major changes, or any which affect the scientific understanding of your work, will cause delays to the publication date of your manuscript.

If your institution or institutions have a press office, please notify them about your upcoming paper to help maximize its impact. If they'll be preparing press materials, please inform our press team as soon as possible -- no later than 48 hours after receiving the formal acceptance. Your manuscript will remain under strict press embargo until 2 pm Eastern Time on the date of publication. For more information, please contact globalpubhealth@plos.org.

Thank you again for supporting Open Access publishing; we are looking forward to publishing your work in PLOS Global Public Health.

Best regards,

Julia Robinson

Executive Editor

PLOS Global Public Health

***********************************************************

Reviewer Comments (if any, and for reference):
